# Impact of sweet, umami, and bitter taste receptor (*TAS1R* and *TAS2R*) genomic and expression alterations in solid tumors on survival

**DOI:** 10.1038/s41598-022-12788-z

**Published:** 2022-05-27

**Authors:** Ryan M. Carey, TaeBeom Kim, Noam A. Cohen, Robert J. Lee, Kevin T. Nead

**Affiliations:** 1 Department of Otorhinolaryngology – Head and Neck Surgery, Hospital of the University of Pennsylvania, 3400 Spruce Street, 5th floor Ravdin Suite A, Philadelphia, PA 19104 USA; 2 Department of Epidemiology, The University of Texas MD Anderson Cancer Center, Houston, TX USA; 3 Philadelphia Veterans Affairs Medical Center, Philadelphia, PA USA; 4 Department of Physiology, University of Pennsylvania Perelman School of Medicine, Philadelphia, PA USA; 5 Department of Radiation Oncology, The University of Texas MD Anderson Cancer Center, Houston, TX USA

**Keywords:** Cancer, Cancer genetics, Prognostic markers

## Abstract

Originally identified on the tongue for their chemosensory role, the receptors for sweet, umami, and bitter taste are expressed in some cancers where they regulate important cellular processes including apoptosis and proliferation. We examined DNA mutations (n = 5103), structural variation (n = 7545), and expression (n = 6224) of genes encoding sweet or umami receptors (*TAS1R*s) and bitter receptors (*TAS2R*s) in 45 solid tumors subtypes compared to corresponding normal tissue using The Cancer Genome Atlas and the Genotype Tissue Expression Project databases. Expression of *TAS1R* and *TAS2R* genes differed between normal and cancer tissue, and nonsilent mutations occurred in many solid tumor taste receptor genes (~ 1–7%). Expression levels of certain *TAS1R*s/*TAS2R*s were associated with survival differences in 12 solid tumor subtypes. Increased *TAS1R1* expression was associated with improved survival in lung adenocarcinoma (mean survival difference + 1185 days, *p* = 0.0191). Increased *TAS2R14* expression was associated with worse survival in adrenocortical carcinoma (−1757 days, *p* < 0.001) and esophageal adenocarcinoma (−640 days, *p* = 0.0041), but improved survival in non-papillary bladder cancer (+ 343 days, *p* = 0.0436). Certain taste receptor genes may be associated with important oncologic pathways and could serve as biomarkers for disease outcomes.

## Introduction

Sweet and bitter taste receptors, T1Rs and T2Rs respectively, are G-protein-coupled receptors (GPCRs) first identified in type 1 and 2 taste cells on the tongue^1^. The T1R family, and associated *TAS1R* genes, consists of 3 isoforms including *TAS1R1*, *TAS1R2*, and *TAS1R3* located on the short arm of human chromosome 1^1,2^. T1Rs function as heterodimers (i.e. T1R1+3 and T1R2+3) to bind a variety of ligands including sucrose and amino acids for detection of umami (T1R1+3) and sweet (T1R2+3) taste^3,4^. There are 25 human T2R isoforms encoded by *TAS2R* genes located on chromosomes 5, 7, and 12^1,5–7^. T2Rs are activated by bitter compounds and signal through Gα-mediated cAMP decrease, Gβγ-activation of phospholipase C, and downstream calcium (Ca^2^^+^) release^8–11﻿^. T1Rs and T2Rs have considerable genetic variability with many common polymorphisms that influence human taste preferences for foods^11–13^ like green leafy vegetables^14^, coffee^15^, and grapefruit^16^.

T2Rs and T1Rs are expressed in normal tissue outside of the oral cavity including in the airway epithelia^17,18^, thyroid^19^, lung^20^, and gastrointestinal (GI) tract^21,22^, where they have a diverse array of functions. Nasal epithelial cells express functional T2Rs that bind bacterial quorum-sensing molecules to activate Ca^2+^-mediated nitric oxide production to clear pathogens^23^. T1Rs in specialized airway solitary chemosensory cells sense changes in airway surface liquid glucose concentration to modulate bactericidal T2R responses^17,18^. More recently, taste receptors have also been investigated in several cancers including GI^24–30^, pancreatic^31,32^, breast^33^, thyroid^34^, acute myeloid leukemia^35^, and head and neck squamous cell carcinoma^36^. For example, single nucleotide polymorphisms (SNPs) in *TAS2R38* which lead to different haplotypes and taste perception have been shown to be broadly associated with overall cancer risk^37^ and risk of GI^24,25,28^ and colorectal^26^ malignancies specifically. In head and neck squamous cell carcinoma, T2Rs in tumor cells bind bitter bacterial metabolites to activate apoptosis, and patients with increased tumor expression of *TAS2R*s appear to have improved overall survival^36^. Bitter agonists trigger apoptosis and/or mitochondrial depolarization in other cancer cells as well^38–40^, including metastatic breast cancer^41^, prostate cancer^42^, and acute myeloid leukemia cells^35^.

We hypothesized that *TAS2R* and *TAS1R* genetic and expression alterations are common in various solid tumors and that these changes are associated with clinical outcomes. To gain further insight into taste receptor genetics in malignancy, we compared *TAS1R*s and *TAS2R*s in solid tumors to corresponding normal tissue using data derived from The Cancer Genome Atlas (TCGA)^43,44^ and the Genotype Tissue Expression Project (GTEx)^45^ databases^46^. We focused on differential expression (DE), mutations, and copy number variations for 20 types of solid tumors, including 45 subtypes, and investigated associations with gene expression and survival outcomes. Our findings identify several taste receptors that could serve as potential biomarkers for oncologic outcomes or therapeutic targets, warranting further exploration.

## Methods

### Data source

We utilized previoulsy published data on GPCRs from *Sriram *et al. (https://insellab.github.io/)^46^, derived from TCGA^43,44^ and the GTEx databases^45^, and data directly from TCGA obtained from cBioPortal (https://www.cbioportal.org/). Twenty types of solid tumors, including 45 histologic subtypes, were compared to corresponding normal tissue from the same anatomic sites. The GPCR genes examined included the 3 *TAS1R* and 24 of the 25 *TAS2R* genes (excluding *TAS2R45* which encodes an orphan receptor and did not have data available). This study was determined to be institutional review board exempt by the University of Pennsylvania.

### Differential expression analysis

Gene expression analysis between tumor and normal tissue was based on RNA-seq data derived from TCGA^43,44^ and the GTEx^45^. The methods used for data curation, DE analysis, and comparison of gene expression in tumors and normal tissue are previously described^46^. Gene expression data were available in transcripts per million (TPM), which is a normalization of gene abundance that corrects for effective gene length, and counts per million (CPM), which is the number of times a gene is encountered per million reads. TPM data were used for comparisons of different genes within a tumor dataset. CPM data were utilized for comparing gene expression between samples from separate datasets that may have been normalized differently.

### Mutation and copy number variation analysis

Mutation and copy number variation data sources and analysis are previously described^46^. Specifically, mutational data were obtained from the Baylor College of Medicine sequencing center and Broad Institute Automated Pipeline and included somatic, nonsilent mutations (gene-level) and somatic mutations (SNPs and small INDELs). Copy number estimates were obtained from TCGA and included homozygous/two-copy deletion, heterozygous/single-copy deletions, no change, low-level amplification, and high-level amplification.

### Statistical analysis

Analyses were conducted in R version 4.0.3 (2020-10-10). RNA data were processed by R package edgeR. Exact tests^47^ were used to estimate the fold-change (FC) in gene expression in tumors compared to normal tissue. A median tumor expression cutoff of > 0.001 TPM and log2 FC > log2(1.5) or < log2(1/1.5) were used. Gene changes with a false discovery rate (FDR) of < 0.05 were considered to be statistically significant. Normalized expression data for tumors and normal tissue were plotted as median expression and upper and lower quartiles.

Tumor samples were divided into high expression (above-median) and low expression (below-median) groups for survival comparisons. The difference in mean survival days was calculated for the high and low expression groups for different tumors. Kaplan–Meier survival analyses and Peto-Peto’s modified survival estimates were performed for comparisons of genes identified as having statistically significant differences in mean survival days for patients with clinical data available from cBioPortal (https://www.cbioportal.org/). Combined expression analyses were performed using a mean-normalized sum of expression of genes that individually predicted survival. A *p*-value < 0.05 was considered statistically significant for survival analyses. The prognostic effectiveness of gene expression data in the solid tumors was assessed using receiver operating characteristic (ROC) curves. ROC curve analysis was performed in R software using procedures from the ‘pROC’ package. The median CPM used in the Kaplan–Meier survival curves were used as the cutoffs for analysis.

### Ethics approval and consent to participate

This study was determined to be institutional review board exempt by the University of Pennsylvania.

### Consent for publication

Not applicable.

## Results

### *TAS1R* and *TAS2R* genes are differentially expressed in solid tumors

**Figure 1:**
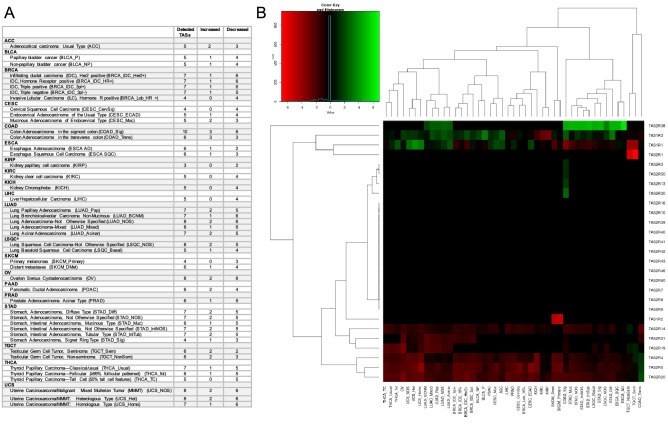
Multiple *TAS1R* and *TAS2R* genes are differentially expressed in solid tumors. To determine the differential expression of genes, RNA sequencing data for solid tumor tissues from The Cancer Genome Atlas (TCGA) were compared to corresponding normal tissues from the Genotype Tissue Expression Project (GTEx) database. (**A**) Table showing the solid tumors surveyed for *TAS1R/2R* differential expression analysis with TCGA cancer type, subtype/histology, and associated abbreviations. The number of *TAS1R/2Rs* with statistically significant changes in expression are listed for each tumor subtype compared to normal tissue with log2 fold change cutoff > log2(1.5) used for increased expression and < log2(1/1.5) for decreased expression (FDR < 0.05, median TPM > 0.001). (**B**) Heatmap demonstrating log2 fold-change of *TAS1R/2R* expression in 45 solid tumor subtypes compared to corresponding normal tissue with hierarchical clustering of differential expression. Increased expression shown in green and decreased expression in red. *DE* differential expression; *FDR* false discovery rate; *GTEx* Genotype Tissue Expression Project; *TPM* Transcripts Per Million; *TCGA* The Cancer Genome Atlas; ^a^LSQC is abbreviated as LUSC in some sources.

DE analysis of *TAS1R*s and *TAS2R*s was performed for 6,224 individual tumors from 45 solid tumor subtypes and compared to corresponding normal anatomic tissue. There were statistically significant differences in median tumor expression of multiple *TAS1R*s and *TAS2R*s across multiple cancers (Fig. 1). Most tumor subtypes had DE of at least one *TAS1R/2R* gene, and a numerically higher number of cancers studied had decreased gene expression rather than increased expression. *TAS2R4*, *TAS2R5*, *TAS2R14*, *TAS2R19*, *TAS2R20*, and *TAS2R31* were frequently expressed at lower levels in tumors compared to normal tissue; whereas, *TAS1R3* and *TAS2R38* were often expressed at higher levels. Other genes including *TAS1R1* showed increased and decreased expression depending on the tumor type.

Supplemental Fig. 1 shows the median expression in CPM for detectable *TAS1R/2R*s between normal and malignant tissues. In addition to the notable differences across malignancy types, these data also demonstrate the expression of taste receptors across normal tissues outside of the oral cavity and the variability that exists between normal tissues. *TAS1R*/*TAS2R* expression in TPM for each tumor type is shown in Supplemental Fig. 2. Within the same anatomic sites, there is variability in the expression levels of different *TAS1R*s and *TAS2R*s. Expression level variability follows similar patterns for cancer and normal tissue, with certain receptors consistently elevated or decreased relative to others. Supplemental Fig. 3 shows median expression in TPM and the log2 fold-change for tumors compared to normal tissue.

### Somatic mutations and copy number variation in *TAS1R* and *TAS2R* genes

**Figure 2:**
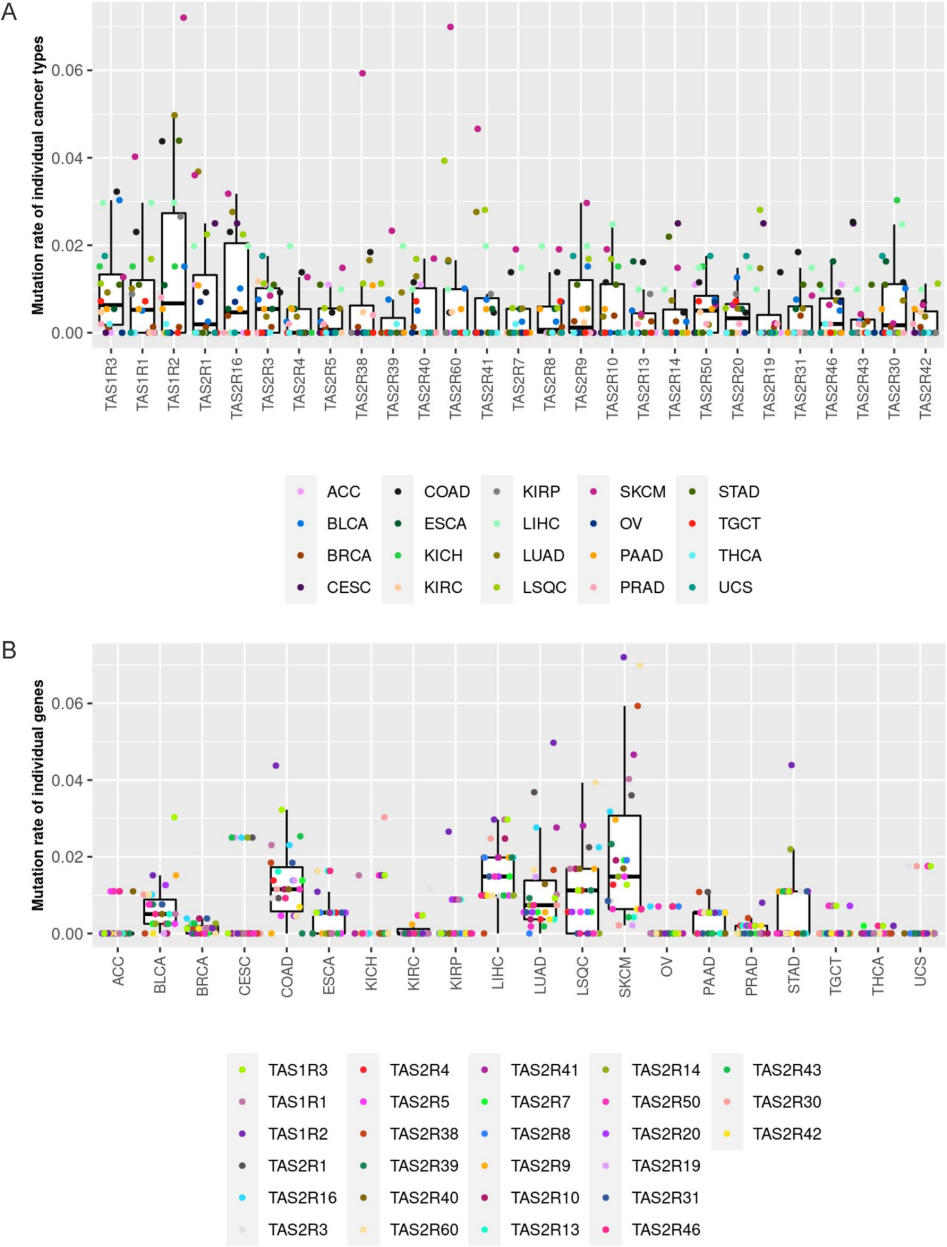
Somatic mutations in *TAS1R* and *TAS2R* genes in solid tumors. Analysis of nonsilent mutations in *TAS1R* and *TAS2R* genes for different TCGA solid tumor types (n = 5103 for 20 tumor types with data available), stratified by (**A**) gene and (**B**) cancer type. Dots represent the mutation rates in each tumor type or *TAS1R*/*2R* gene. *ACC* Adrenocortical Cancer; *BLCA* bladder cancer; *BRCA* breast cancer; *CESC* Cervical Cancer; *COAD* colon adenocarcinoma; *ESCA* esophageal cancer; *KICH* Kidney Chromophobe; *KIRC* kidney clear cell carcinoma; *KIRP* kidney papillary cell carcinoma; *LIHC* liver hepatocellular carcinoma; *LSQC* lung squamous cell carcinoma; *LUAD* lung adenocarcinoma; *OV* ovarian cancer; *PAAD* pancreatic ductal adenocarcinoma; *PRAD* prostate adenocarcinoma; *SKCM* skin cutaneous melanoma; *TCGA* The Cancer Genome Atlas; *TGCT* testicular germ cell tumor, *THCA* thyroid cancer; *UCS* Uterine Carcinosarcoma.

Analysis of *TAS1R* and *TAS2R* mutations performed for all solid tumors with data available (n = 5103) showed that nonsilent mutations occur in these genes (Fig. 2). Across all tumor types, the median percentage of tumors with nonsilent mutations in *TAS1R*s, including *TAS1R1*, *TAS1R2*, *TAS1R3*, was slightly less than 1%. Similarly, the *TAS2R*s had a median percentage of tumors with nonsilent mutations less than 1% (Fig. 2A).

The proportion of tumors with nonsilent *TAS1R* and *TAS2R* mutations varied for each cancer type with skin melanoma having the highest rate of mutations overall (Fig. 2A, B). Specifically, nonsilent mutation rates in skin melanoma were approximately 7% for *TAS1R2* and *TAS2R60*, 6% for *TAS2R38*, and 4.5% for *TAS2R41*. In liver hepatocellular carcinoma, the 3 *TAS1R*s were each mutated in about 3% of individuals. *TAS1R2* was mutated in roughly 5% of lung, colon, and stomach adenocarcinomas and *TAS2R60* was mutated in ~ 4% of lung squamous cell carcinomas.

**Figure 3:**
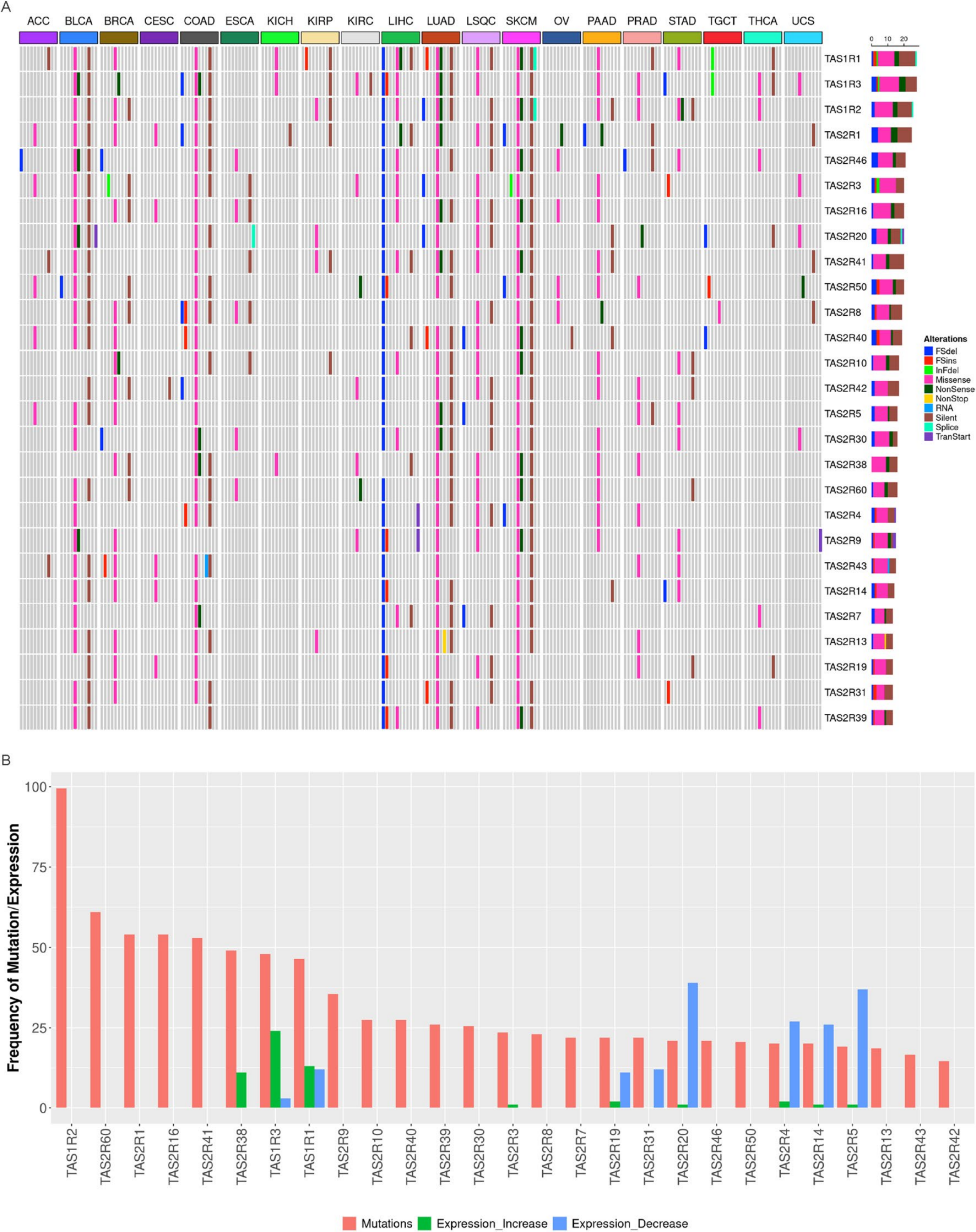
*TAS1R* and *TAS2R* mutation types in solid tumors and correlation of gene mutations with expression. (**A**) Mutation landscape showing the different mutation types for *TAS1R* and *TAS2R* genes for different TCGA solid tumor types (n = 5103 for 20 tumor types with data available). (**B**) Graph demonstrating the number of tumors with nonsilent taste receptor mutations and the frequency of increased or decreased expression for the same receptor. *ACC* Adrenocortical Cancer; *BLCA* bladder cancer; *BRCA* breast cancer; *CESC* Cervical Cancer; *COAD* colon adenocarcinoma; *ESCA* esophageal cancer; *KICH* Kidney Chromophobe; *KIRC* kidney clear cell carcinoma; *KIRP* kidney papillary cell carcinoma; *LIHC* liver hepatocellular carcinoma; *LSQC* lung squamous cell carcinoma; *LUAD* lung adenocarcinoma; *OV* ovarian cancer; *PAAD* pancreatic ductal adenocarcinoma; *PRAD* prostate adenocarcinoma; *SKCM* skin cutaneous melanoma; *TCGA* The Cancer Genome Atlas; *TGCT* testicular germ cell tumor, *THCA* thyroid cancer; *UCS* Uterine Carcinosarcoma.

The types of somatic mutations in taste receptors for each solid tumor type are shown in Fig. 3A and Supplemental Fig. 4. For most tumors, missense mutations were the most common types of somatic mutations followed by silent mutations. Liver hepatocellular carcinoma, which had prevalent mutations in the 3 *TAS1R* genes located on chromosome 1, was unique for its relatively high proportion of frame shift deletions relative to the other malignancies. Figure 3B demonstrates the number of tumors with nonsilent taste receptor mutations and the frequency of increased or decreased expression for the same receptor. The frequency of increased or decreased taste receptor expression in tumors does not clearly correlate with the frequency of mutations.

**Figure 4:**
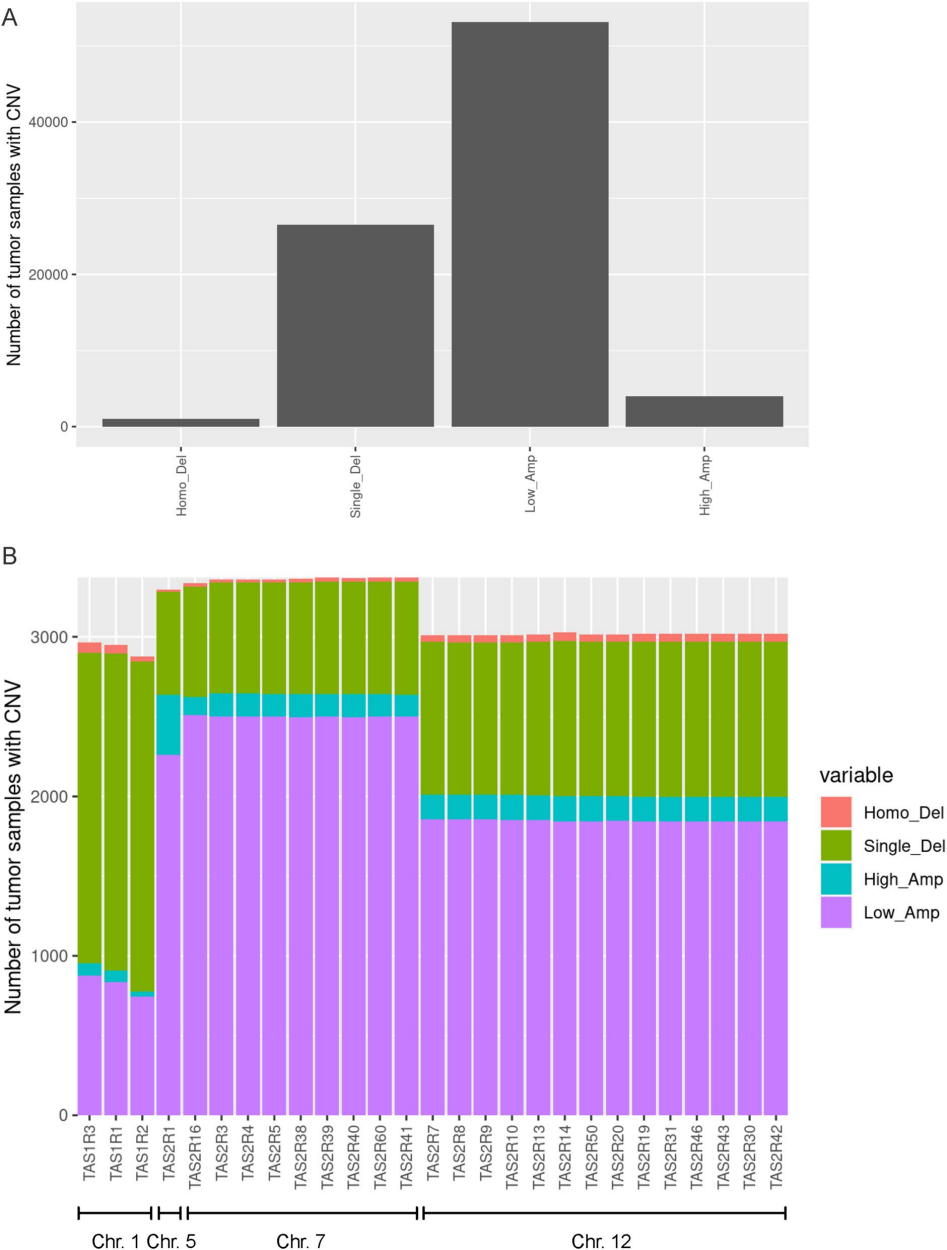
Copy number variation (CNV) in *TAS1R* and *TAS2R* genes in solid tumors. (**A**–**B**) Graphical representations of CNV in *TAS1R* and *TAS2R* genes for 20 tumor types (n = 7545) including the total number of homozygous deletions, single-copy deletions, low-level amplifications, and high-level amplifications. Data is shown for (**A**) all *TAS1R* and *TAS2R* genes and for (**B**) each gene organized by chromosome location. *CNV* copy number variation; *Chr.* chromosome.

CNV analysis was performed for *TAS1R* and *TAS2R* genes in 7,545 individual samples from 20 tumor types (Fig. 4). Overall, low-amplification was the most common type of CNV followed by single copy deletions (Fig. 4A). However, for *TAS1R*s, single copy deletions were more common than low-amplifications (Fig. 4B). The CNV pattern appeared to cluster based on the chromosome location of the different taste receptor genes. The mutation and CNV analyses indicate that there are alterations in taste receptor genes that occur in malignancy.

### *TAS1R* and *TAS2R* expression is associated with survival

**Figure 5:**
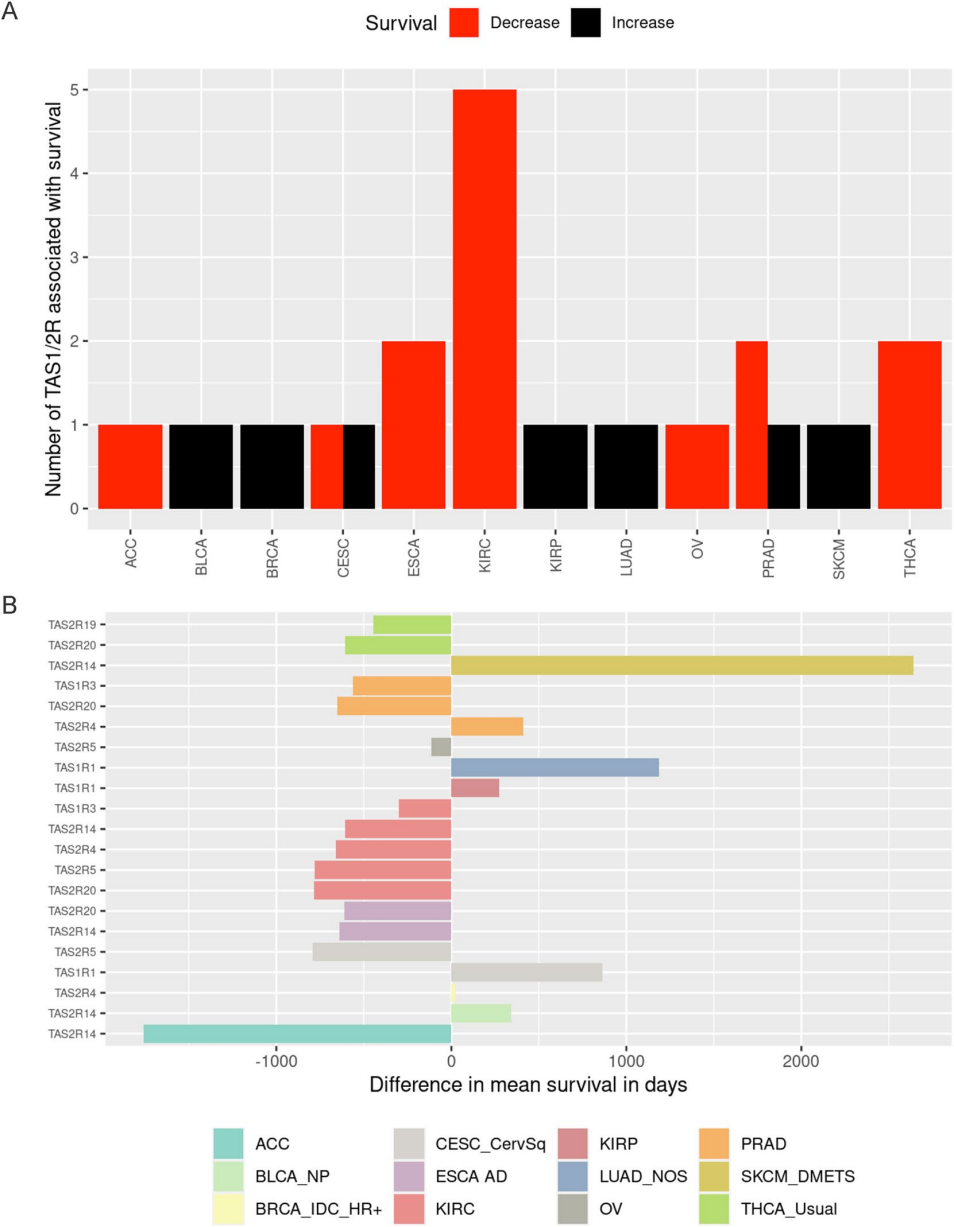
*TAS1R* and *TAS2R* expression is associated with survival in solid tumors. Tumor samples were divided into high and low expression groups and the difference in mean survival days was calculated for these groups for each *TAS1R*/*TAS2R* gene and tumor. (**A**) Bar graph demonstrating the number of genes significantly associated with increased (black) or decreased (red) survival in solid tumors (*p* < 0.05). Non-significant associations are not shown. Negative survival difference indicates that higher gene expression corresponds to shorter survival times while positive survival difference indicates that higher gene expression corresponds to longer survival times. (**B**) Difference in mean survival in days between above-median and below-median expression groups for each *TAS1R* and *TAS2R* genes for tumors that reached significance in (**A**). Negative values imply an adverse impact on survival where patients with higher median expression survived shorter than those with lower median expression and vice versa.​ *ACC* adrenocortical cancer; *BLCA_NP* non-papillary bladder cancer; *BRCA_IDC_HR +* hormone receptor positive infiltrating ductal carcinoma; *CESC_CervSq* cervical esophageal squamous cell carcinoma; *CPM* counts per million; *ESCA_AD* esophageal adenocarcinoma; *KIRC* kidney clear cell carcinoma; *KIRP* kidney papillary cell carcinoma; *LUAD_NOS* lung adenocarcinoma not otherwise specified; *OV* ovarian cancer; *PRAD* prostate adenocarcinoma; *SKCM_DistantMets* distantly metastatic skin melanoma; *THCA_usual* thyroid cancer usual type.

After demonstrating that taste receptor genetic and expression alterations occur in various solid tumors, we sought to determine if these genes served as prognostic markers for clinical outcomes. By comparing tumors with high and low expression of specific taste receptor genes (defined as above or below the median expression, respectively), we found that expression of at least one *TAS1R* or *TAS2R* was statistically significantly associated with mean survival differences in 12 subtypes of solid tumors (Fig. 5A). When examining statistically significant associations, higher gene expression was most commonly associated with shorter mean survival times (n = 14) compared to longer survival times (n = 7). Increased gene expression in the following taste receptor genes had negative survival associations (i.e. higher expression correlating with worse survival) in at least one tumor histology: *TAS1R3*, *TAS2R14*, *TAS2R19*, *TAS2R20*, *TAS2R4*, and *TAS2R5*. Positive survival associations (i.e. higher expression correlating with improved survival) were identified for *TAS1R1*, *TAS2R14*, and *TAS2R4* in at least one malignancy.

The degree of survival impact was determined by calculating the difference in mean survival days between above- and below-median expression groups for *TAS1R* and *TAS2R* genes in different tumors as shown in Fig. 5B and Supplemental Table 1. The most dramatic positive survival association was present in melanoma distant metastases for *TAS2R14* with the high-expression group surviving a mean 2641 days (~ 7.2 years) longer than the low-expression group (*p* = 0.0127). Adrenocortical carcinoma had the largest negative survival association for *TAS2R14* with mean survival difference of −1757 days (*p* = 0.0007). *TAS1R1* had profound positive survival associations in lung adenocarcinoma–not otherwise specified (mean difference 1185 days, *p* = 0.0191) and cervical squamous cell carcinoma (mean difference 862 days, *p* = 0.0098).

**Figure 6:**
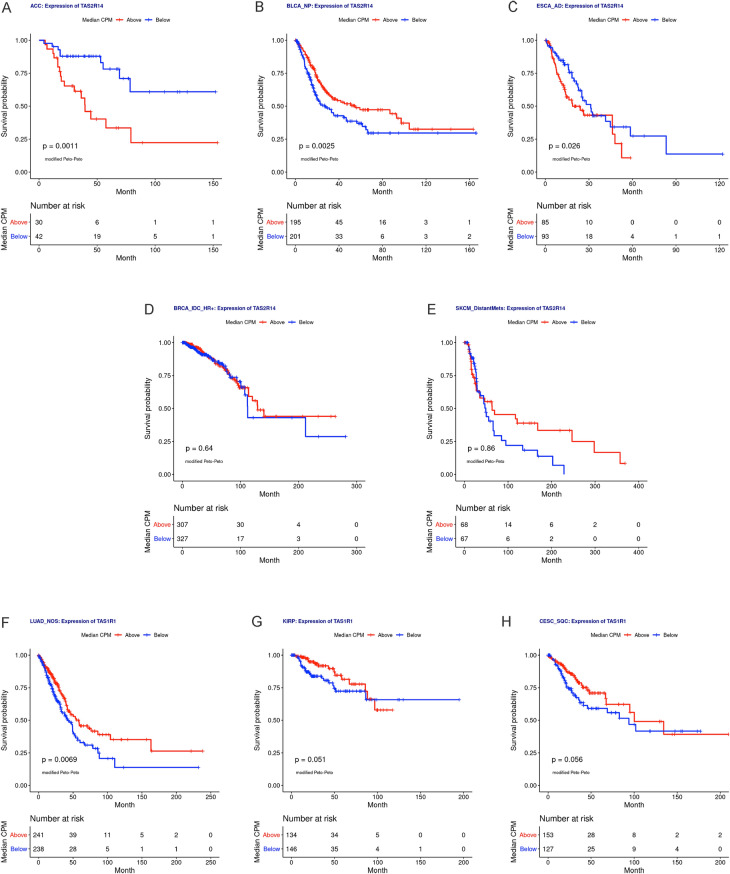
Bitter taste receptor *TAS2R14* and umami receptor *TAS1R1* expression are associated with survival in select solid tumors. (**A**–**E**) Kaplan–Meier curves for high and low expression groups for *TAS2R14* in (**A**) adrenocortical cancer (ACC), (**B**) non-papillary bladder cancer (BLCA_NP), (**C**) esophageal adenocarcinoma (ESCA_AD), (**D**) hormone receptor positive infiltrating ductal breast carcinoma (BRCA_IDC_HR +), and (**E**) distantly metastatic skin melanoma (SKCM_DistantMets). Peto-Peto’s modified survival estimate was significant for *TAS2R14* expression in ACC, BLCA_NP, ESCA_AD, and kidney clear cell carcinoma (KIRC; shown in Supplemental Fig. 5). (**F**–**H**) Kaplan–Meier survival curves for high and low expression groups for *TAS1R1* in (**F**) lung adenocarcinoma (LUAD_NOS), (**G**) kidney papillary cell carcinoma (KIRP), and (H) cervical esophageal squamous cell carcinoma (CESC_SQC). Peto-Peto’s modified survival estimate was statistically significant for *TAS1R1* expression in LUAD_NOS. *ACC* adrenocortical cancer; *BLCA_NP* non-papillary bladder cancer; *BRCA_IDC_HR + * hormone receptor positive infiltrating ductal carcinoma; *CESC_SQC* cervical esophageal squamous cell carcinoma; *CPM* counts per million; *ESCA_AD* esophageal adenocarcinoma; *KIRC* kidney clear cell carcinoma; *KIRP* kidney papillary cell carcinoma; *LUAD_NOS* lung adenocarcinoma; *SKCM_DistantMets* distantly metastatic skin melanoma.

We performed Kaplan–Meier survival analyses for any taste receptor genes identified as having statistically significant mean survival differences between high and low expression groups and highlighted the most frequently associated bitter taste receptor*, TAS2R14* and umami taste receptor*, TAS1R1* (Fig. 6). Kaplan–Meier survival analyses for high- and low-expression groups of *TAS2R14* were statistically significantly associated with survival for adrenocortical cancer (*p* = 0.0011), non-papillary bladder cancer (p = 0.0025), and esophageal adenocarcinoma (*p* = 0.026; Fig. 6A–E). Kaplan–Meier survival analyses for high- and low-expression groups of *TAS1R1* was statistically significant for lung adenocarcinoma (*p* = 0.0069) but not for kidney papillary cell carcinoma (*p* = 0.051) or cervical esophageal squamous cell carcinoma (*p* = 0.056, Fig. 6F–H).

Kaplan–Meier analysis of kidney clear cell carcinoma showed significant survival associations for high and low expression groups for *TAS1R3* (*p* = 0.0062), *TAS2R4* (*p* = 0.015), *TAS2R5* (*p* = 0.0029), *TAS2R14* (*p* < 0.001), and *TAS2R20* (*p* < 0.001) and the mean-normalized sum of expression of these genes that individually predicted survival (*p* < 0.001, Supplemental Fig. 5). Additional Kaplan–Meier survival curves for expression of *TAS2Rs*, *TAS1Rs*, and combined taste receptor gene groups are shown in Supplemental Fig. 6. Notably, statistically significant survival associations were identified for *TAS2R5* in cervical squamous cell carcinoma (*p* < 0.001), *TAS2R20* in esophageal adenocarcinoma (*p* = 0.044), and *TAS2R4, TAS2R20,* and *TAS1R3* in prostate adenocarcinoma (*p* = 0.03, *p* = 0.033, and *p* = 0.023, respectively).

### Prognostic roles of *TAS2R14* and *TAS1R1* for survival

**Figure 7:**
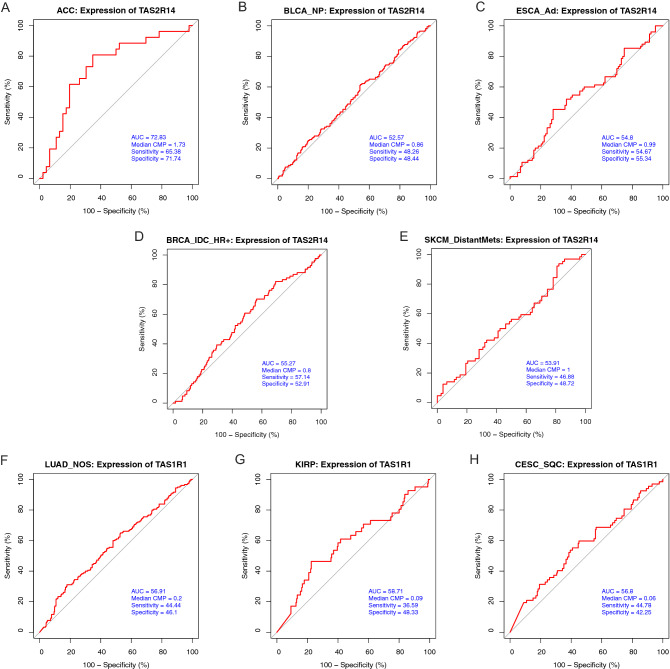
Prognostic roles of *TAS2R14* and *TAS1R1* for survival in solid tumors. Gene expression data from TCGA was used to generate receiver operating characteristic (ROC) curves using the median counts per million (CPM) cutoffs used in the Kaplan-Meier survival curves from Fig. 6. ROC curves are shown for *TAS2R14* in (**A**) adrenocortical cancer (ACC), (**B**) non-papillary bladder cancer (BLCA_NP), (**C**) esophageal adenocarcinoma (ESCA_AD), (**D**) hormone receptor positive infiltrating ductal breast carcinoma (BRCA_IDC_HR +), and (**E**) distantly metastatic skin melanoma (SKCM_DistantMets). ROC curves were also generated for *TAS1R1* in (**F**) lung adenocarcinoma (LUAD_NOS), (**G**) kidney papillary cell carcinoma (KIRP), and (**H**) cervical esophageal squamous cell carcinoma (CESC_SQC). The red line represents the sensitivity curve, and the blue line represents random chance. The X axis shows the false positive rate, presented as ‘100–Specificity (%)’. The Y axis indicates the true positive rate, shown as ‘Sensitivity (%)’. The area under the receiver operating characteristics was highest for *TAS2R14* in adrenocortical cancer (sensitivity 65.4% and specificity 71.7%). *ACC* adrenocortical cancer; *BLCA_NP* non-papillary bladder cancer; *BRCA_IDC_HR +* hormone receptor positive infiltrating ductal carcinoma; *CESC_SQC* cervical esophageal squamous cell carcinoma; *CPM* counts per million; *ESCA_AD* esophageal adenocarcinoma; *KIRC* kidney clear cell carcinoma; *KIRP* kidney papillary cell carcinoma; *LUAD_NOS* lung adenocarcinoma; *ROC* receiver operating characteristic; *SKCM_DistantMets* distantly metastatic skin melanoma.

The median CPM expression cutoffs used in the Kaplan–Meier survival curves from Fig. 6 were used to generate ROC curves to assess the prognostic roles of *TAS2R14* and *TAS1R1* in certain solid tumors (Fig. 7). The ROC curves demonstrated that these genes had different specificity and sensitivity for predicting survival of solid tumor patients. The area under the receiver operating characteristics was highest for *TAS2R14* in adrenocortical cancer with associated sensitivity 65.4% and specificity 71.7%. The predictive role of the other evaluated genes was relatively poor.

## Discussion

Despite being best known for their role in taste sensing^1^, T2Rs and T1Rs have been identified in various extra-oral tissues where they serve diverse chemosensory roles^17–22,48^. Emerging data on the role of taste receptors in malignancy led us to explore the genetic and expression alterations for solid tumors and implications on survival. T2Rs have been studied in some cancers^24–29,31–36^, but we are the first to show their expression across numerous types of solid cancer tissues using data from TCGA. Our demonstration of *TAS1R* expression in solid tumors is also highly novel, as there are no prior reports in the literature to our knowledge.

Using the GTEx data, we verified previous studies showing taste receptor expression in normal, extra-oral human tissues such as the lung^20^, GI tract^21,22^, and skin^49,50^. *TAS2R14* was detected at relatively high levels in many normal tissues in our study which is consistent with prior expression analyses including work in human embryonic kidney (HEK) 293 cell lines and human epidermal keritoncytes where T2R14 is functional and serves as a chemosensory receptor^51,52^. Our data on normal tissue expression provides a key resource for exploring potential taste receptor pathways in physiologically normal tissue.

The DE comparison of *TAS1R/2R*s between normal and cancerous tissues showed several expression alterations that varied between cancer types. Compared to their corresponding normal tissue, most solid tumors differentially expressed one or more taste receptor gene and there was a trend toward decreased expression of *TAS2R*s across many of the malignancies. Bitter agonists, which bind to functional T2Rs, have been shown to activate apoptosis in prostate cancer^42^, metastatic breast cancer^41^, acute myeloid leukemia cells^35^, pancreatic cancer^32^, and head and neck squamous cell carcinoma^36^. Given the potential role of some T2Rs in regulating apoptosis, it is possible that decreased expression of *TAS2Rs* contributes to unregulated proliferation in malignancy or serves an integral role in oncogenesis, potentially explaining the general trend towards decreased expression in cancer compared to normal tissue. Importantly, our DE analysis compared the median expression for tumor versus normal tissue and did not specifically explore the distribution of *TAS1R/2R* expression of individual samples for a given anatomic site/cancer which may mask or dilute variability in expression that exists between samples/individuals.

Using the same logic that some T2Rs may regulate apoptosis and impact growth regulation, we predicted that taste receptor expression levels may be a prognostic feature for stratifying outcomes in solid tumors. We did in fact find positive survival differences for higher expression of some taste receptor genes in certain malignancies; however, more taste receptors were associated with a negative survival difference (i.e. higher gene expression corresponded to shorter survival times). These findings may be related to the diverse array of functions and ligands for the T1Rs and T2Rs and potential synergistic or antagonistic effects of these receptors and ligands in different tumor types. Alternatively, changes in taste receptor expression may be related to other genetic changes in cancer cells that have more pronounced impacts on survival.

Regardless of receptor function, *TAS1R* and *TAS2R* gene expression had significant survival associations in numerous subtypes of solid tumors, suggesting that they could potentially be refined to serve as biomarkers for disease prognosis or selection of treatment intensity in some cancers. Changes in expression for *TAS1R1*, *TAS1R3*, *TAS2R4, TAS2R5, TAS2R14*, *TAS2R19*, and *TAS2R20* had survival associations in at least one tumor histology. The most notable survival associations were seen for expression of *TAS2R14* in adrenocortical carcinoma, esophageal adenocarcinoma, and non-papillary bladder cancer which were significant based on analysis of mean survival difference and Kaplan–Meier analysis. Similarly, increased *TAS1R1* expression was associated with improved survival in lung adenocarcinoma in both analysis methods. The efficacy of median *TAS2R14* and *TAS1R1* expression in predicting survival was assessed (with *TAS2R14* in adrenocortical carcinoma performing best), but additional biomarker development is necessary to determine if combinations of various *TAS1R/TAS2R* expression levels and additional clinical variables may allow for improved sensitivity and specificity. Interestingly, kidney clear cell carcinoma showed significant negative survival associations for 5 genes (*TAS1R1*, *TAS2R4*, *TAS2R5*, *TAS2R14*, and *TAS2R20*) and a combined group based on expression of these 5 genes, which suggests that this tumor type may be particularly well suited for a genetic risk screening tool based on *TAS2R*s. The candidate prognostic genes identified in our study will need to be further evaluated and validated to fully understand their clinical utility.

Because specific genes, such as *TAS2R4* and *TAS2R14*, had opposing survival associations in different malignancies, it is possible that their functions in certain cancers vary and extend beyond apoptotic signaling pathways previously described. The variability in T2R function has been evidenced in basic science work where common bitter ligands that have anti-proliferative effects in some cancers^32,35,36,41,42^ instead have pro-tumor actions in other malignancies such as submandibular gland cancer cells^53^. Adding to the potential multifaceted impact of taste receptors, certain T2Rs are activated by bacterial metabolites, such as T2R38 activation by *Pseudomonas aeruginosa* N-3-oxo-dodecanoyl-L-homoserine lactone in pancreatic cancer^32^, suggesting that tumor-microbiome crosstalk and the specific microenvironment may impact cancer through these receptors. From a therapeutic perspective, taste receptors with positive impacts on survival could potentially be targetted with T1R/T2R-activating drugs delivered topically to a tumor. Alternatively, receptors with pro-tumor effects could be candidates for immunomodulators or biologics aimed at neutralizing their function.

We showed that nonsilent mutations occurred in *TAS1R* and *TAS2R* genes and occurred at different rates for different cancer types. Some of the highest rates of mutations occurred in skin melanoma, liver hepatocellular carcinoma, lung adenocarcinoma, colon adenocarcinoma, stomach adenocarcinoma, and lung squamous cell carcinoma. Importantly, *TAS2R* genes have a high density of polymorphisms, including amino acid substitutions, compared with other gene families^55^. Polymorphisms in taste receptor genes that regulate their function in terms of taste have also been linked to alterations in susceptibilty to infection and inflammatory disease due to their role in immunity^18^. We propose that these same polymorphisms may have even further reaching clinical roles by regulating T2R function within tumor cells. These pre-existing polymorphisms may have equal or more profound impacts in cancer than the cancer-derived mutations in these genes. While the clinical impact of taste receptor mutations and polymorphisms was not specifically analyzed in the current study, this should be the topic of future work.

Importantly, the complex taste receptor genetics underlying individual taste preferences (e.g., for bitter coffee, green leafy vegetables, hoppy beer)^11–16^ may also underlie outcomes in cancer through their impact on diet and consumption of certain foods and beverages. For example, one prior study showed that *TAS1R* genetic variations (determined by blood sample genotype) were associated with dietary fruit consumption and cigarette use and possibly gastric cancer risk^30^. Further work is required to fully separate out tumor cell-dependent versus diet-dependent effects of taste receptor polymorphisms.

In summary, this study was the first to characterize taste receptor DNA mutations, structural variation, differential expression, and survival associations across numerous solid tumors. Our finding that expression levels of specific taste receptor genes predicted survival suggests that these genes could serve as biomarkers for clinical outcomes or targets in certain malignancies. There are several limitations of our study which can serve as opportunities for additional analyses. These limitations include the need for investigation of taste receptor function in malignancy, analysis of survival associations based on genetic mutations and polymorphisms, and incorporation of a wider range of clinical variables and outcome measures. Furthermore, in vitro and in vivo studies on the functional role of taste receptors and their genes are warranted to reveal potential associations with cancer progression and behavior which cannot be fully determined through in silico analyses. Regardless, our work will serve as a launching point for exploration of this novel class of GPCRs in malignancy which may improve disease stratification and treatment.

## Supplementary Information

Supplementary Information.

## Data Availability

All relevant data are within the paper and its Supporting information files, with additional information available at the open-access websites https://insellab.github.io/ and https://www.cbioportal.org/.
