# Supplementary material for: Impact of sweet, umami, and bitter taste receptor (TAS1R and TAS2R) genomic and expression alterations in solid tumors on survival
Source: Sci Rep. 2022 May 27;12:8937. doi: 10.1038/s41598-022-12788-z (PMC9142493; doi:10.1038/s41598-022-12788-z)
Supplement: Supplementary file 1 — Supplementary Information. [file 41598_2022_12788_MOESM1_ESM.docx]

**Supplementary Material**

Carey, et al. Impact of sweet, umami, and bitter taste receptor (*TAS1R* and *TAS2R*) genomic and expression alterations in solid tumors on survival.


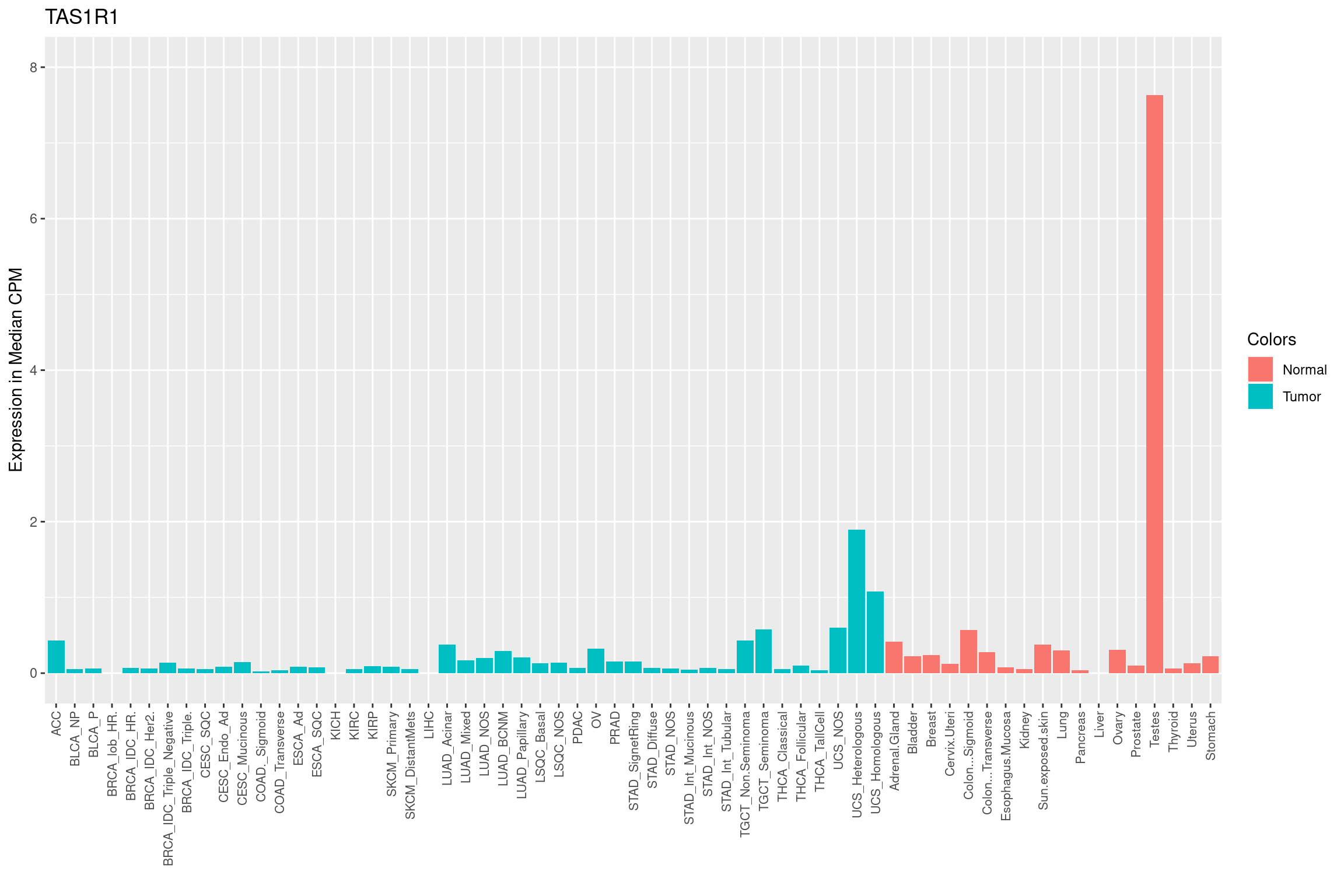

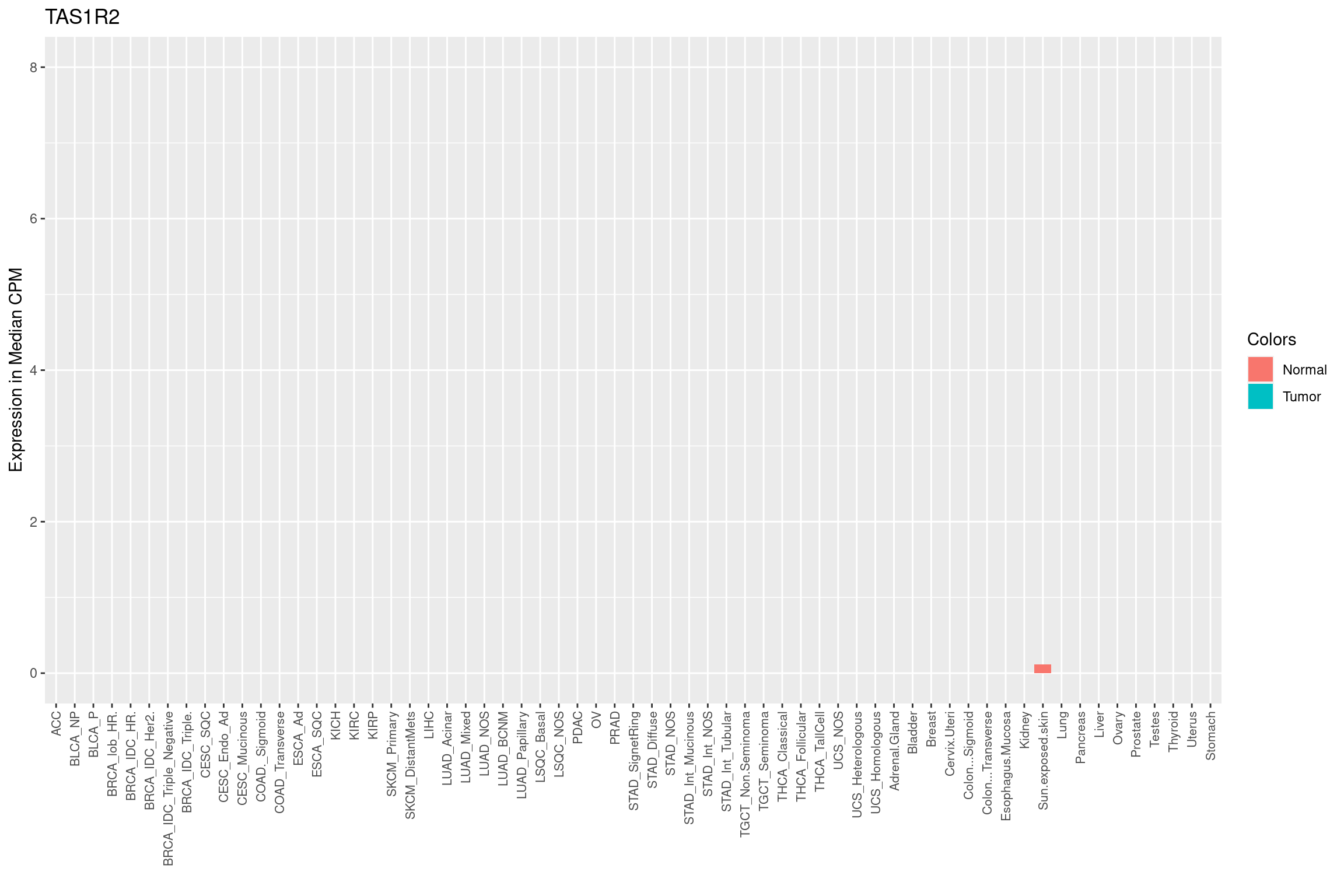

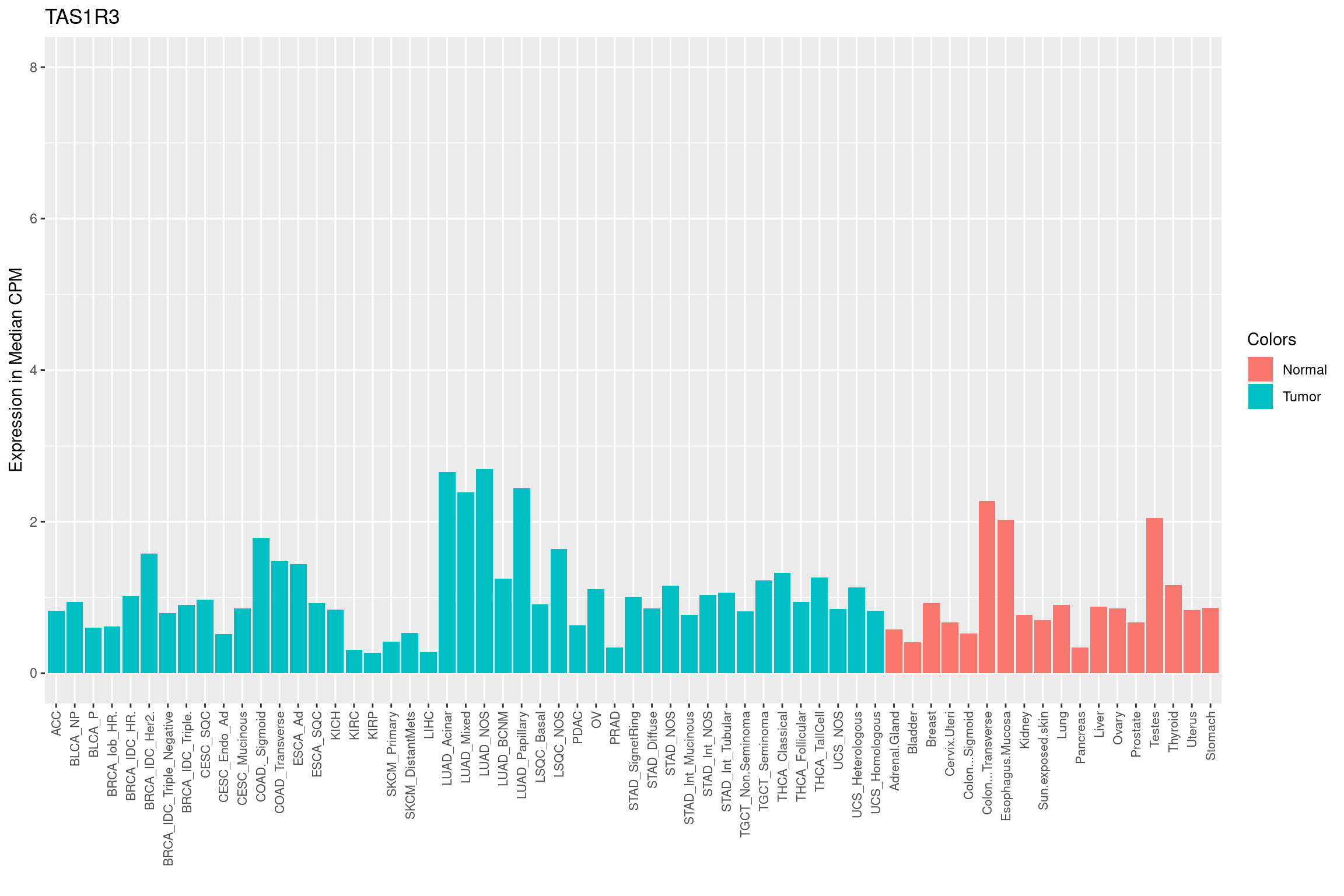

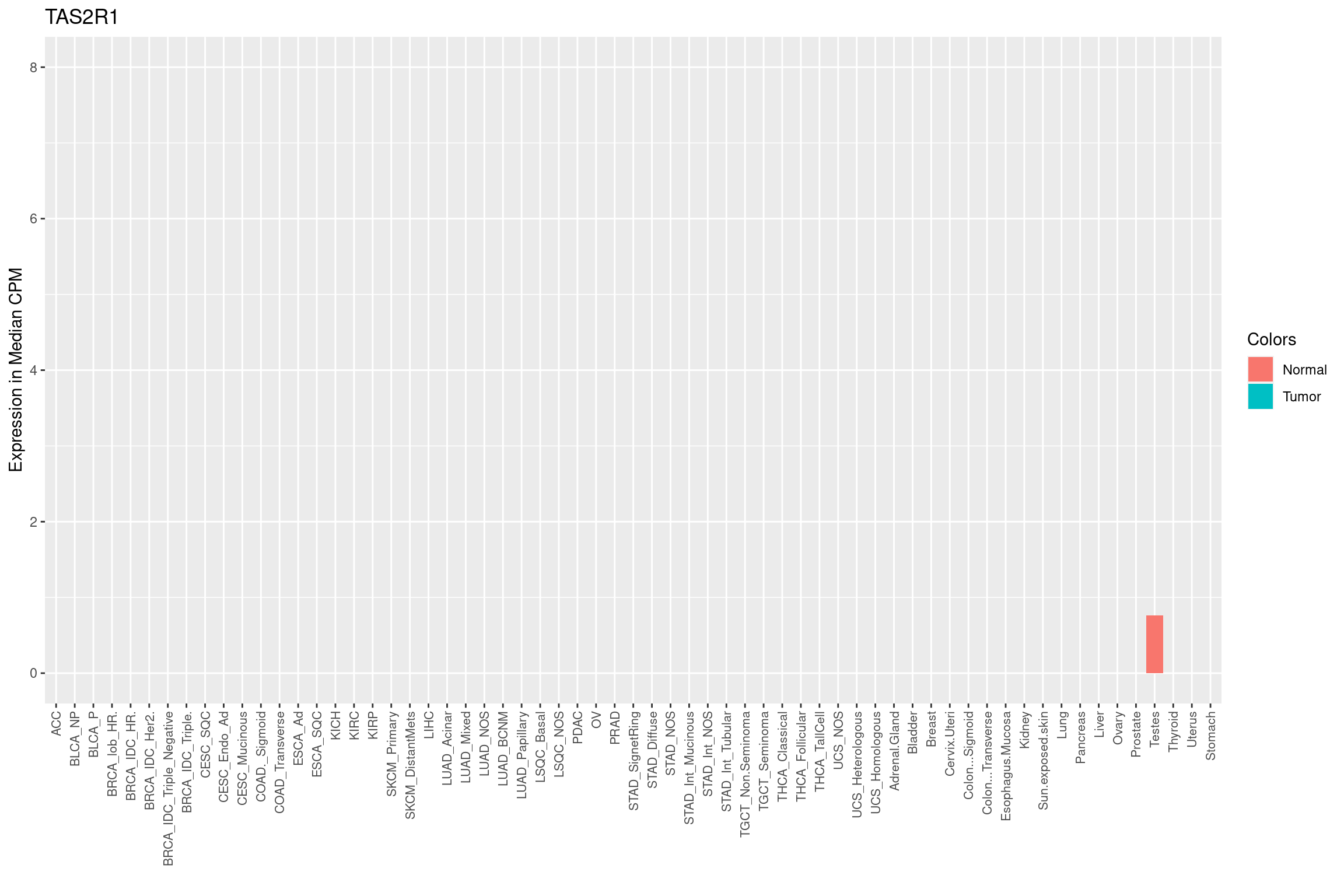

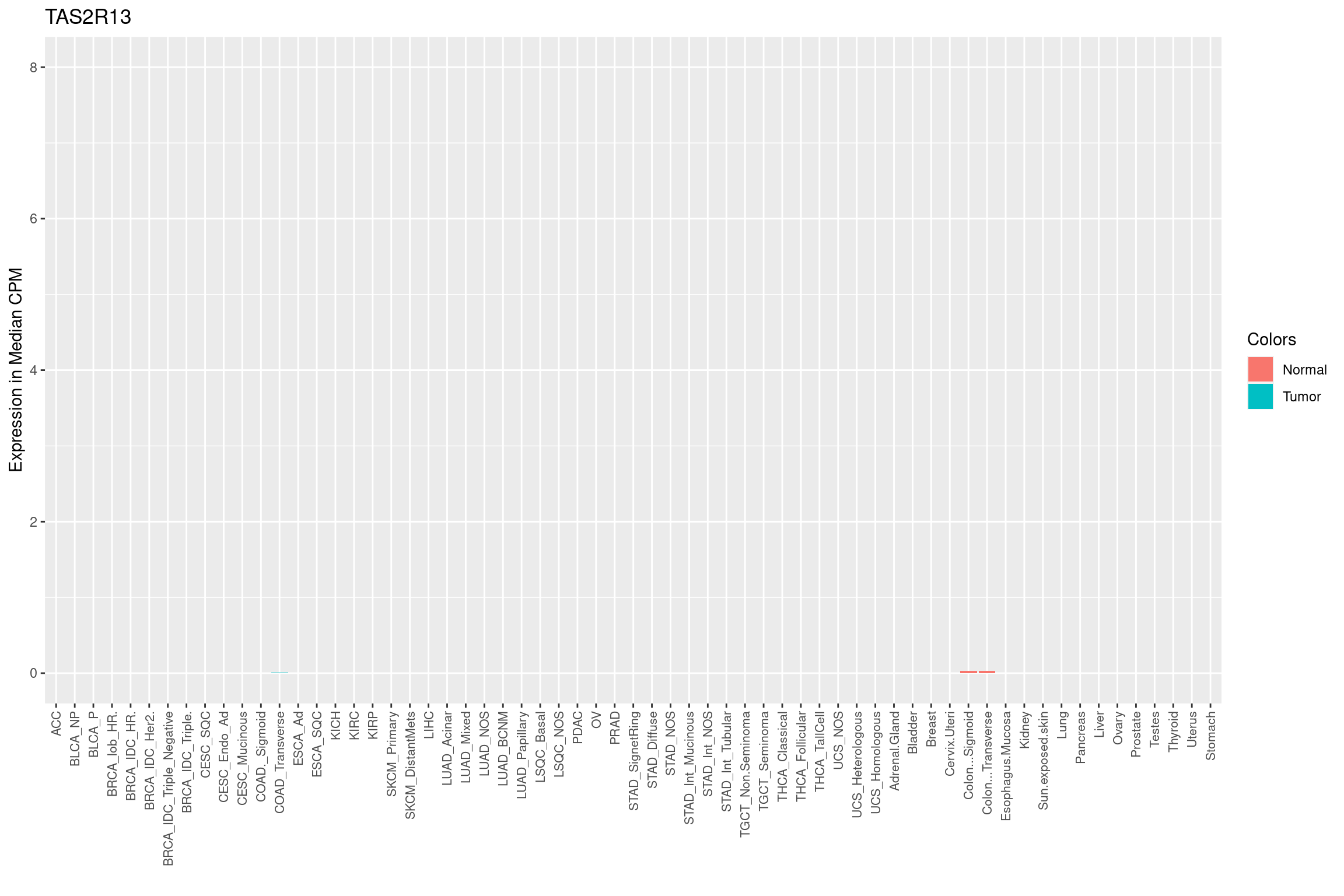

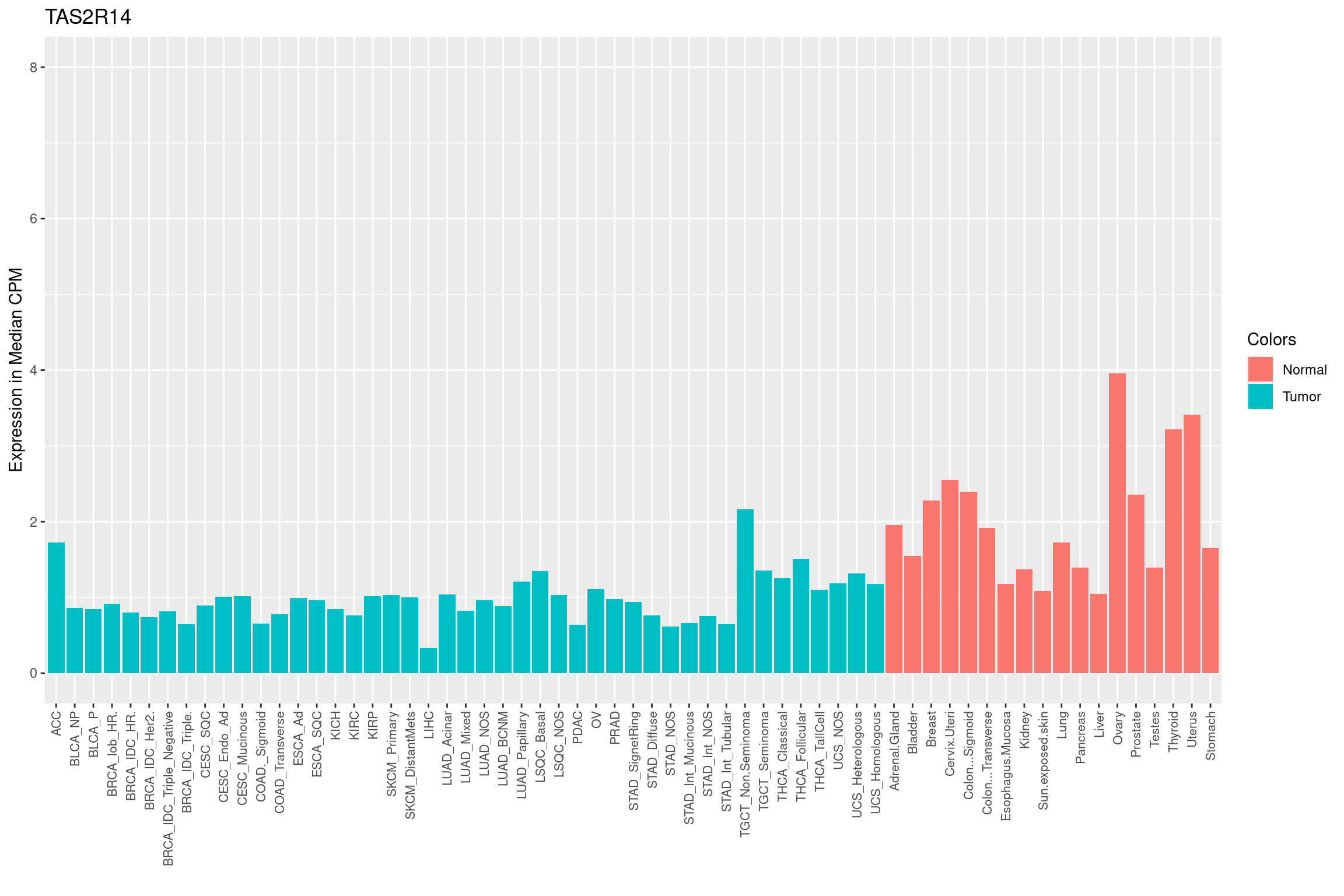

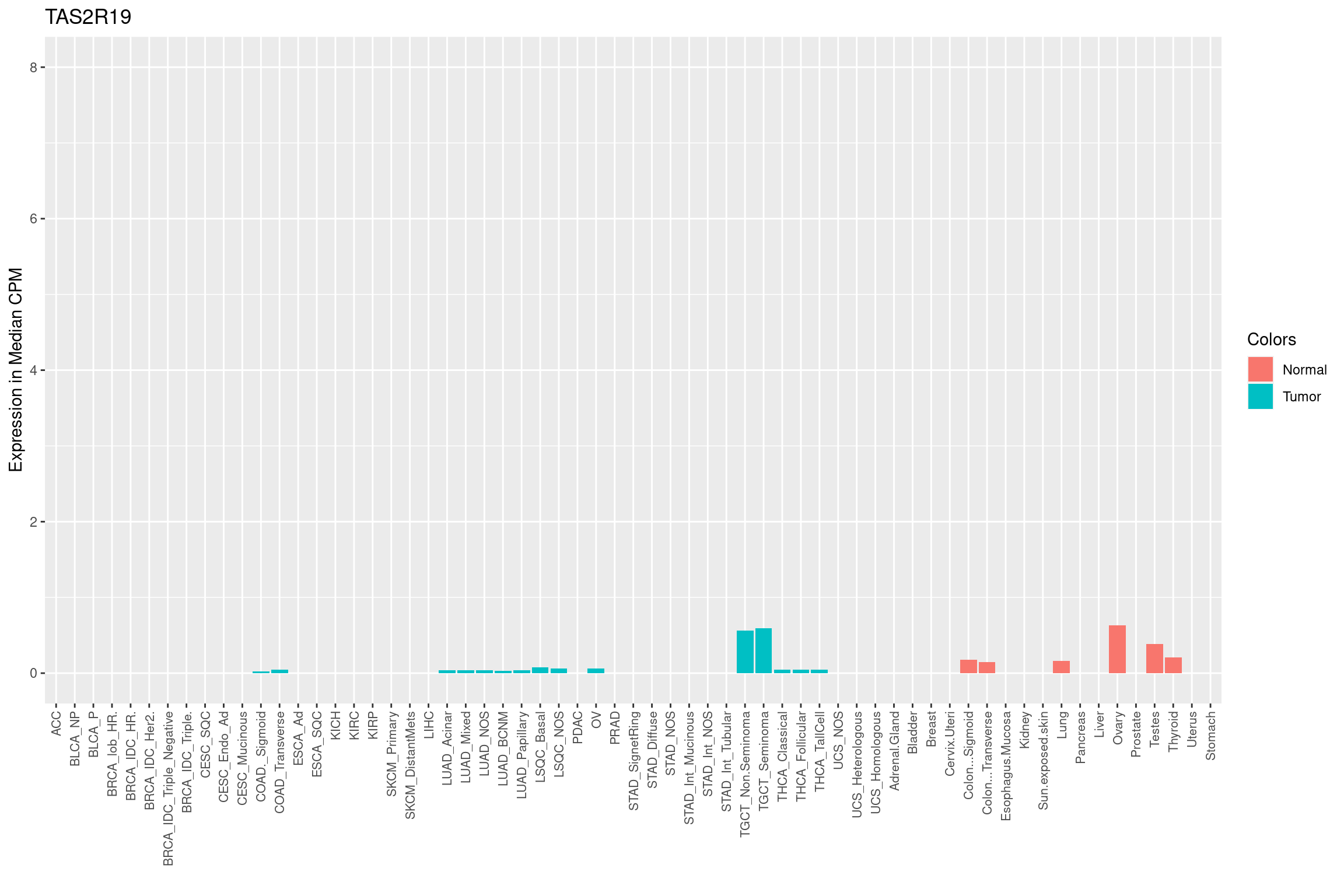

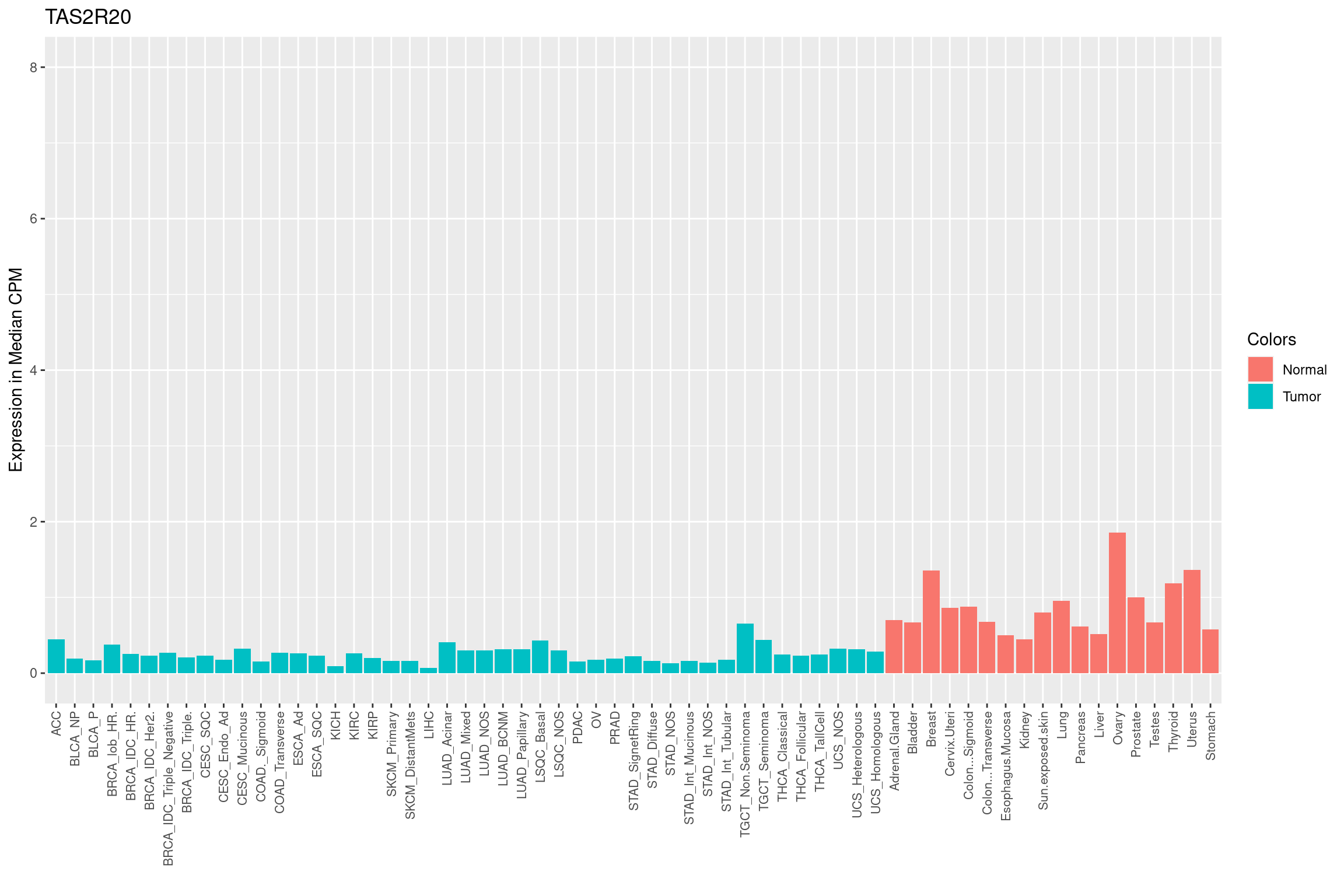

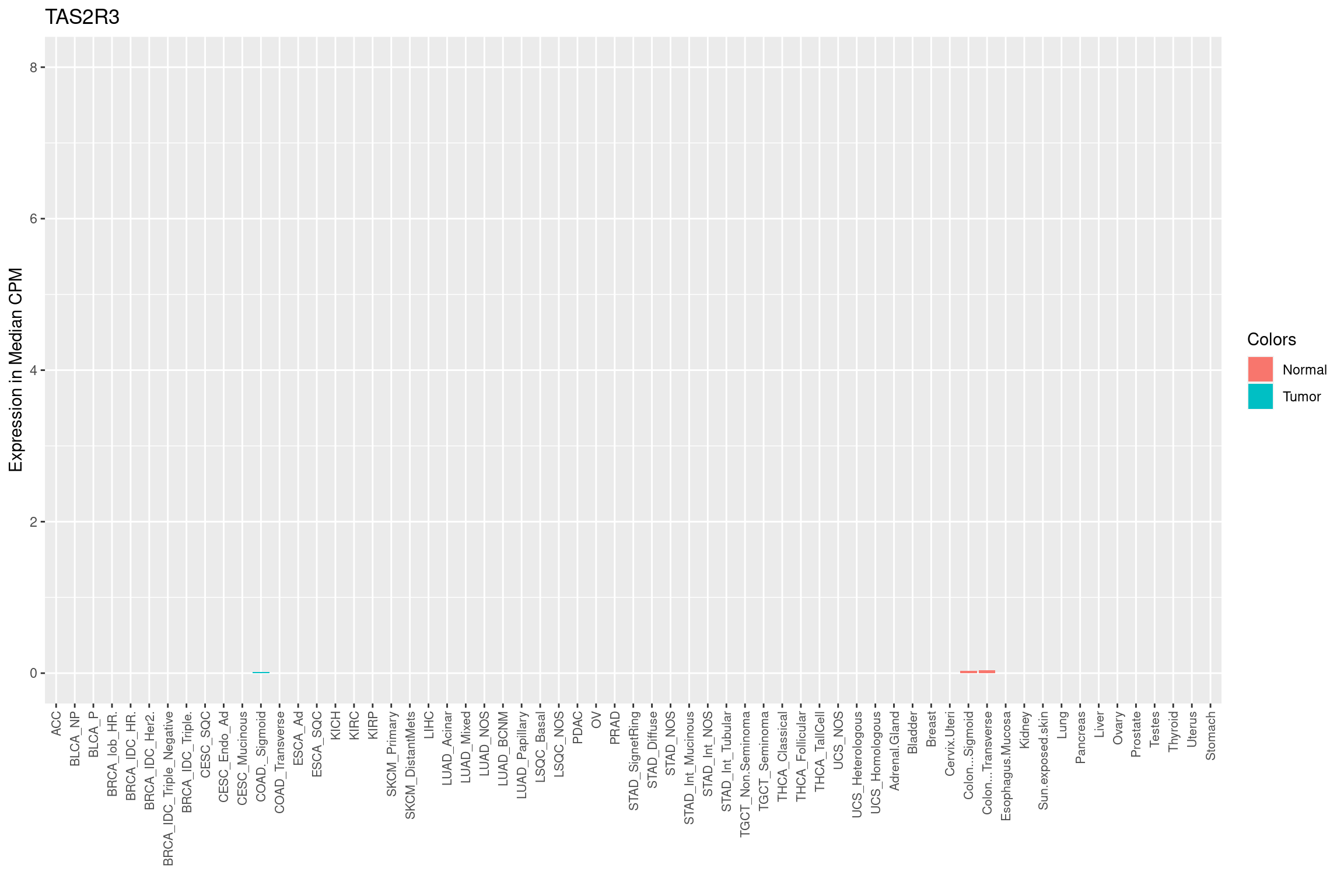

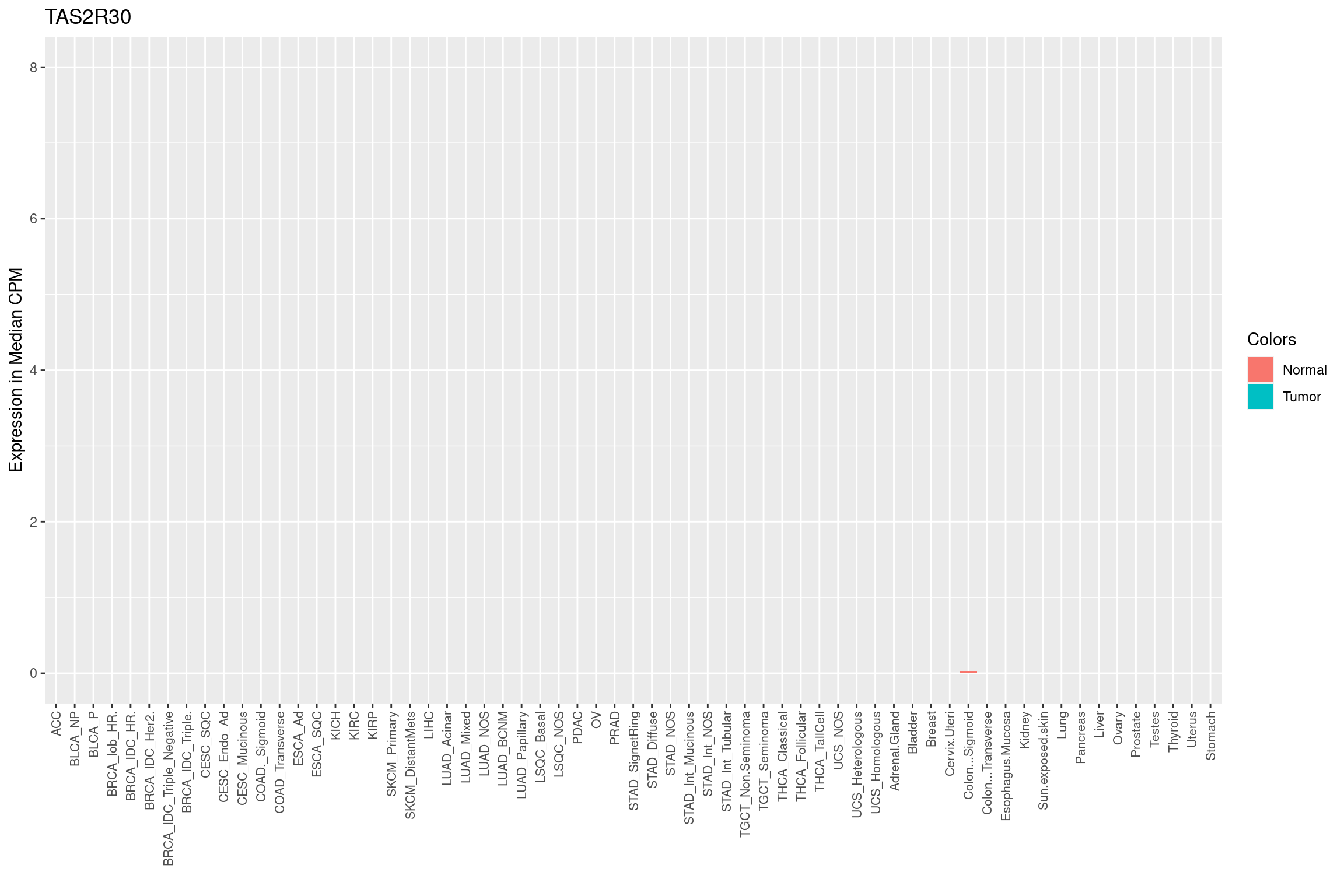

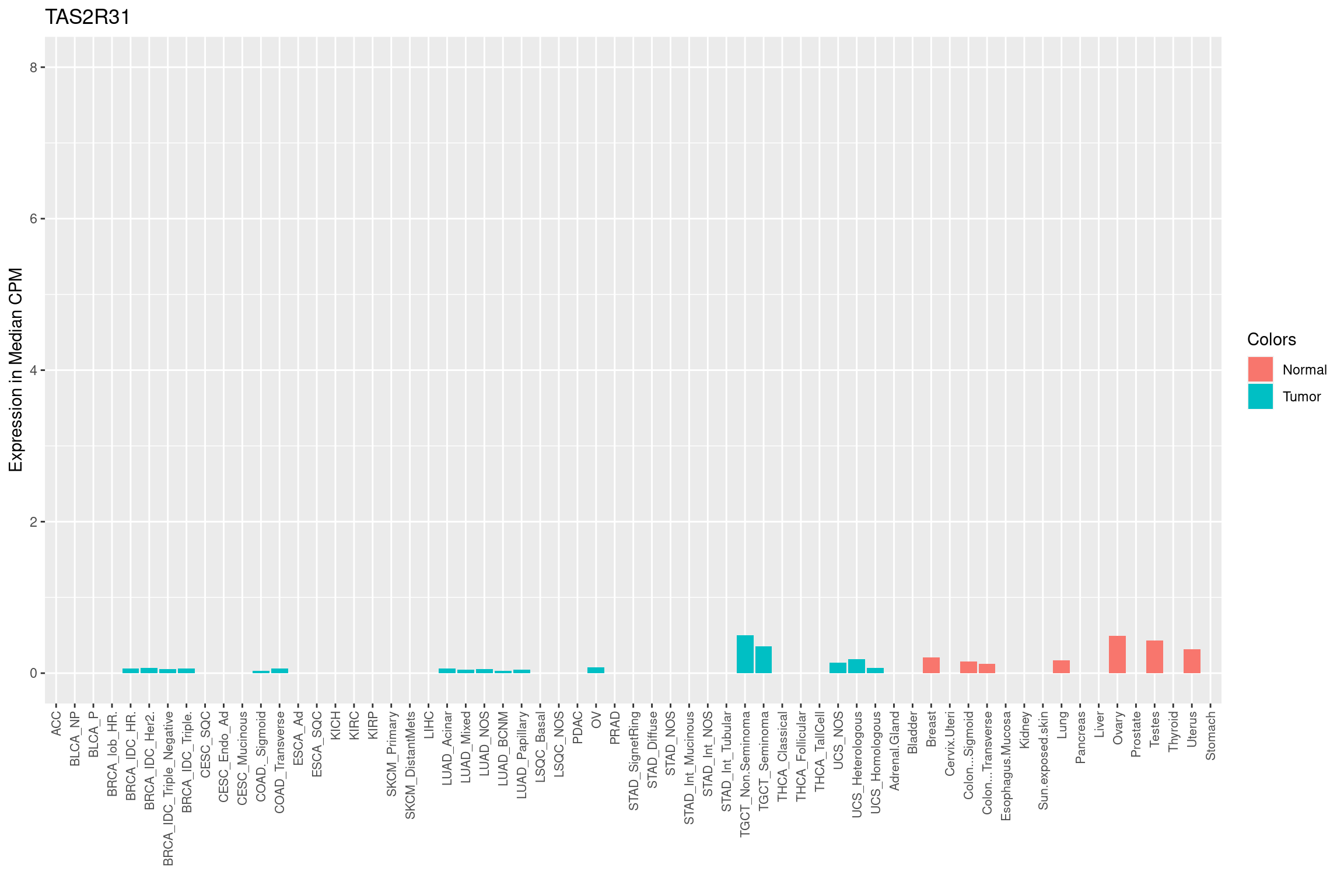

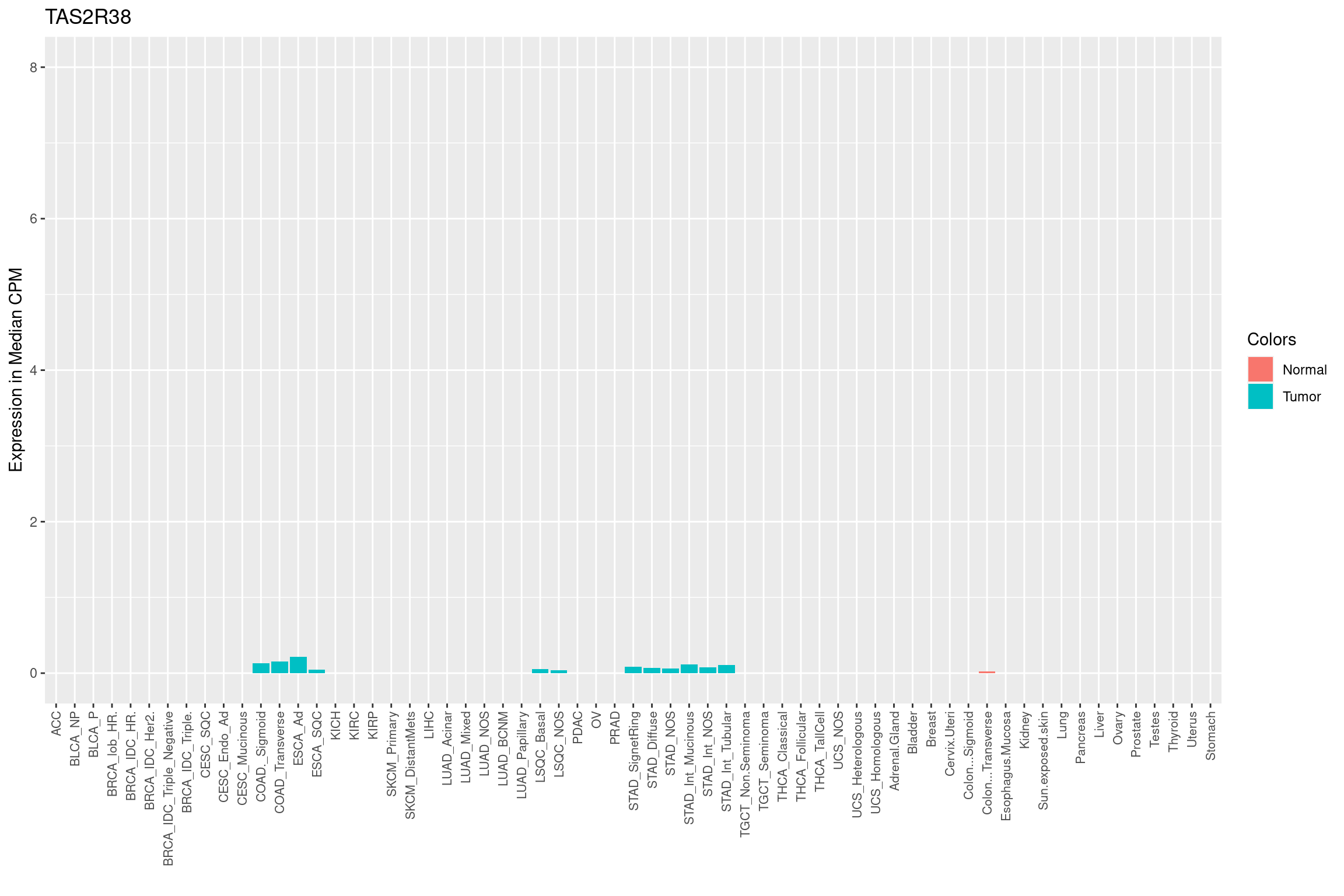

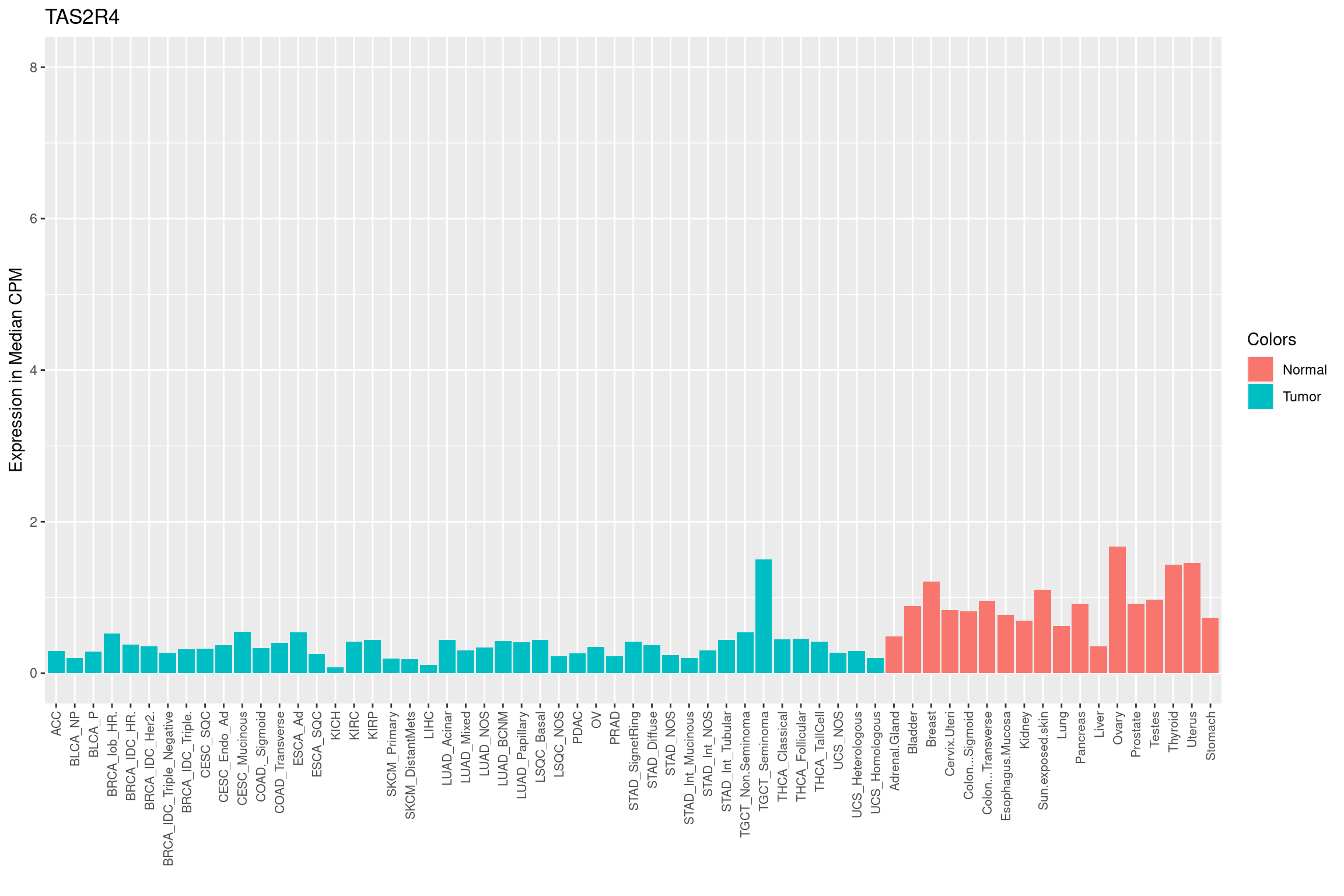

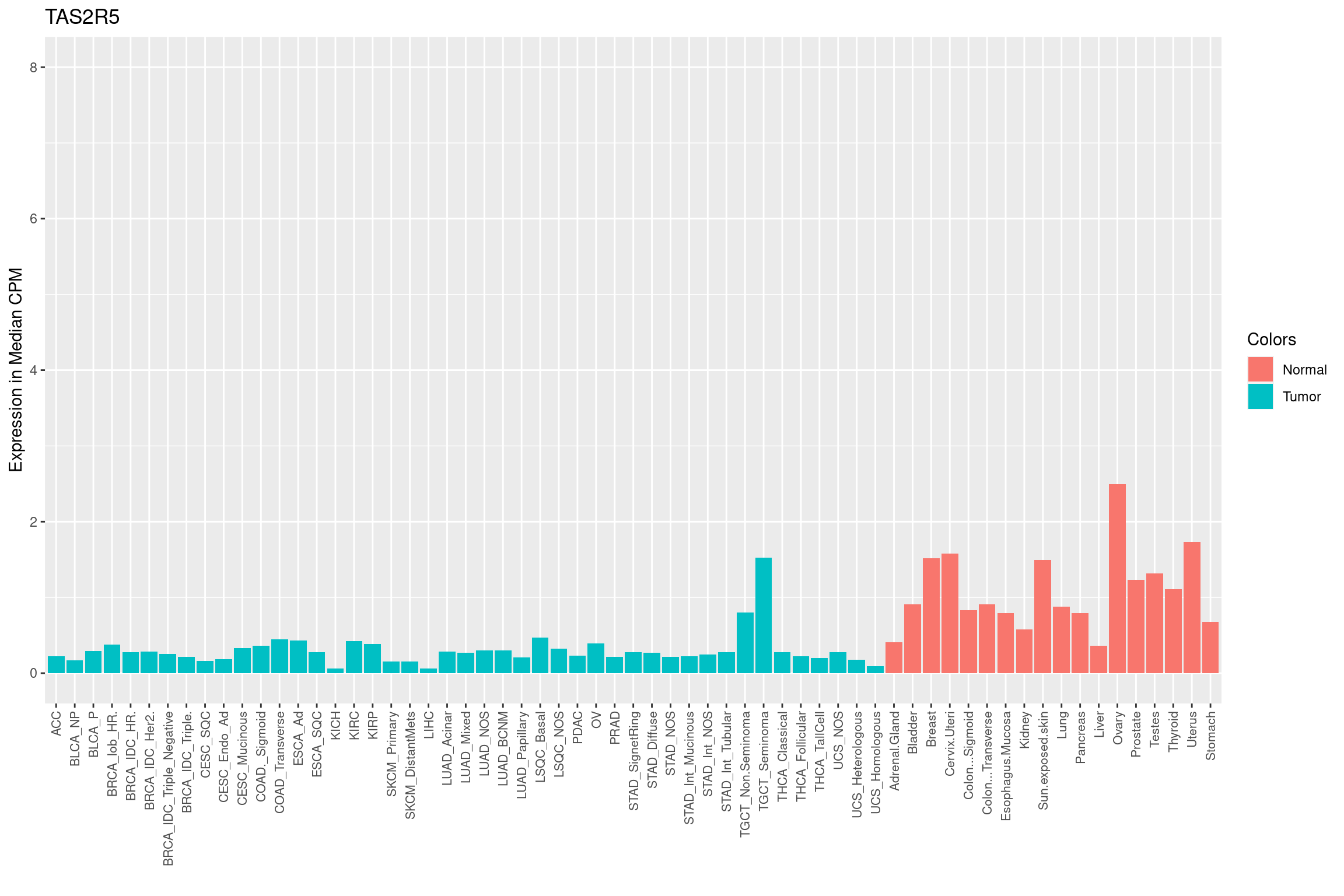

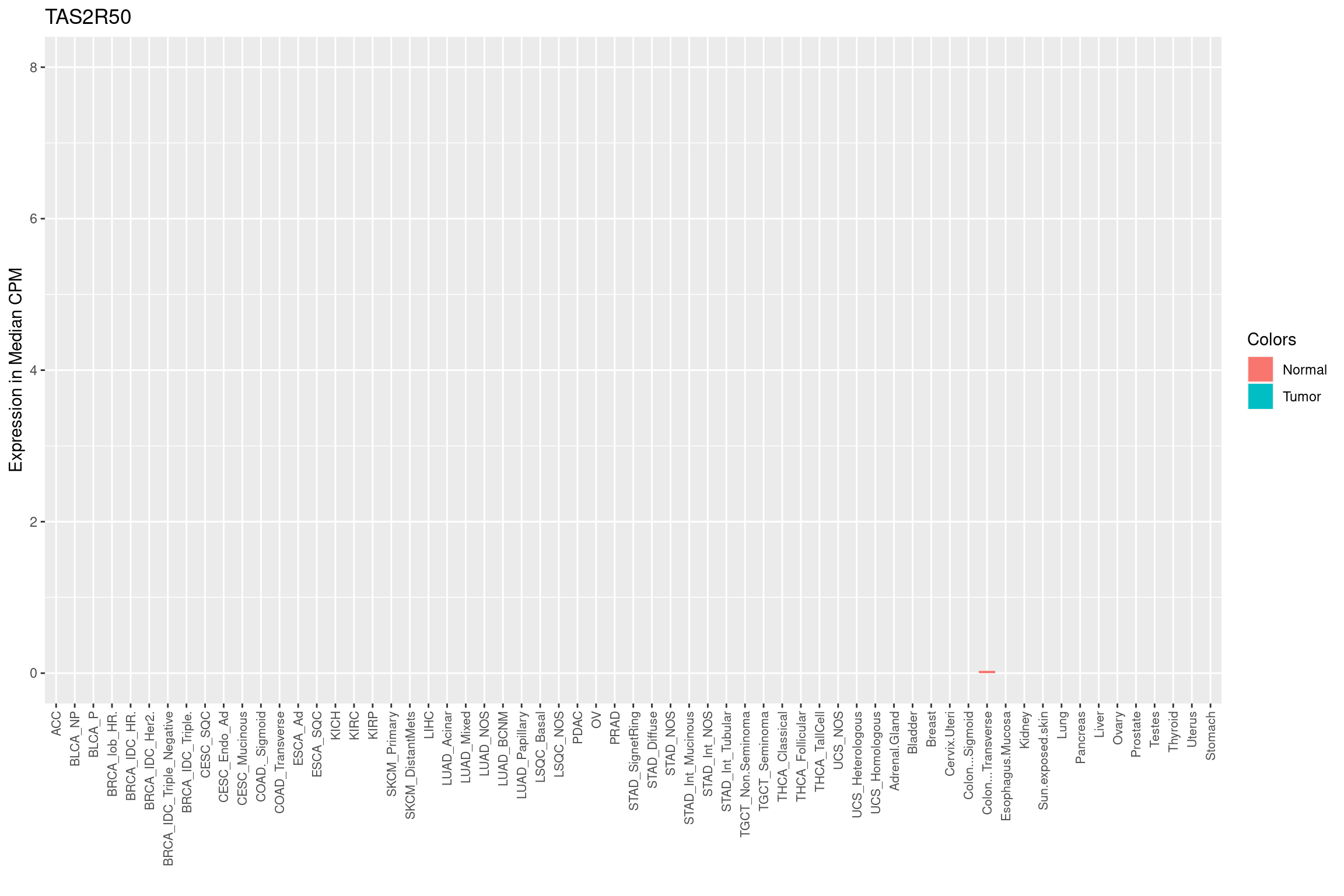


**Supplemental Figure 1.** Median expression of *TAS1R* and *TAS2R* genes across solid tumor types (blue) and corresponding normal tissue (red). Data is normalized in counts per million, allowing for comparison between benign and malignant tissue. Only detectable *TAS1R* and *TAS2R* genes are included. CPM, Counts Per Million. Plots generated using files from <https://insellab.github.io/>^47^ and labeled using The Cancer Genome Atlas (TCGA) cancer types and sub-classifications as defined in Figure 1A.


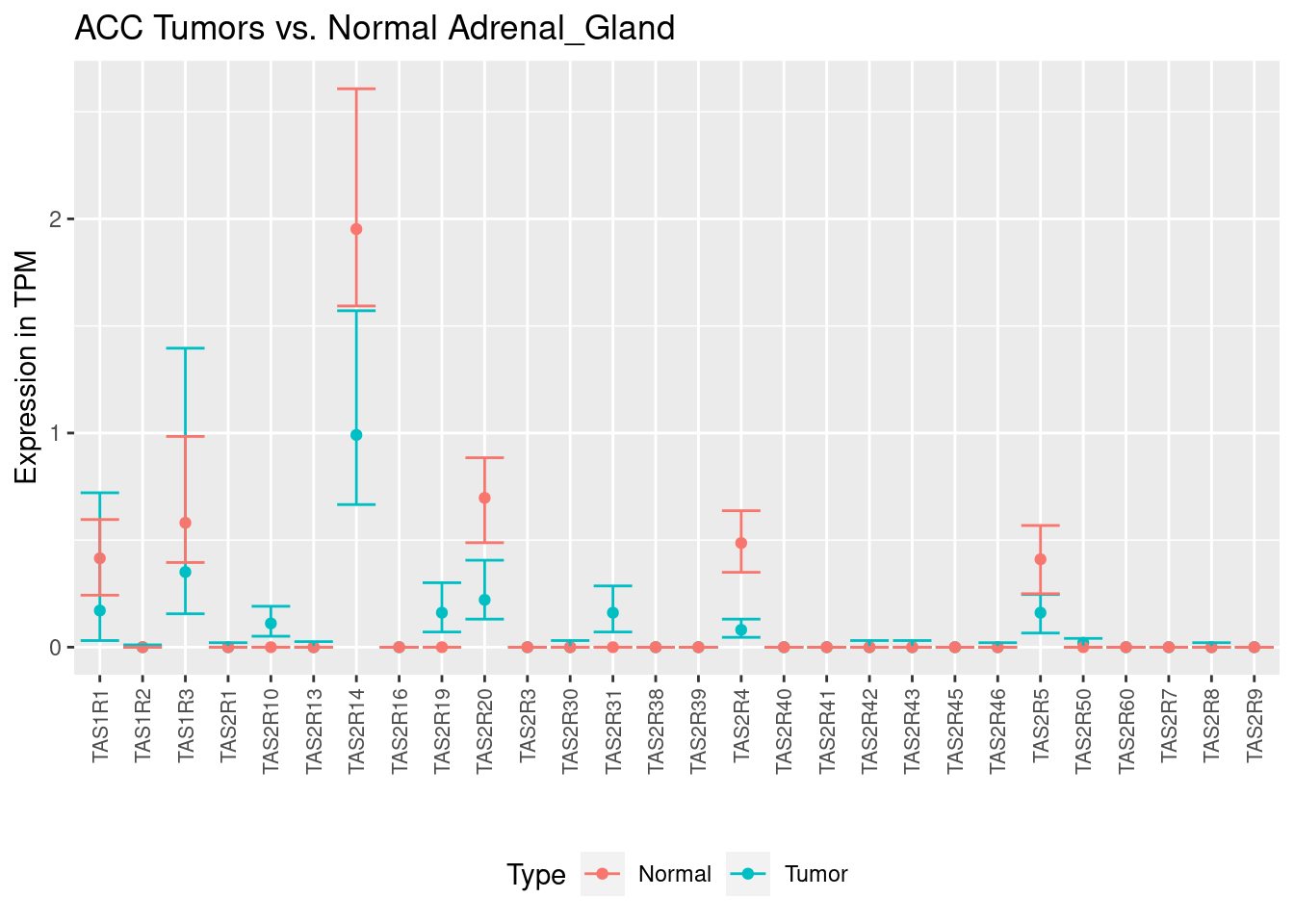

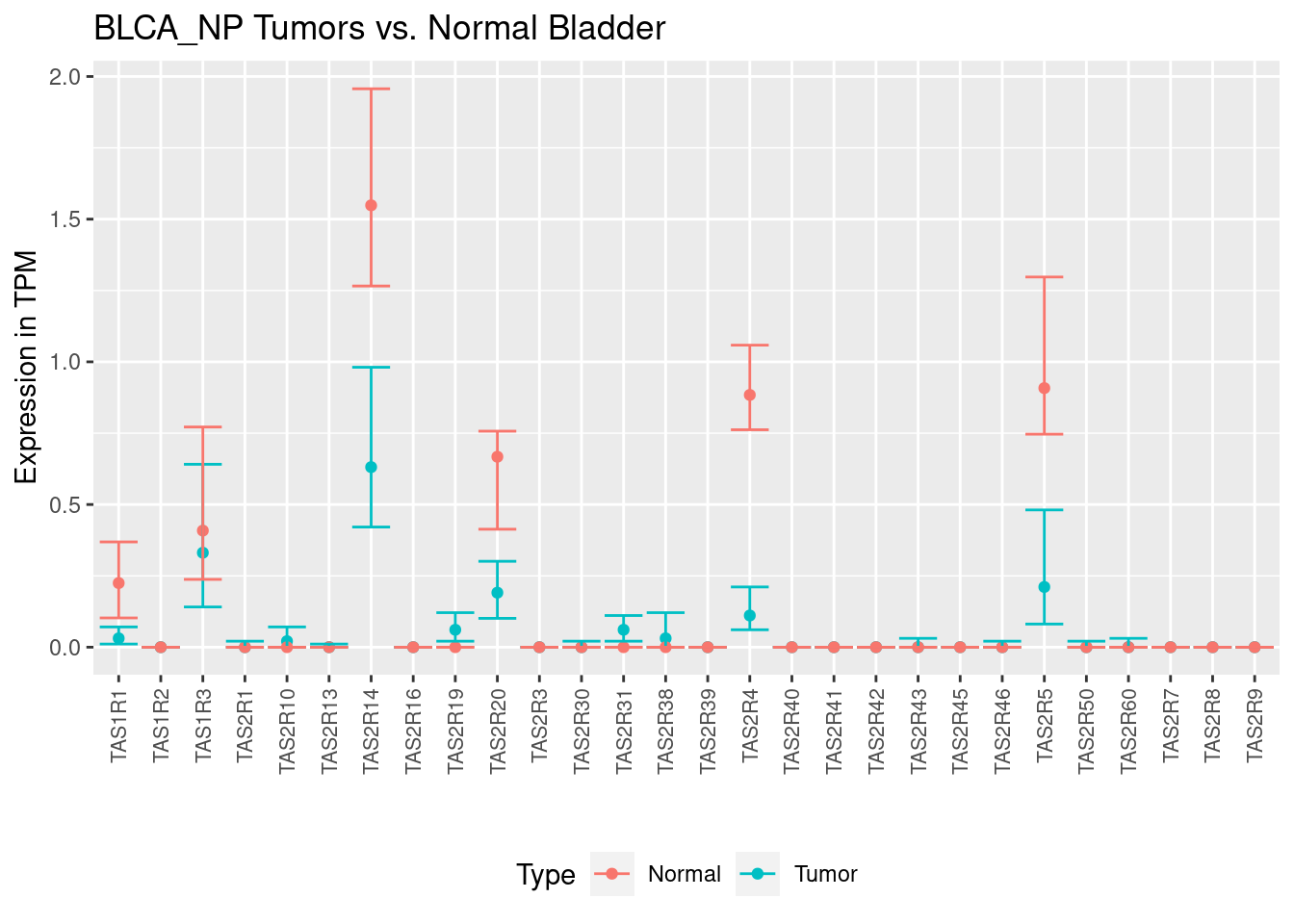

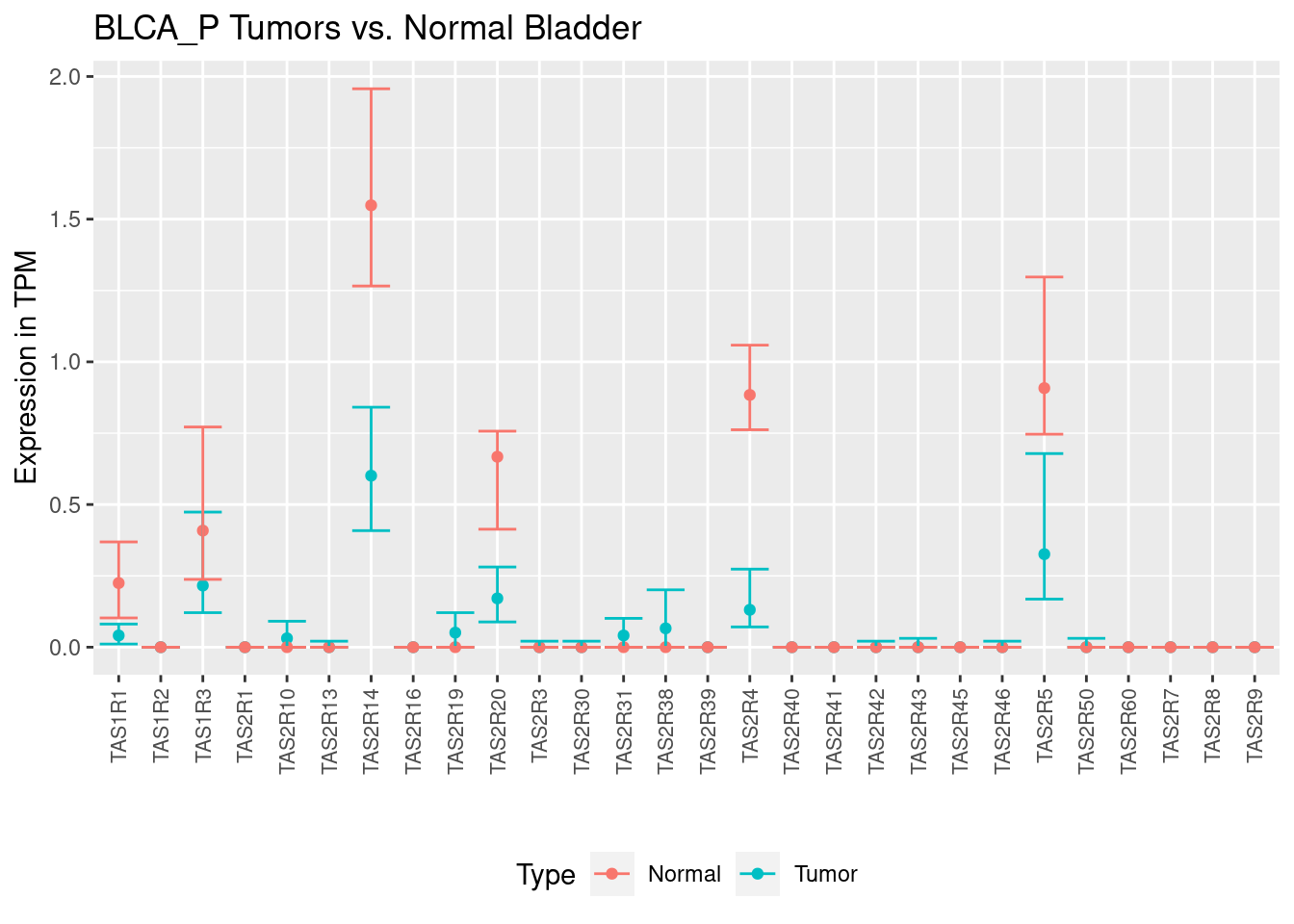

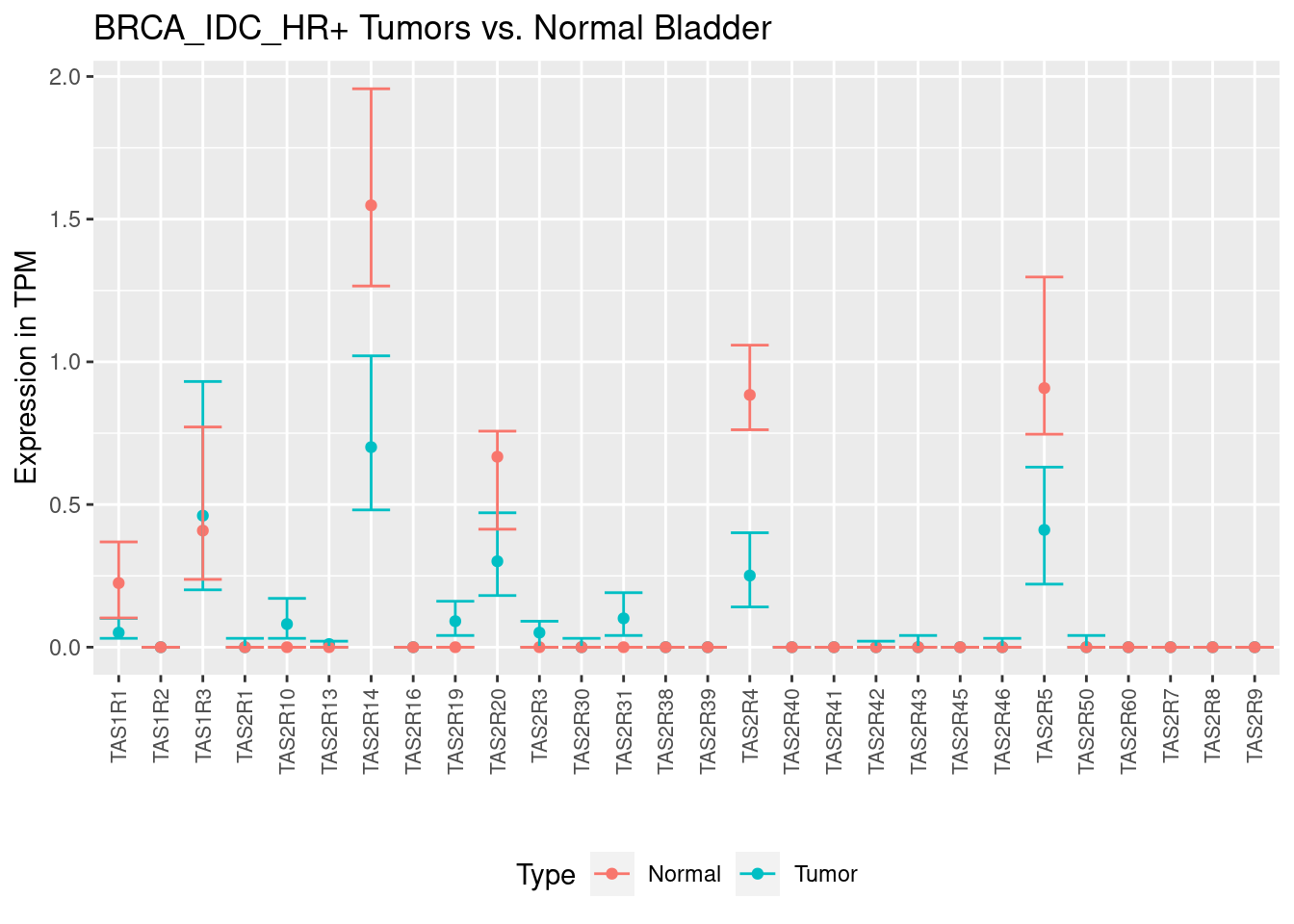

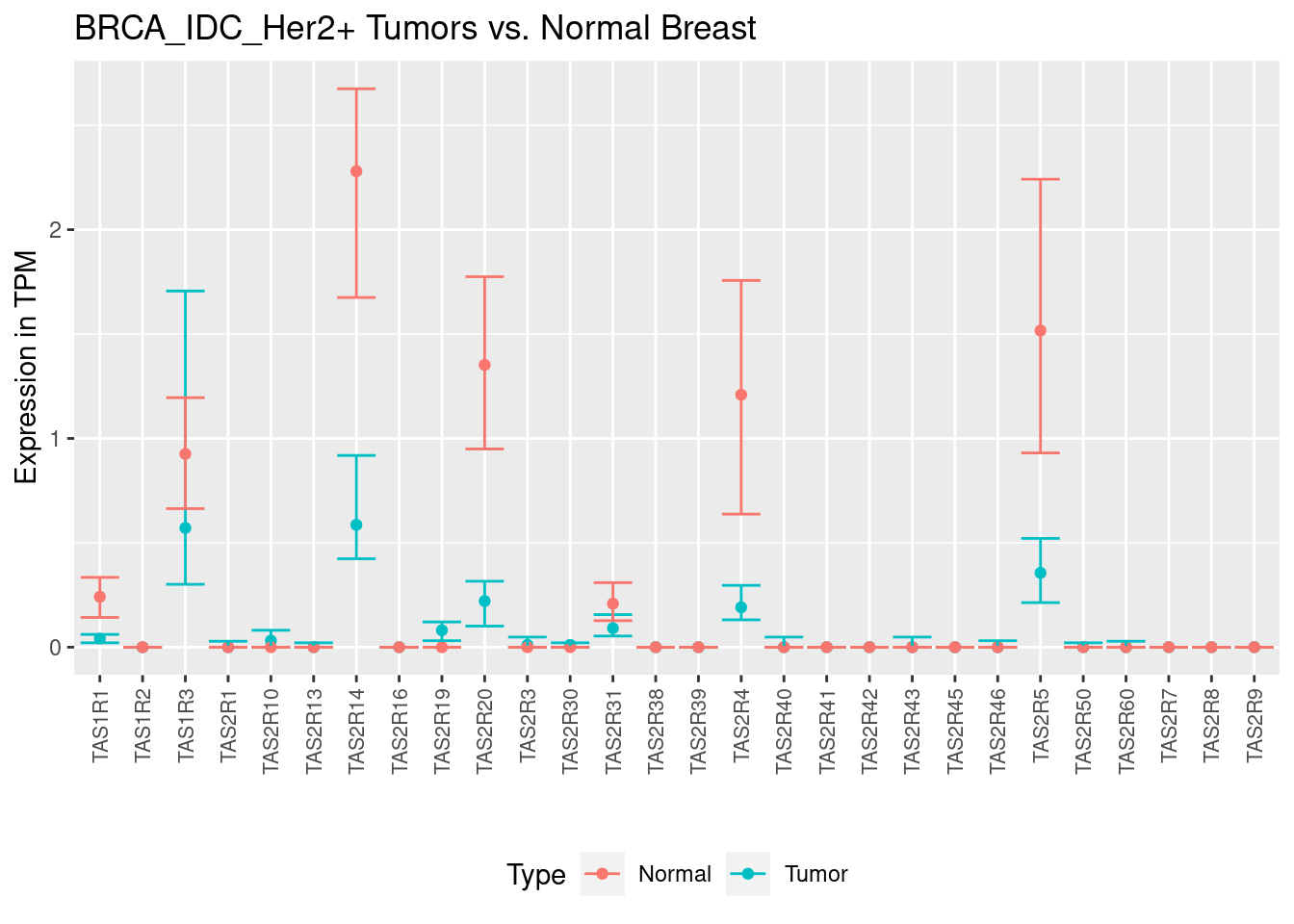

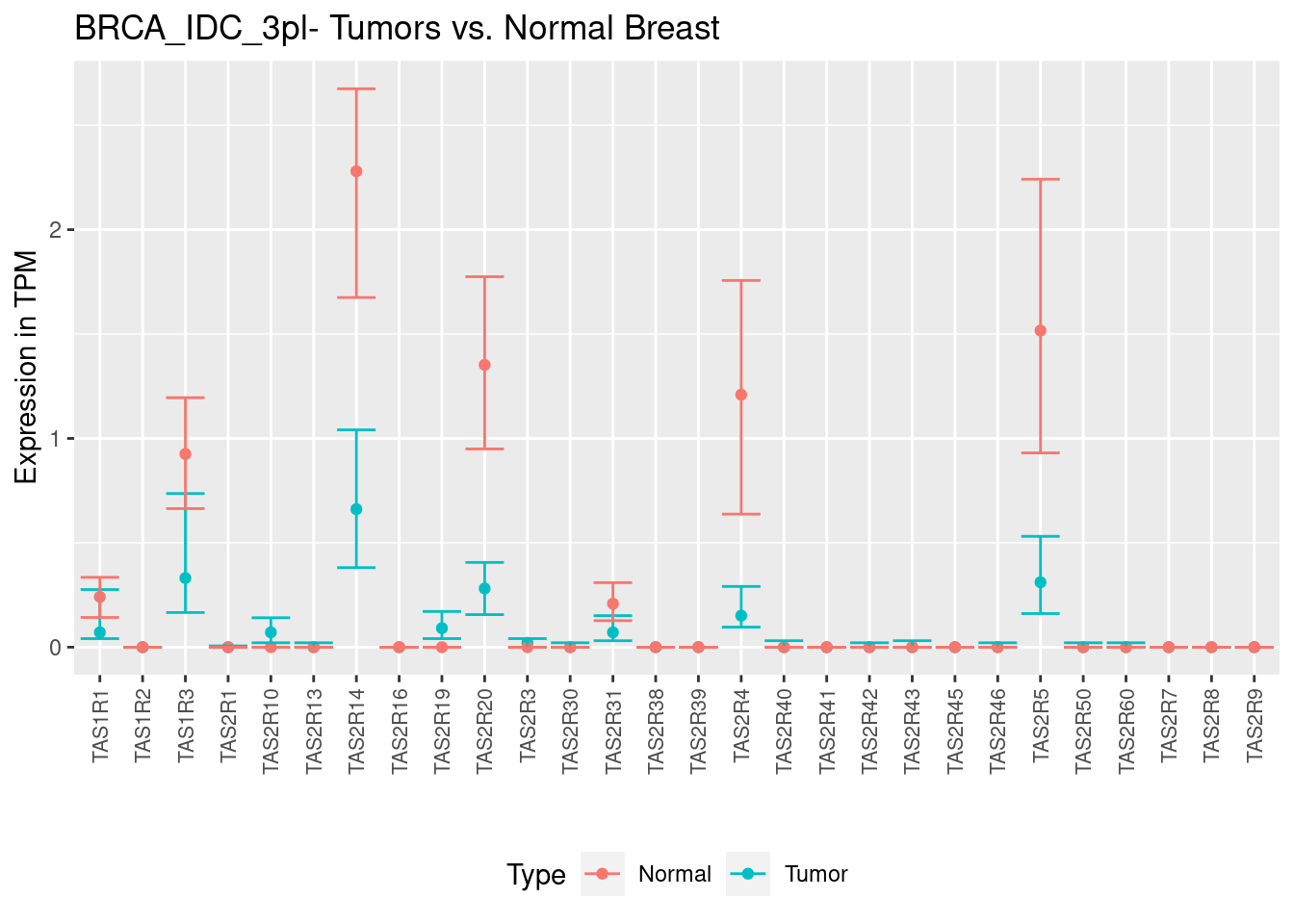

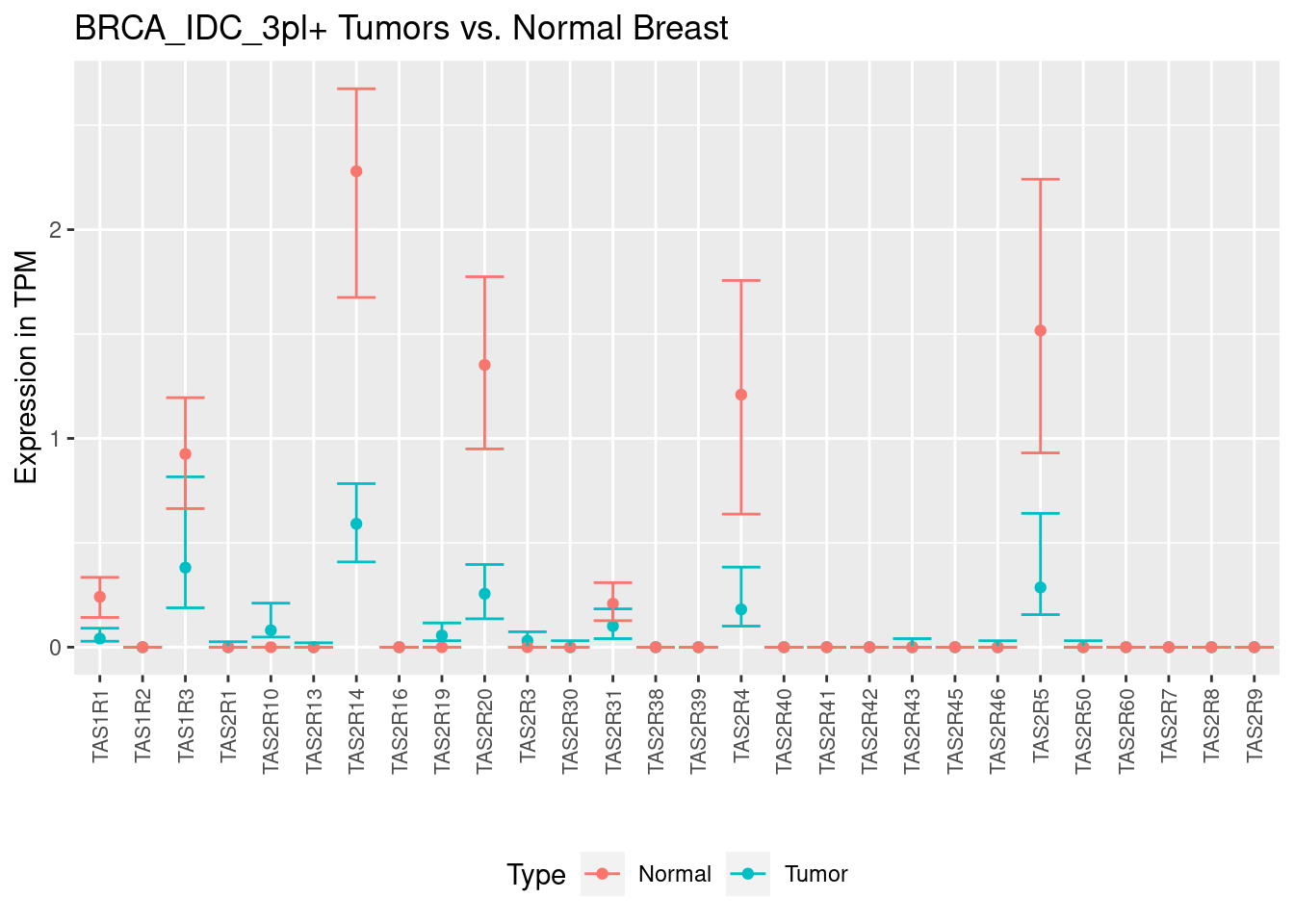

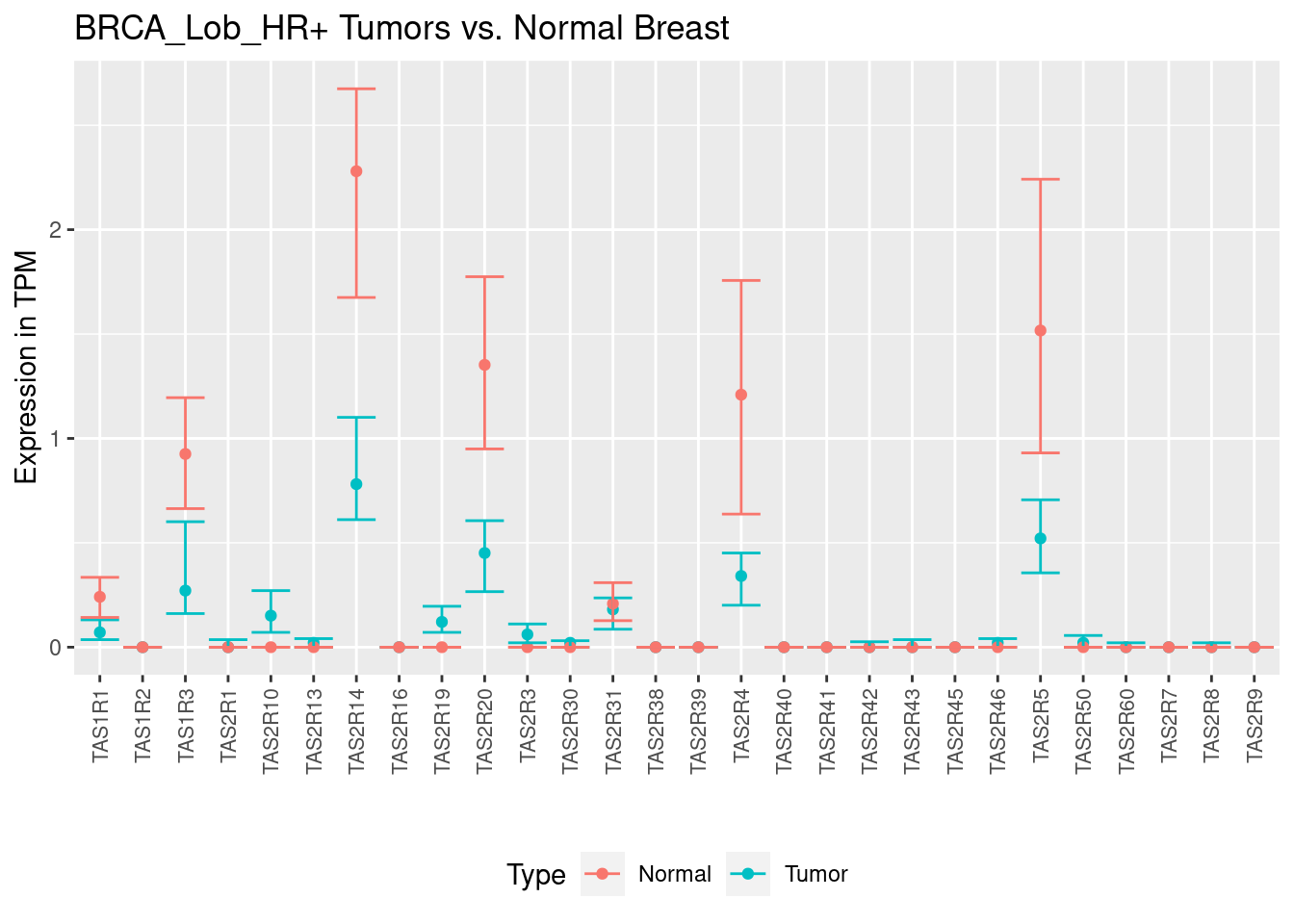

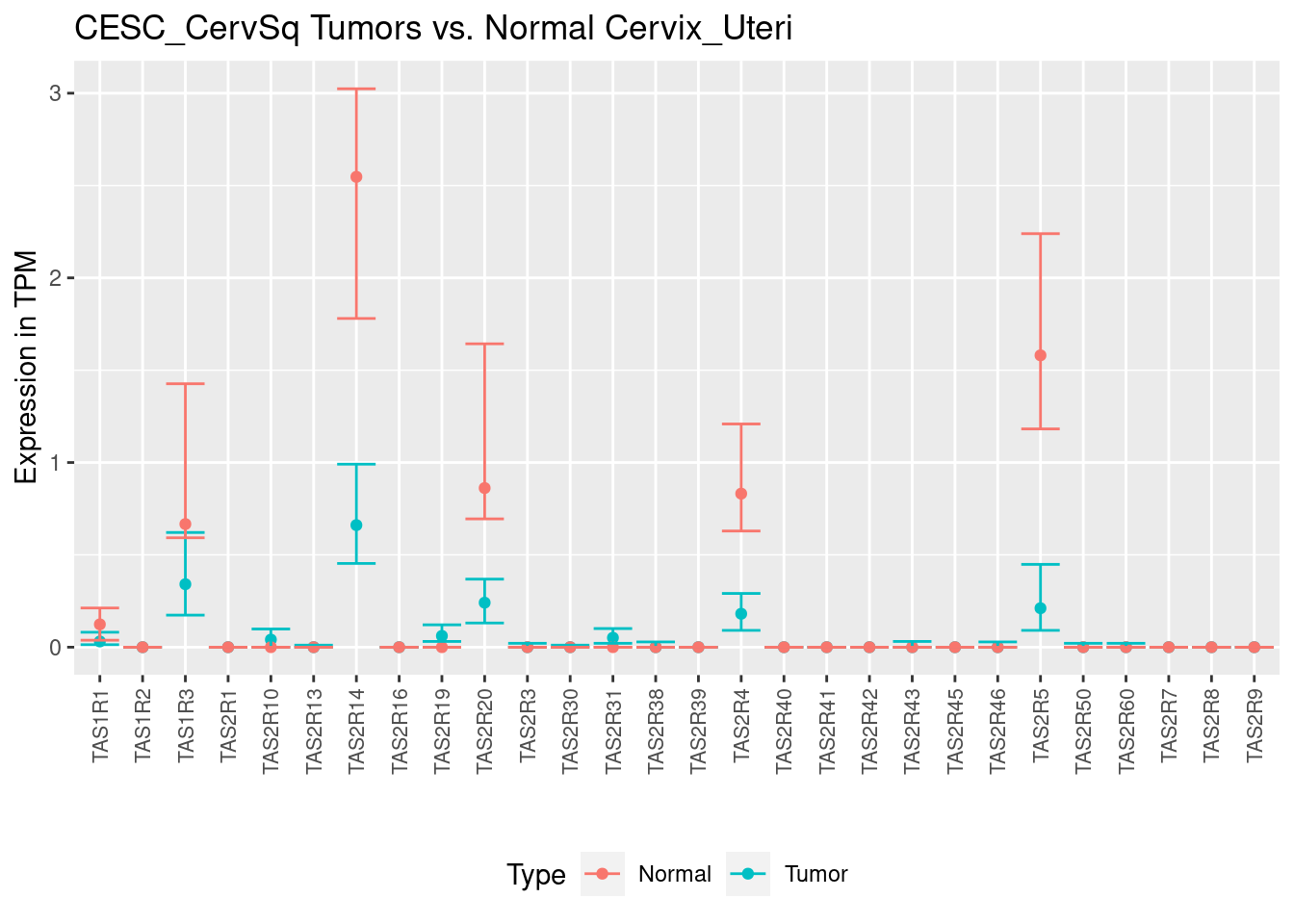

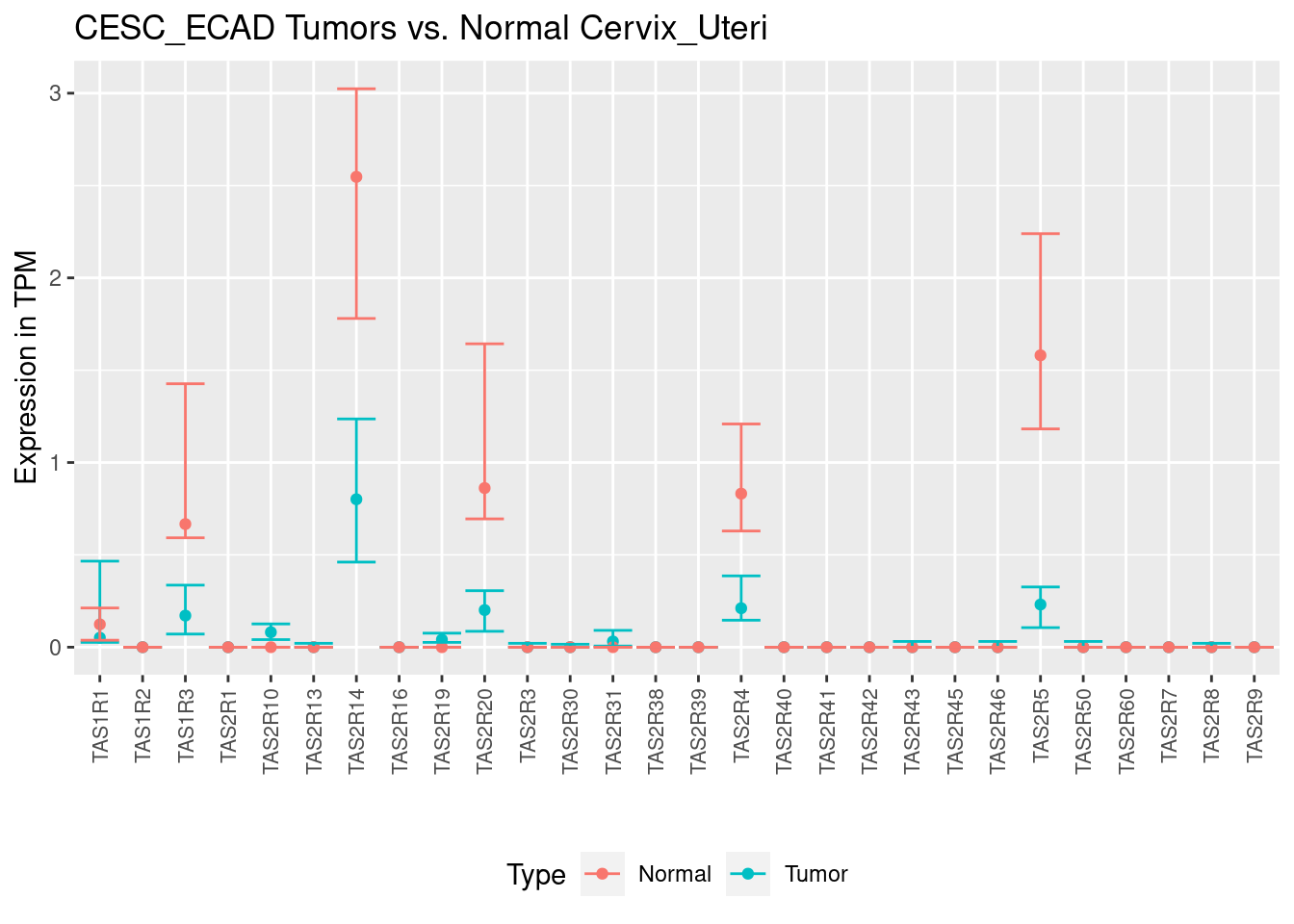

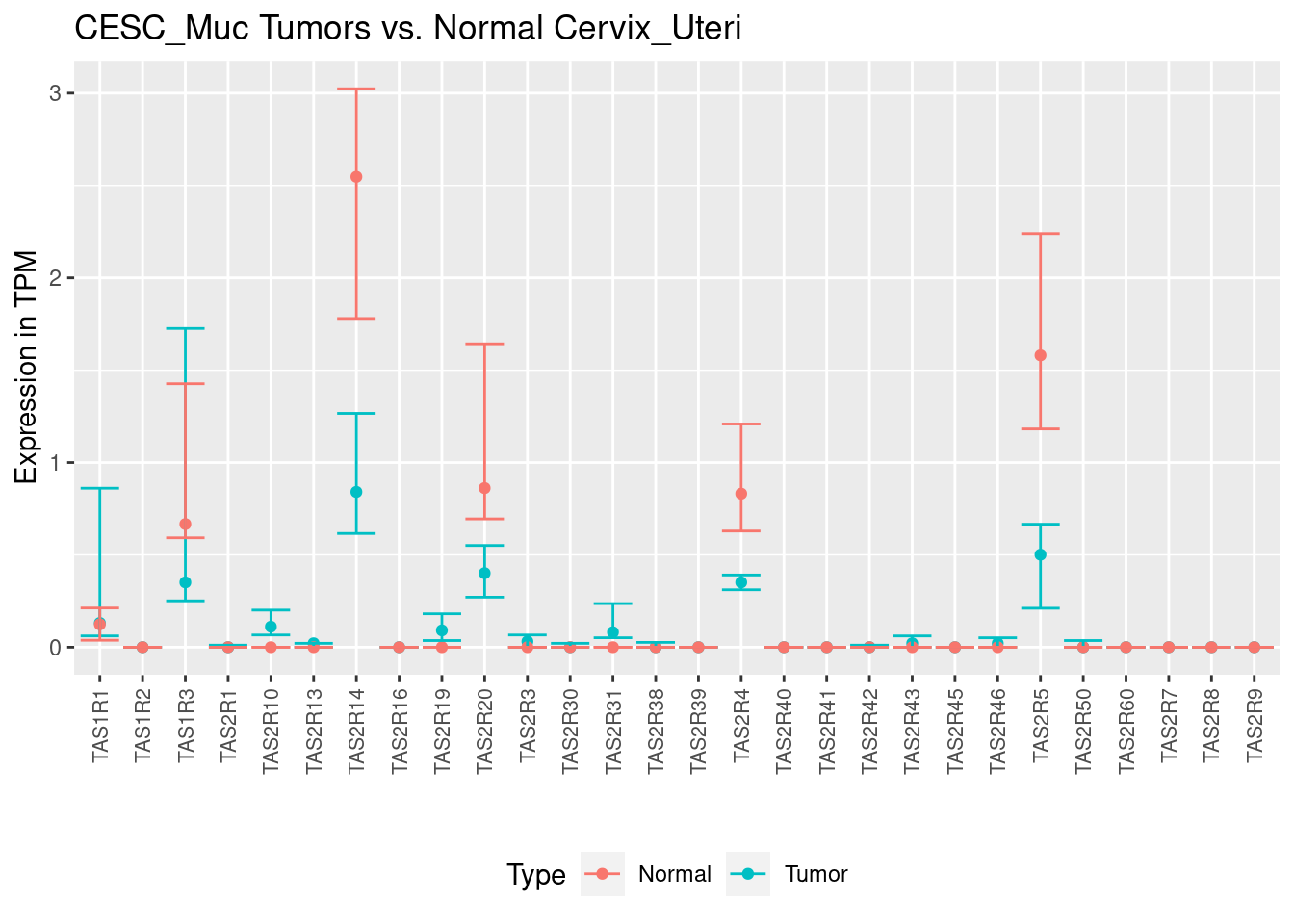

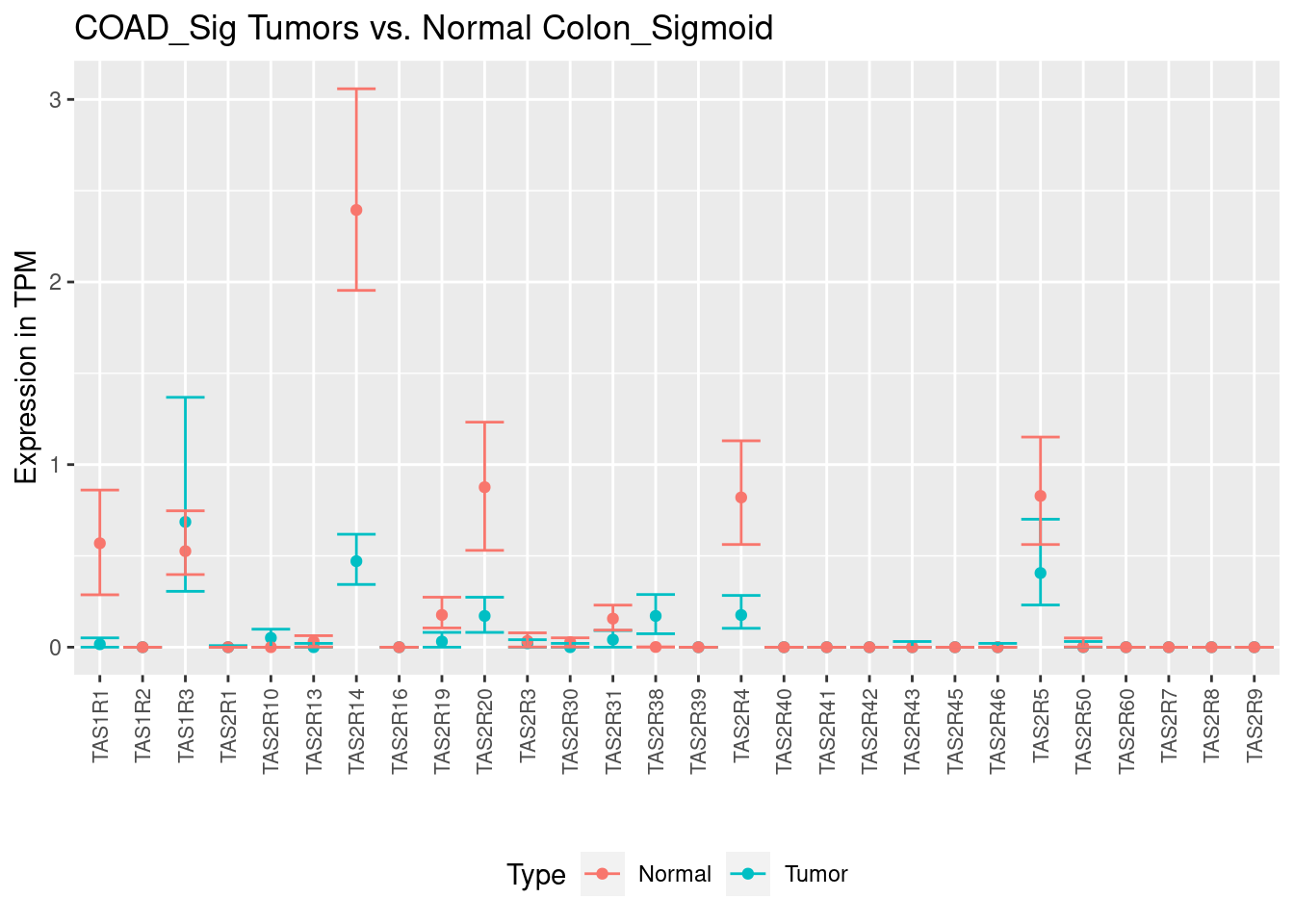

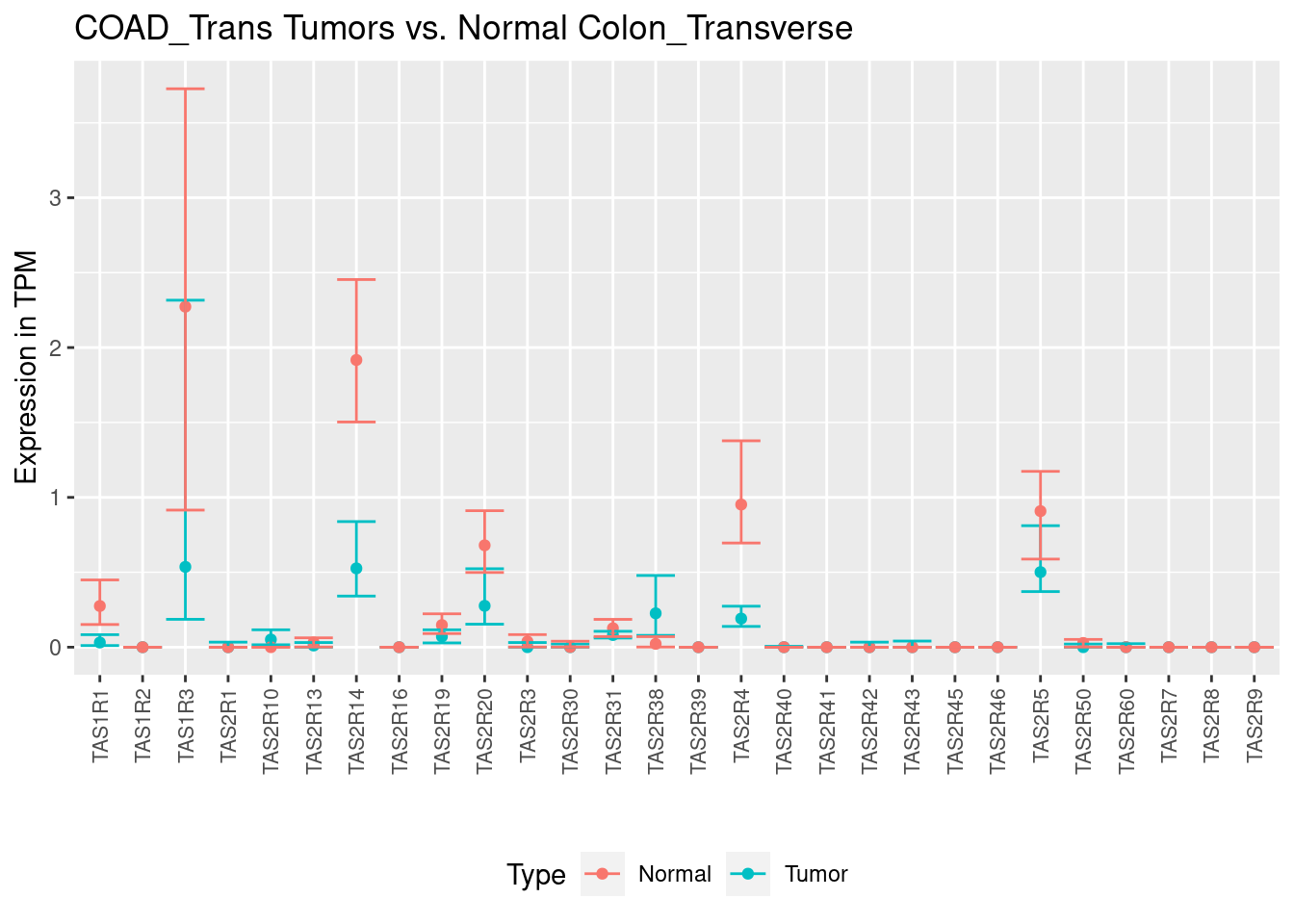

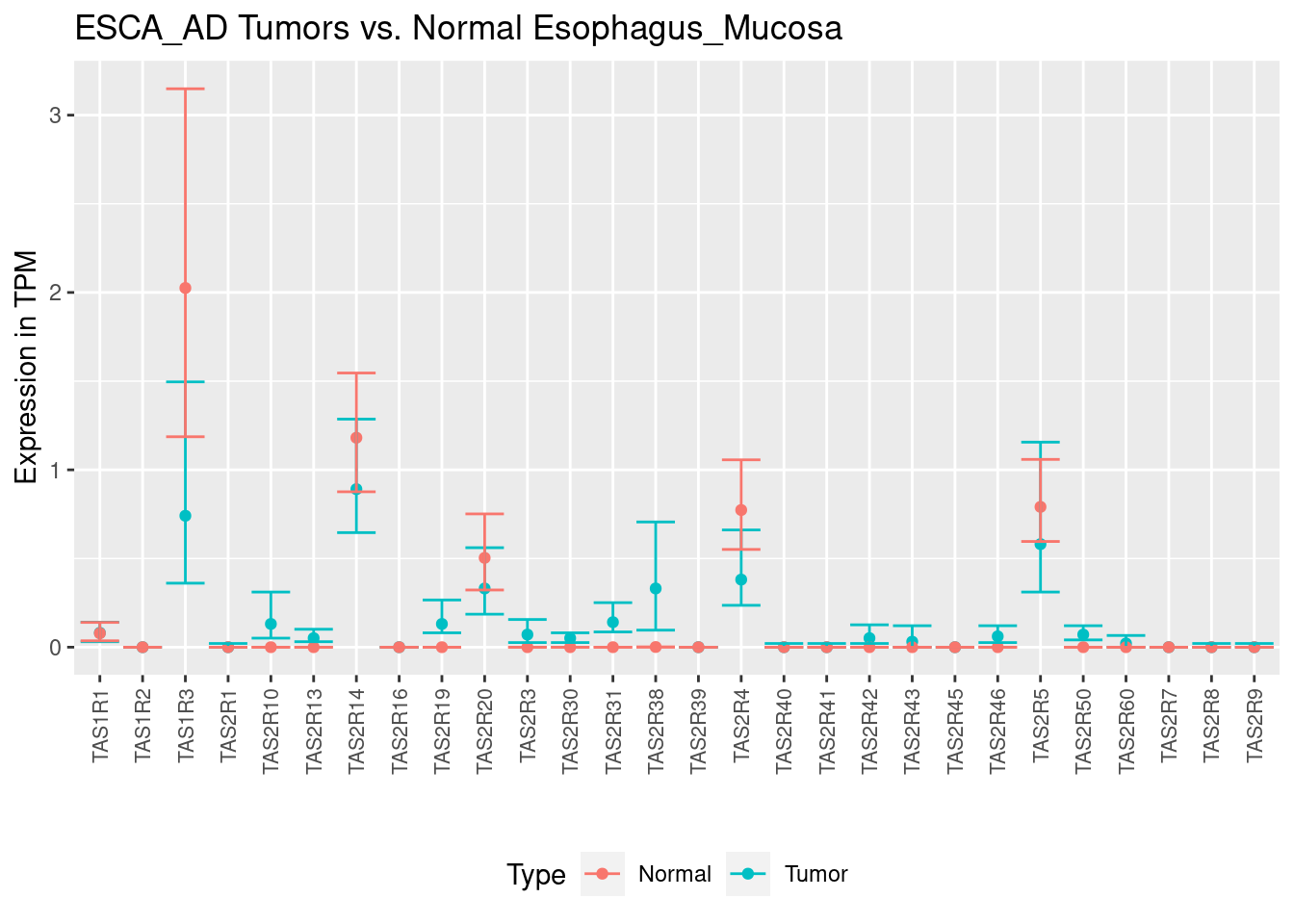

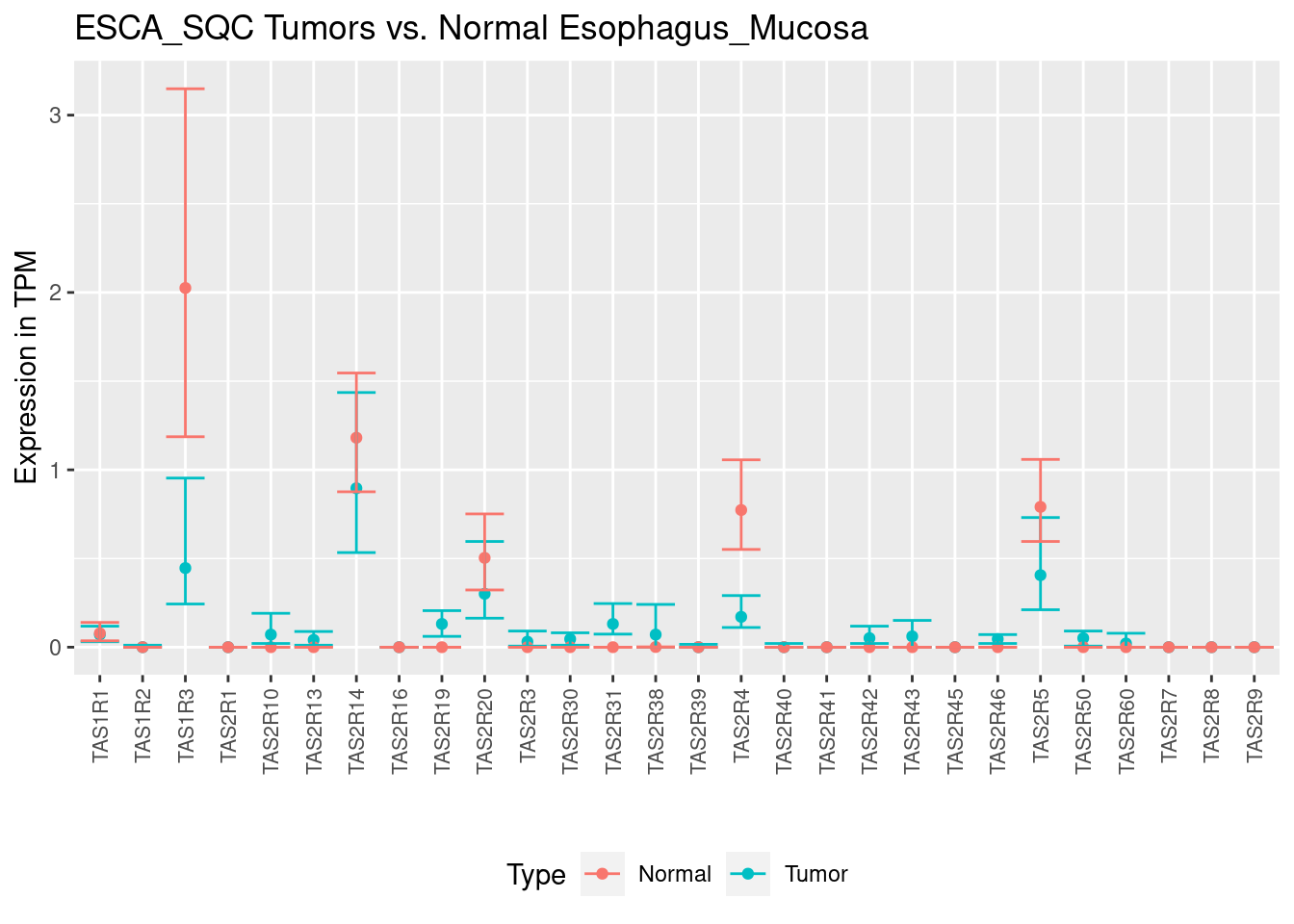

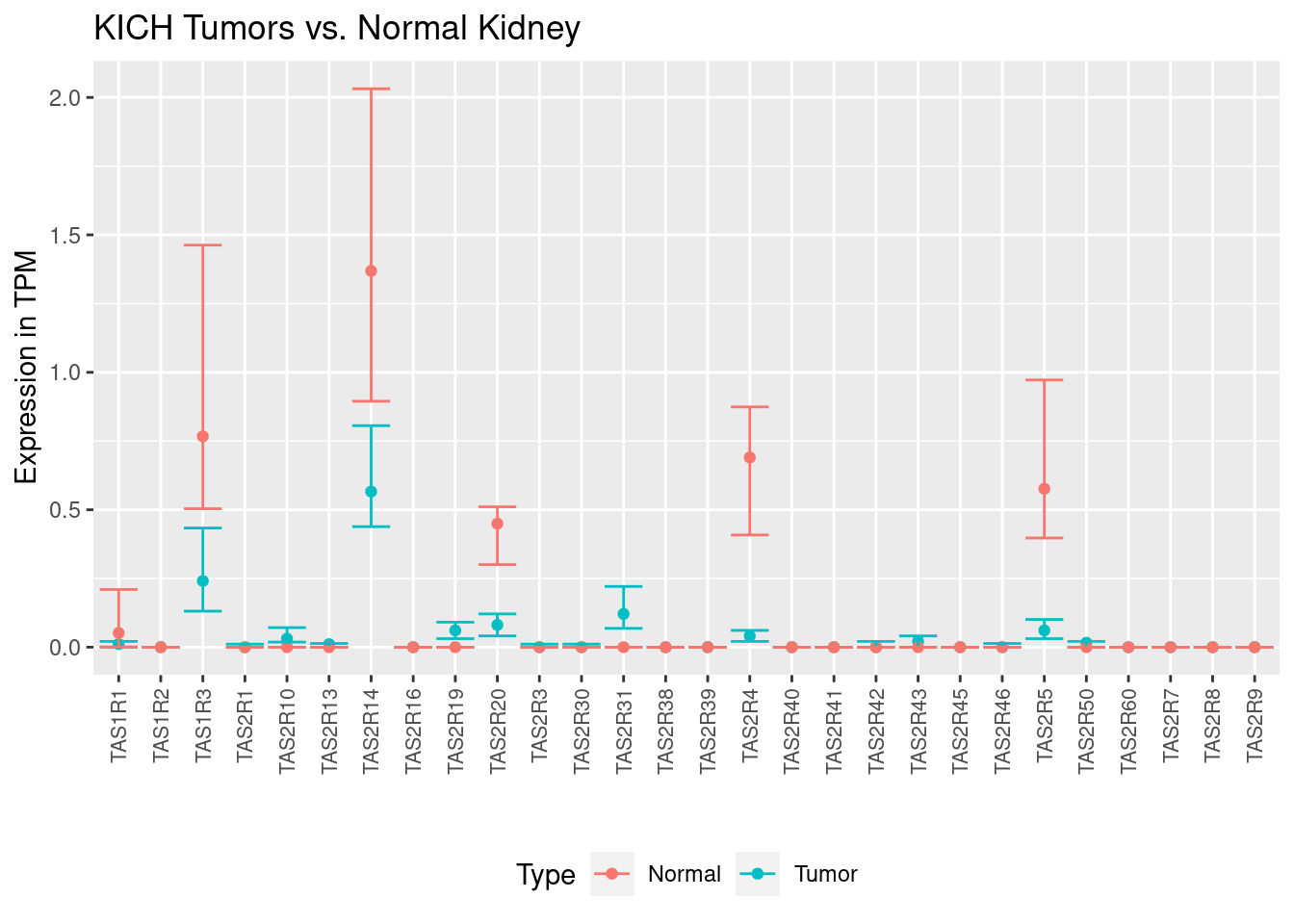

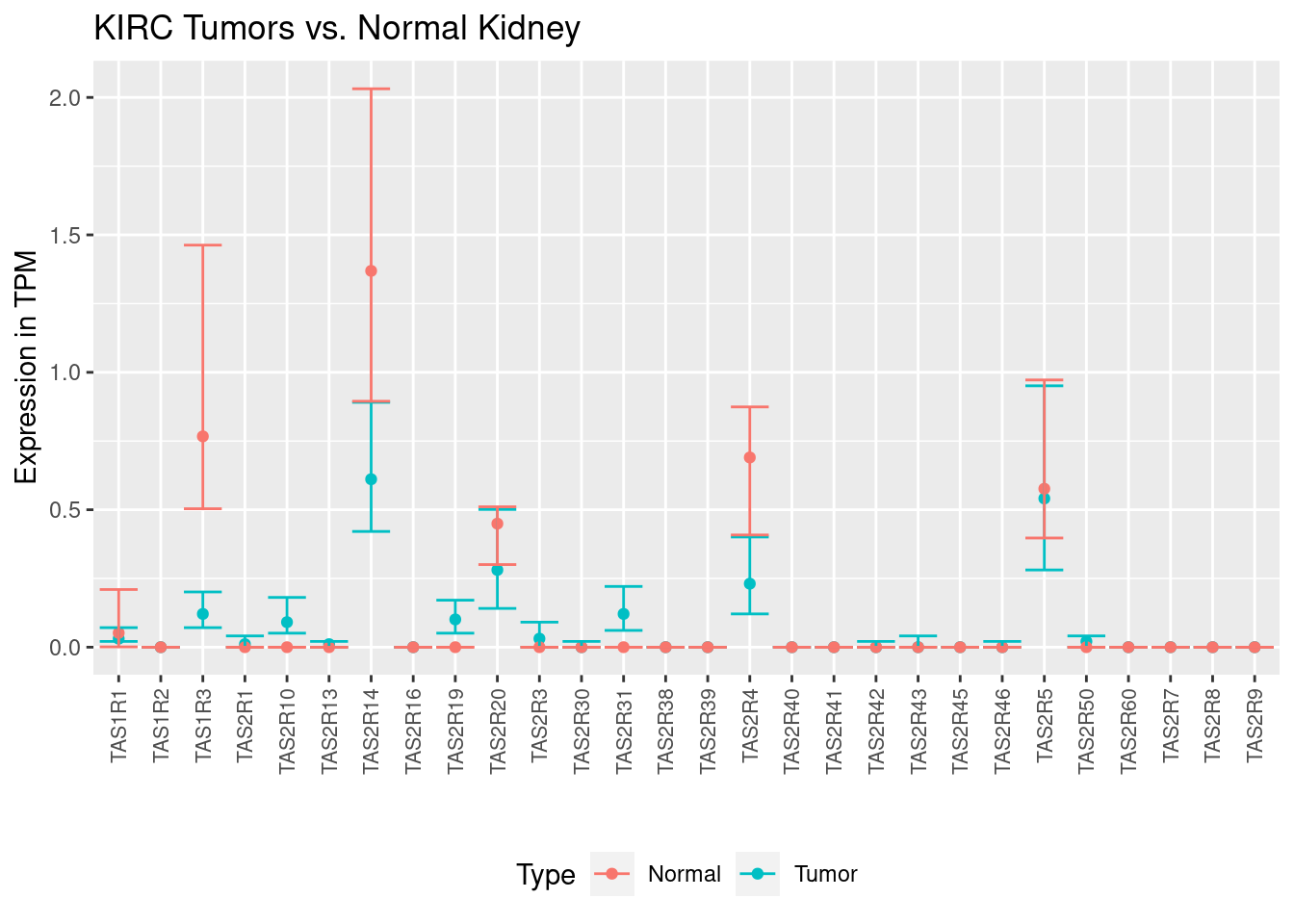

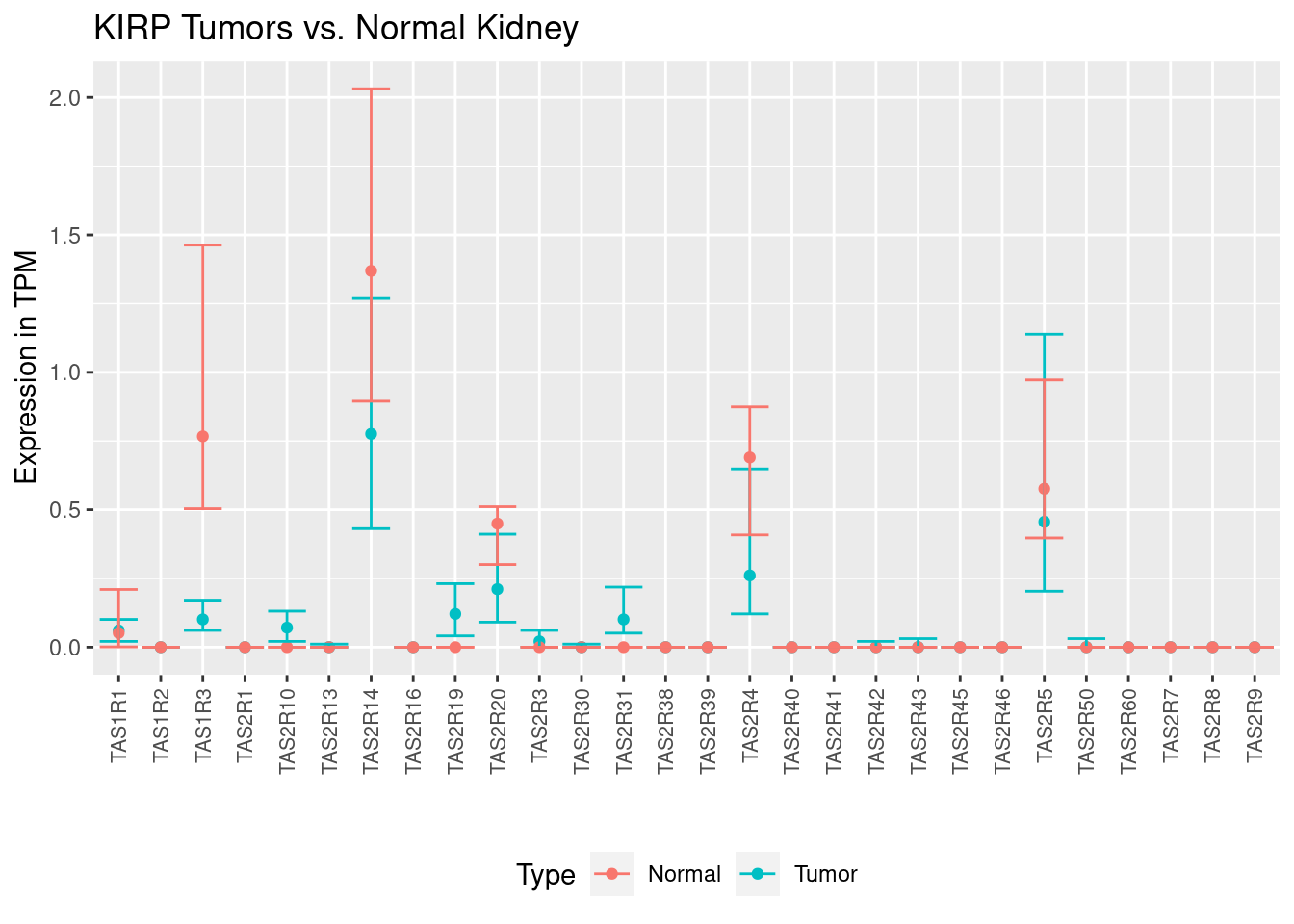

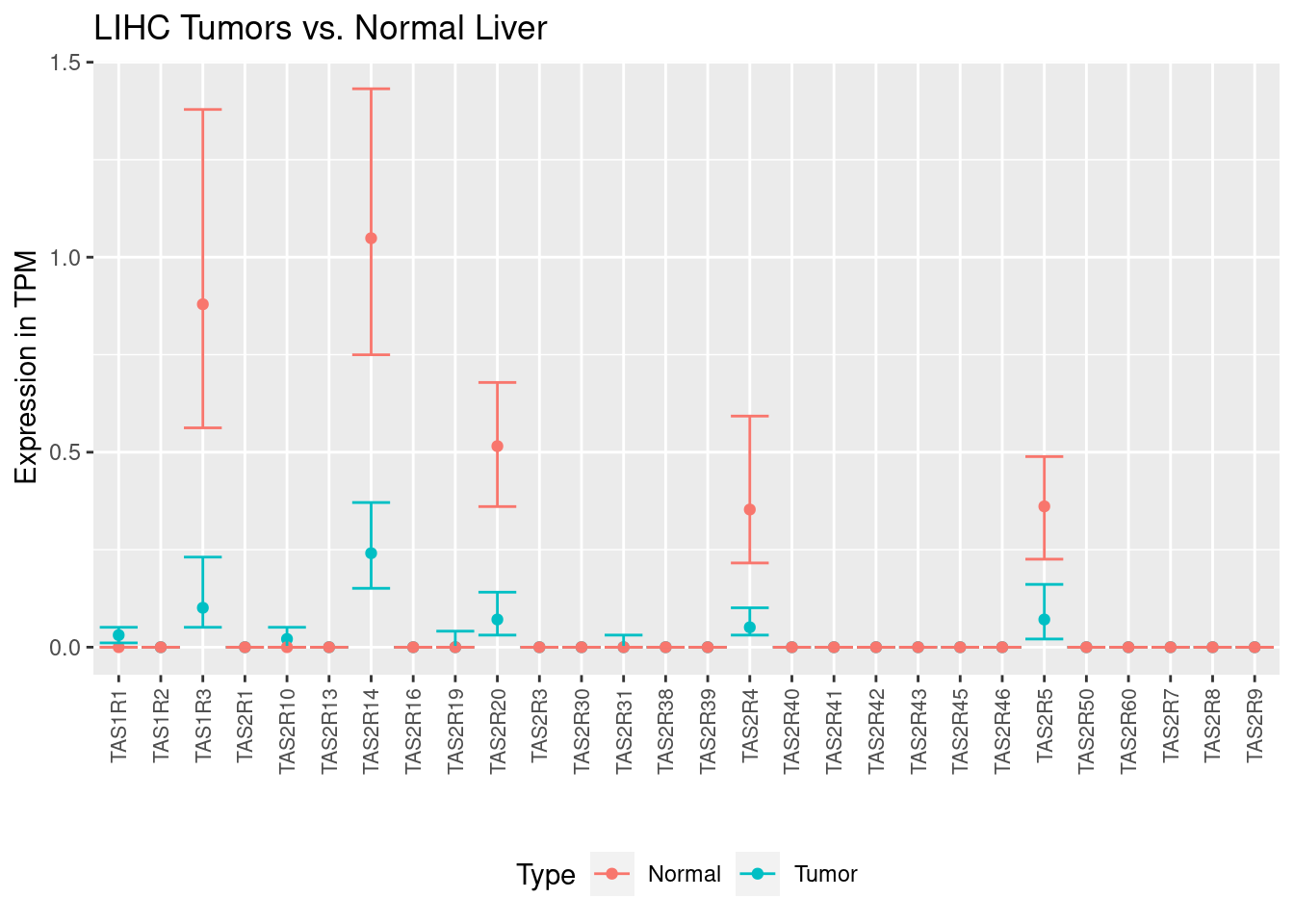

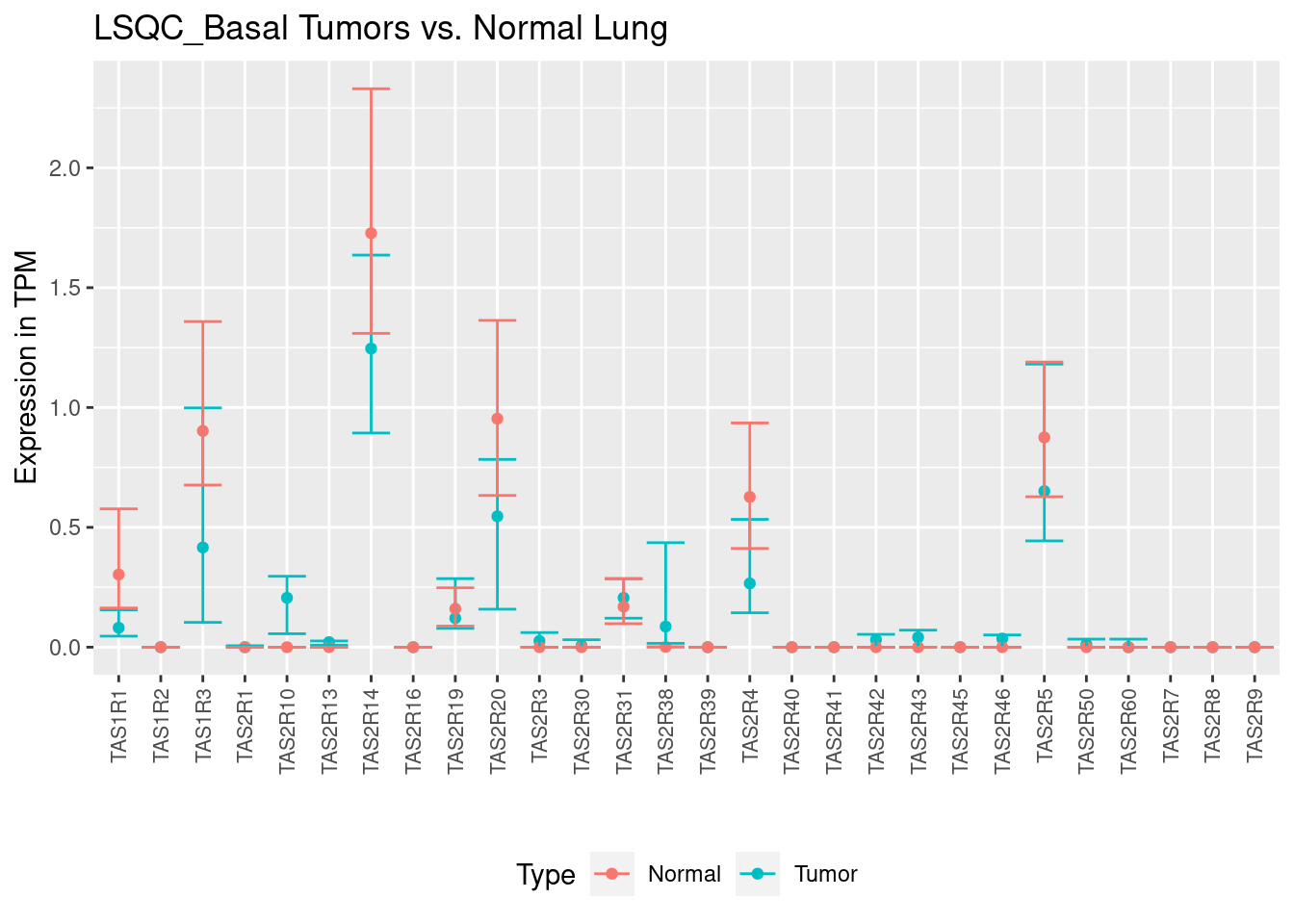

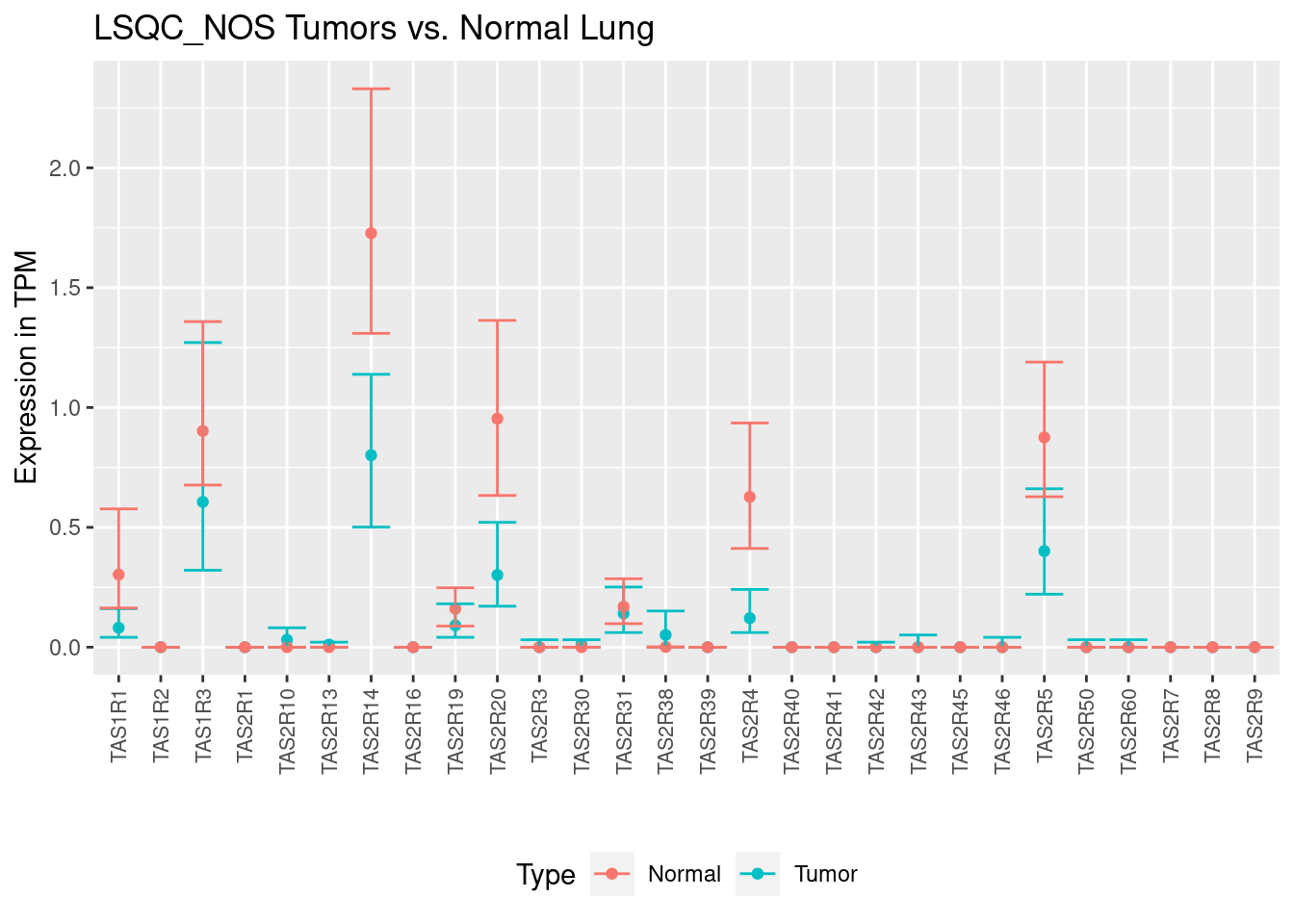

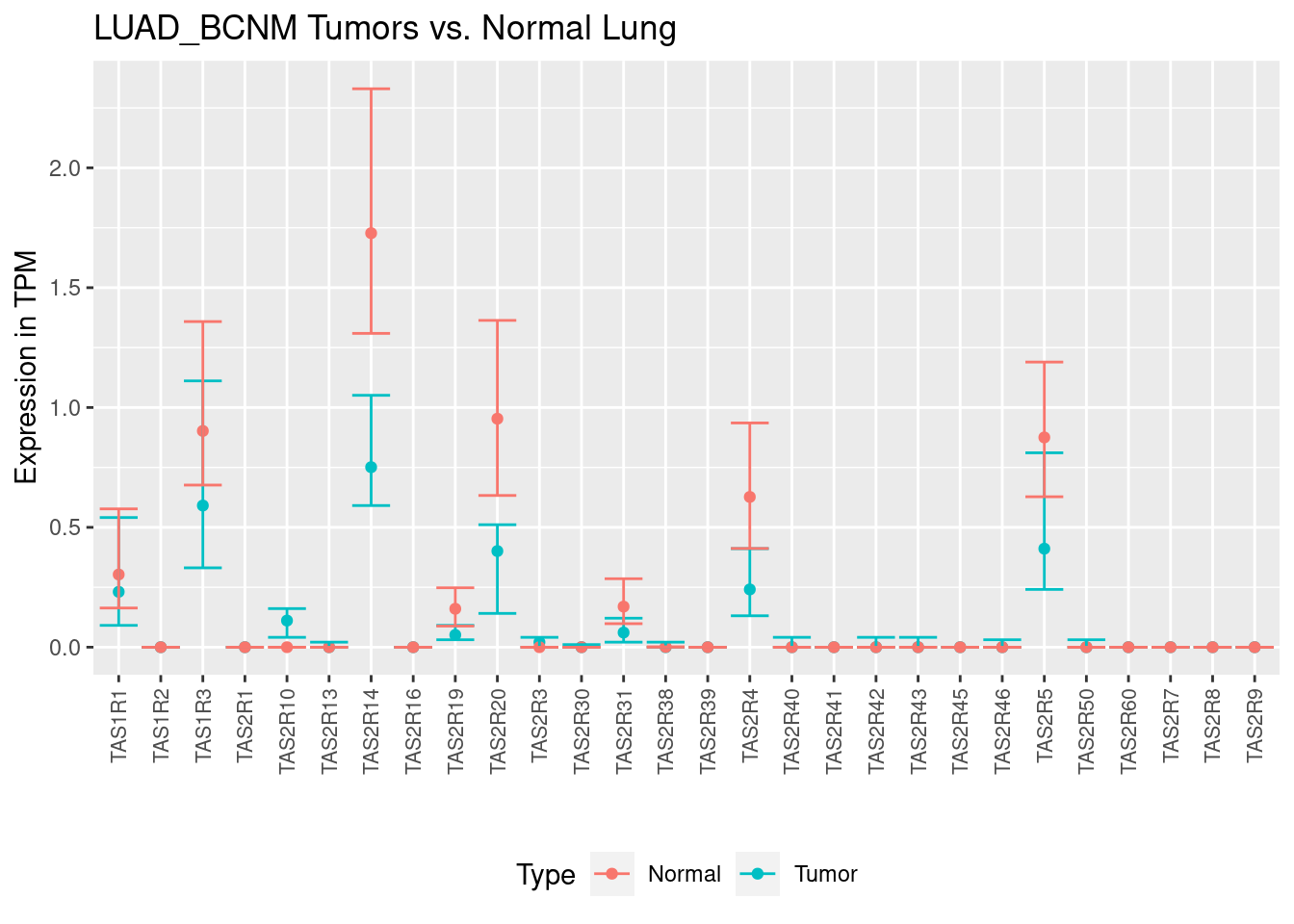

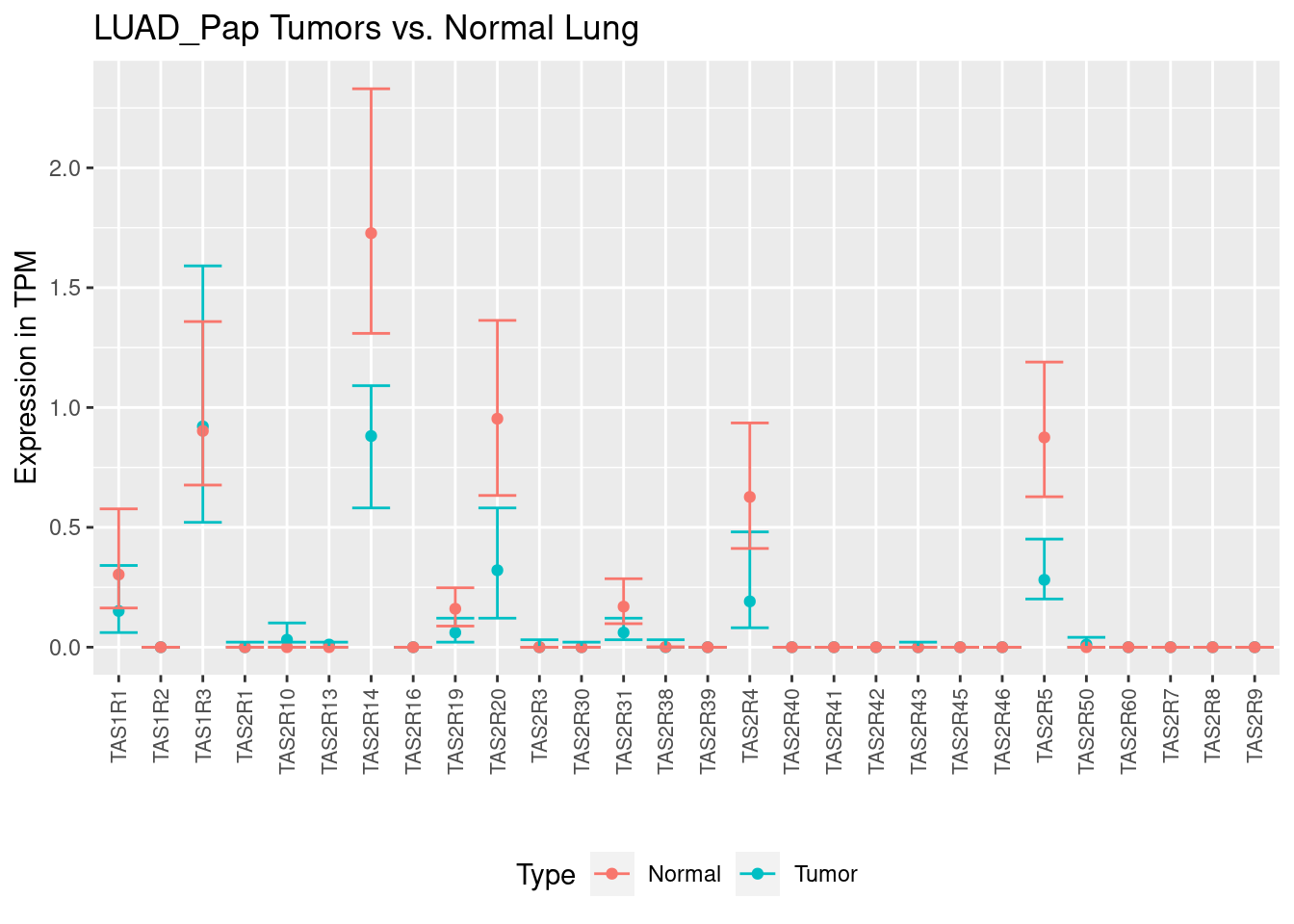

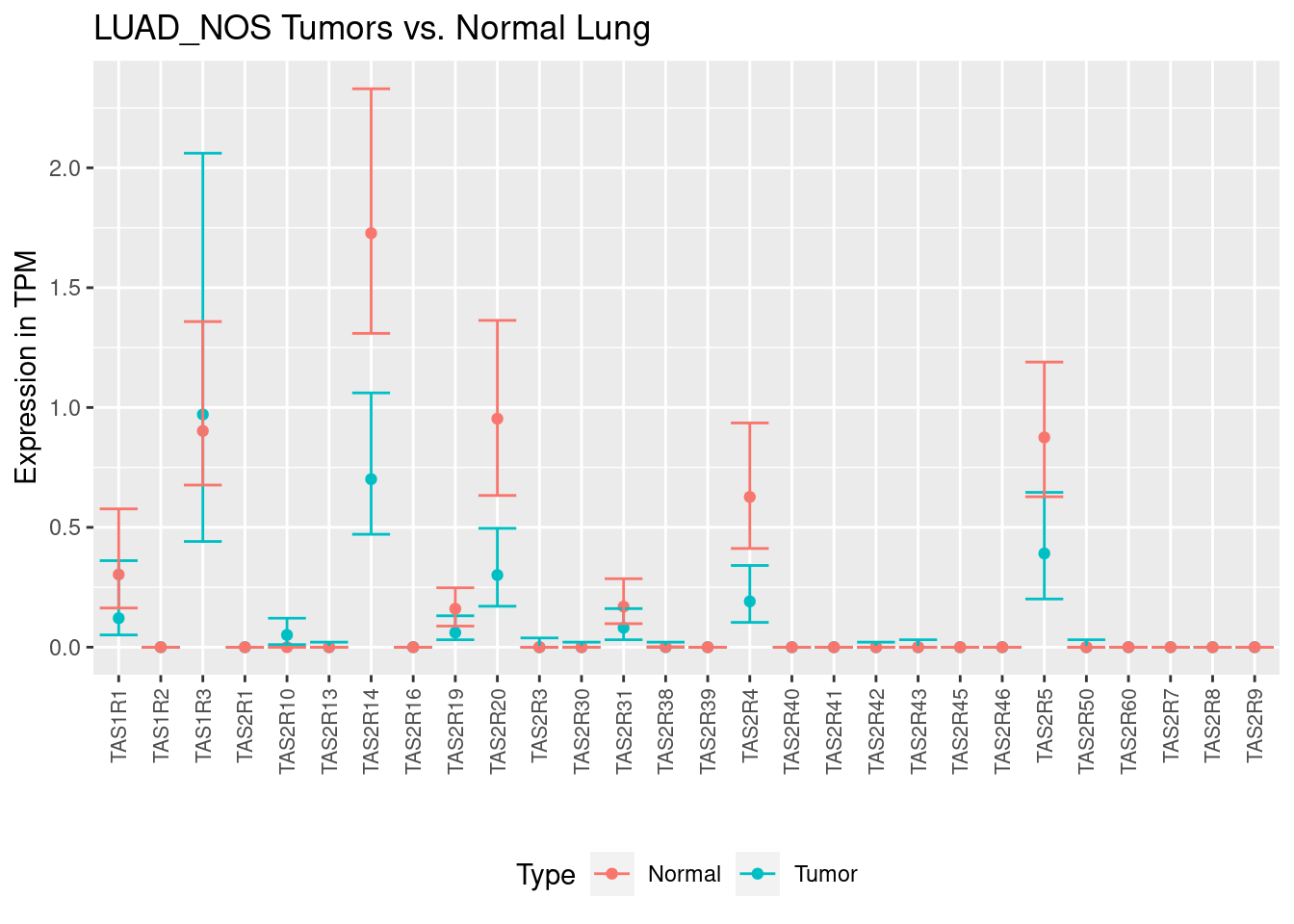

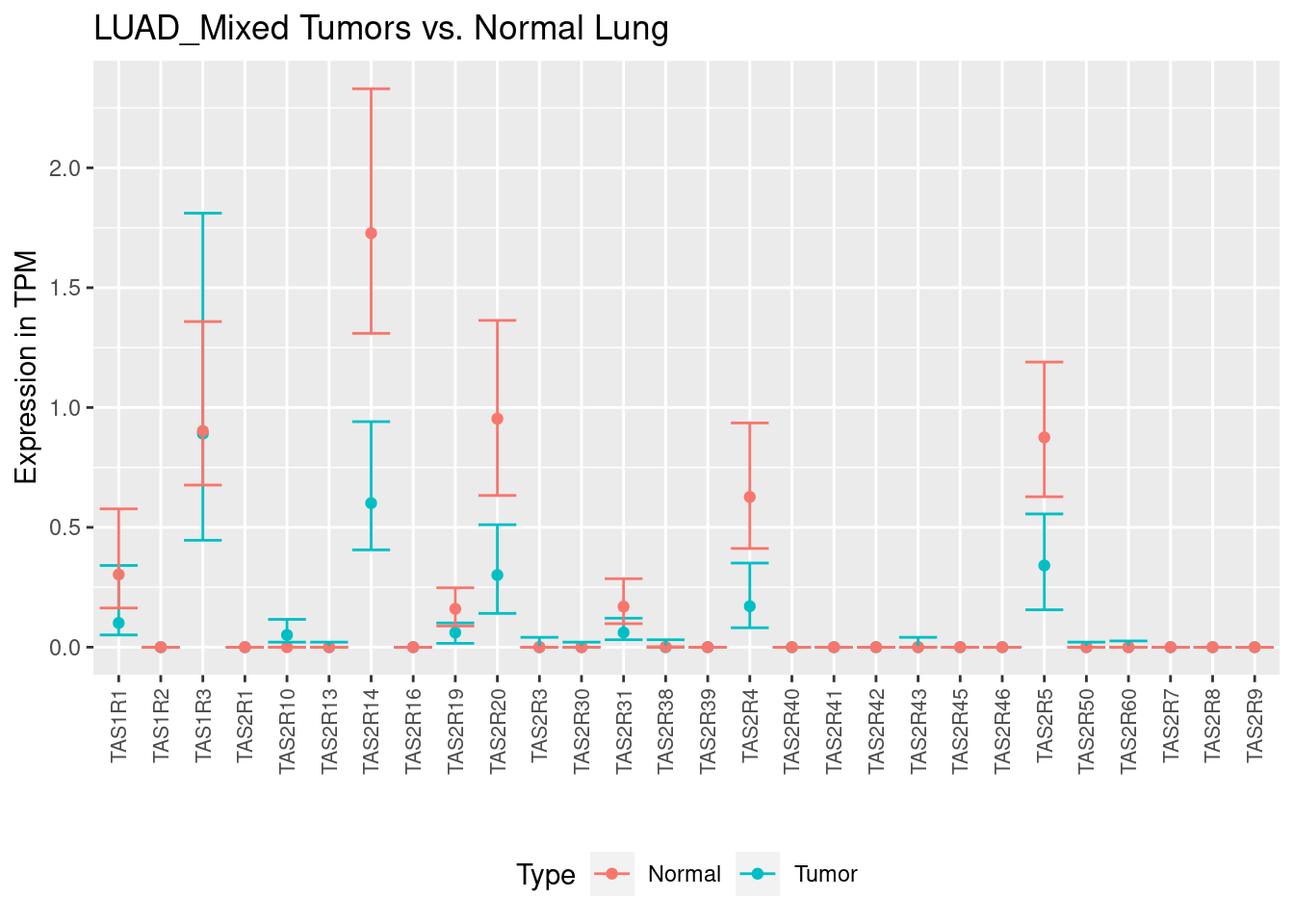

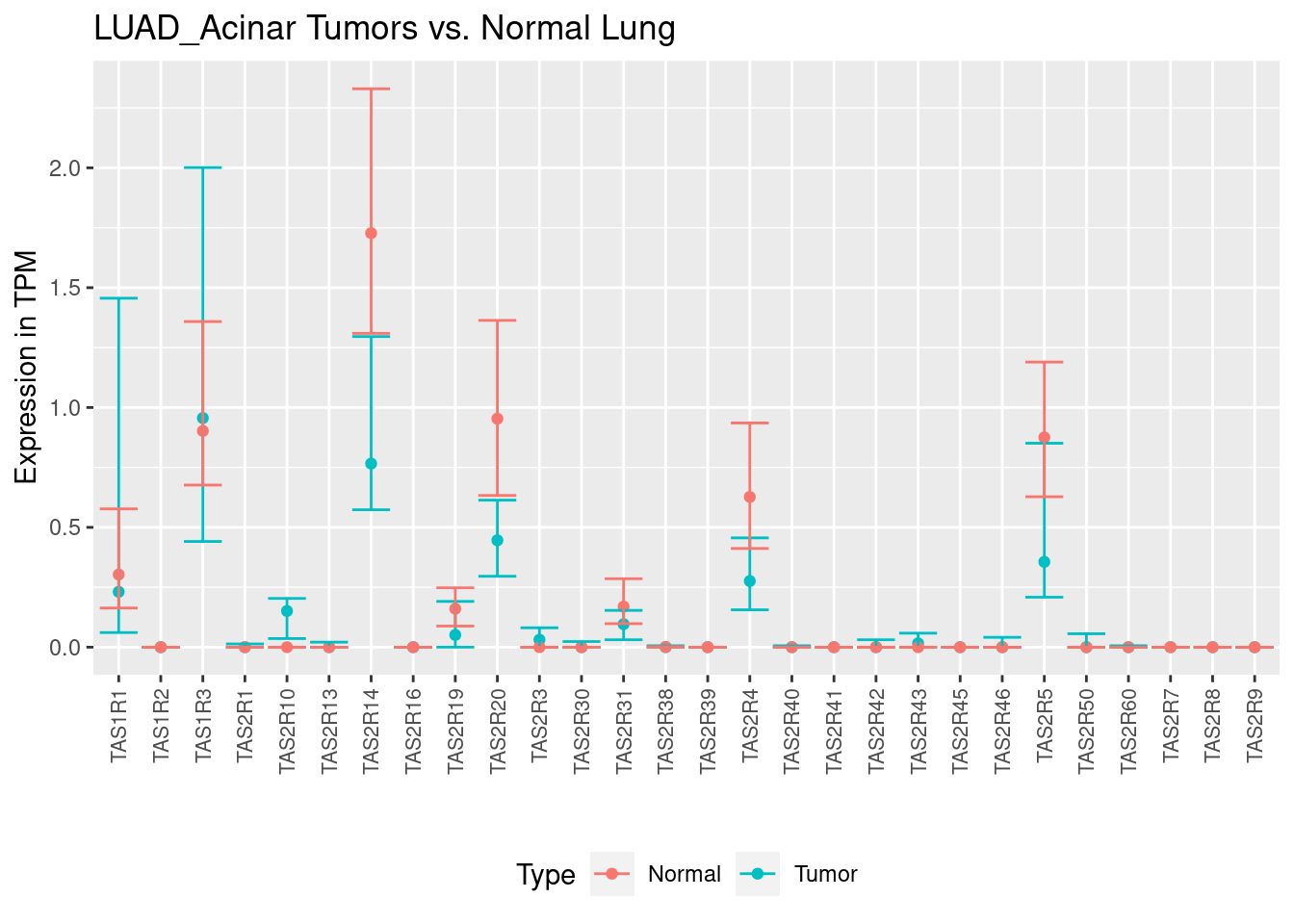

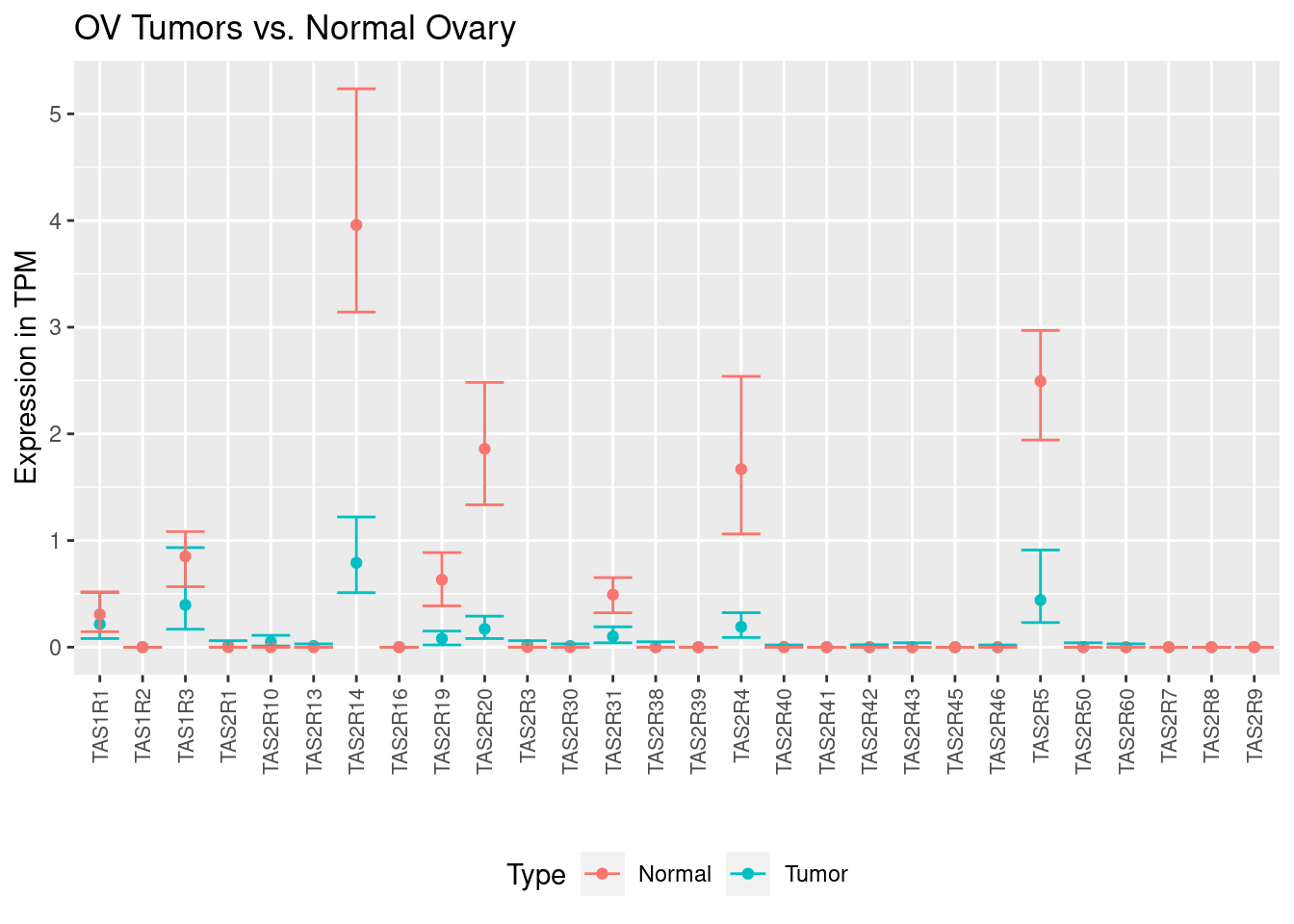

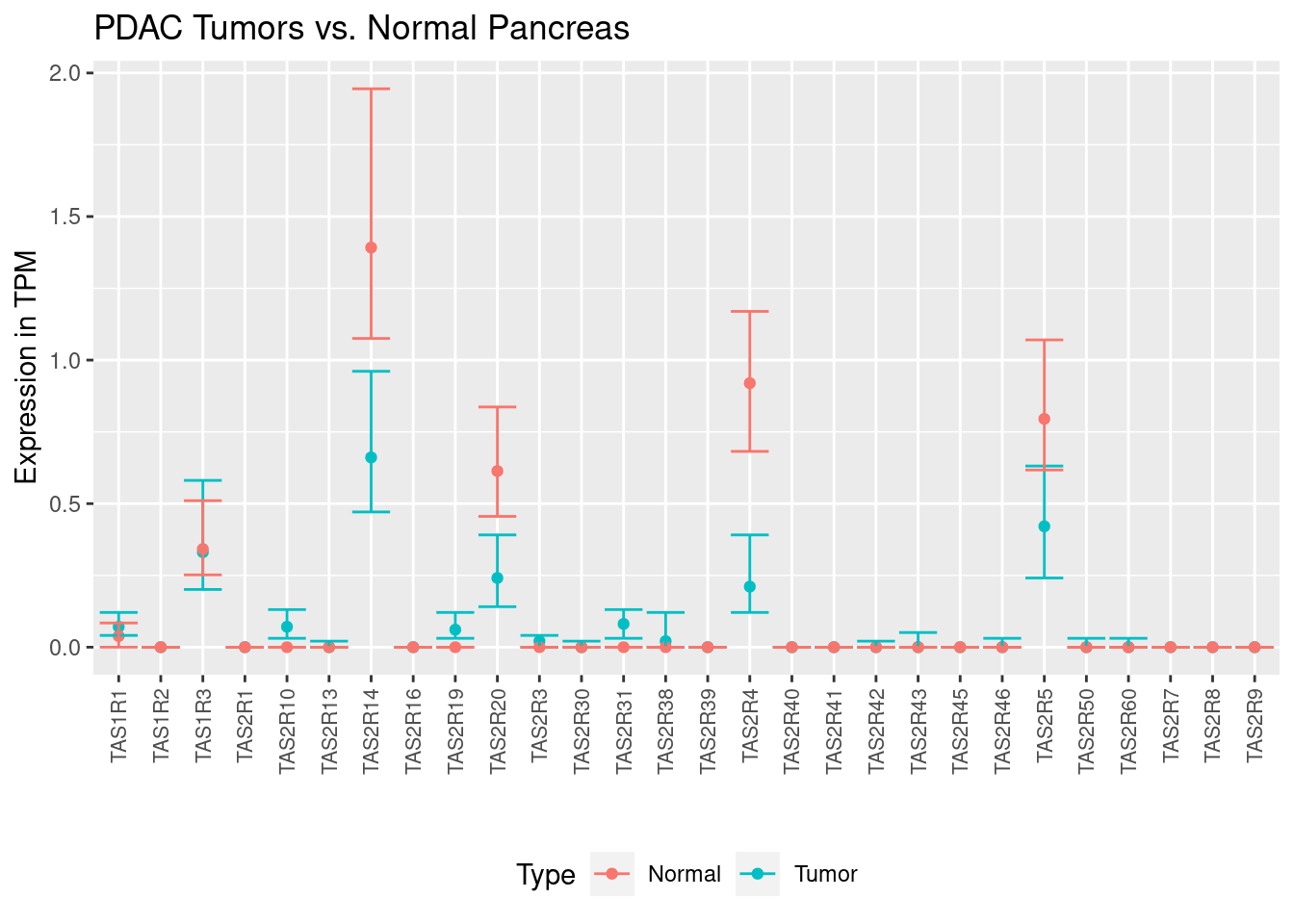

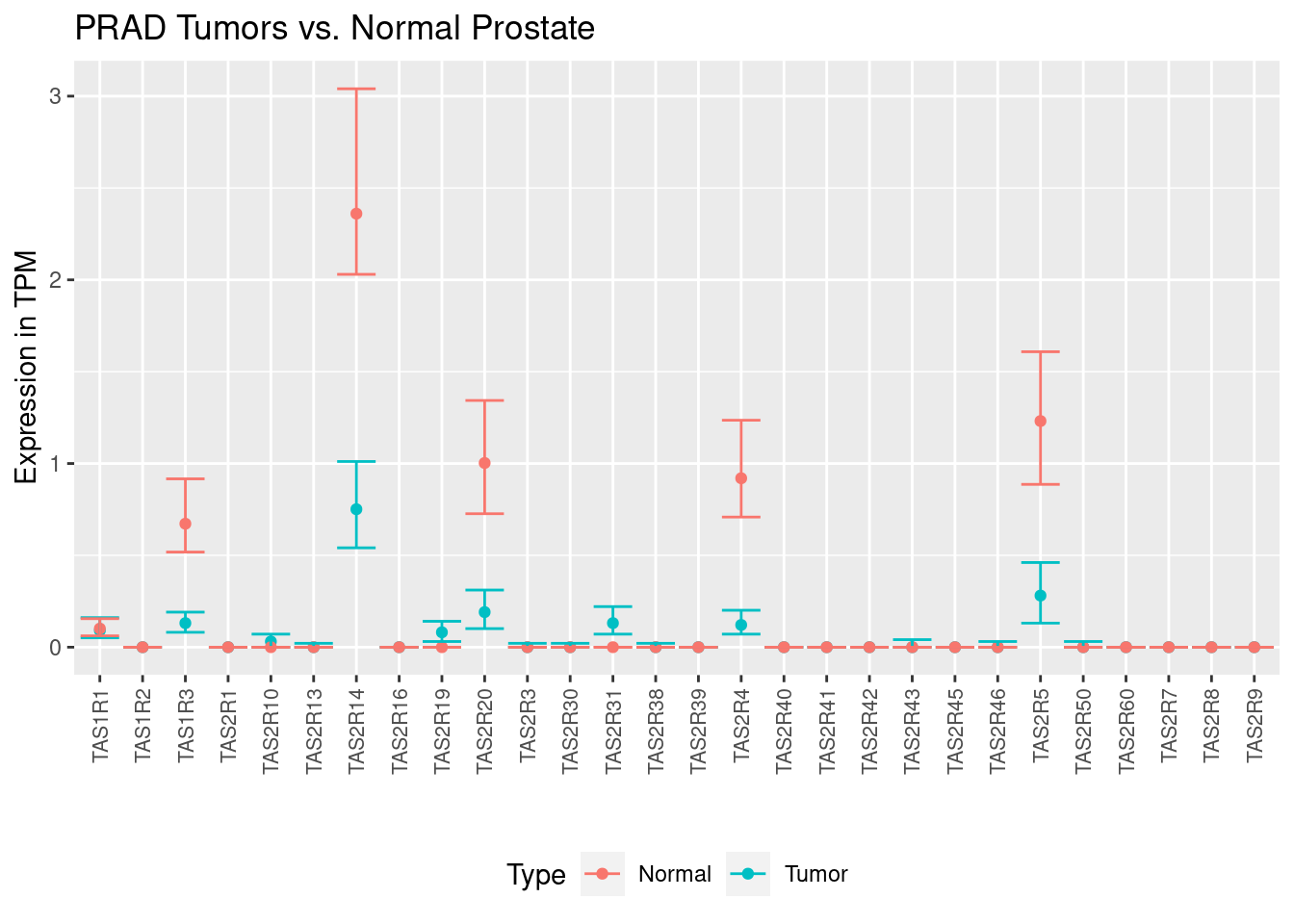

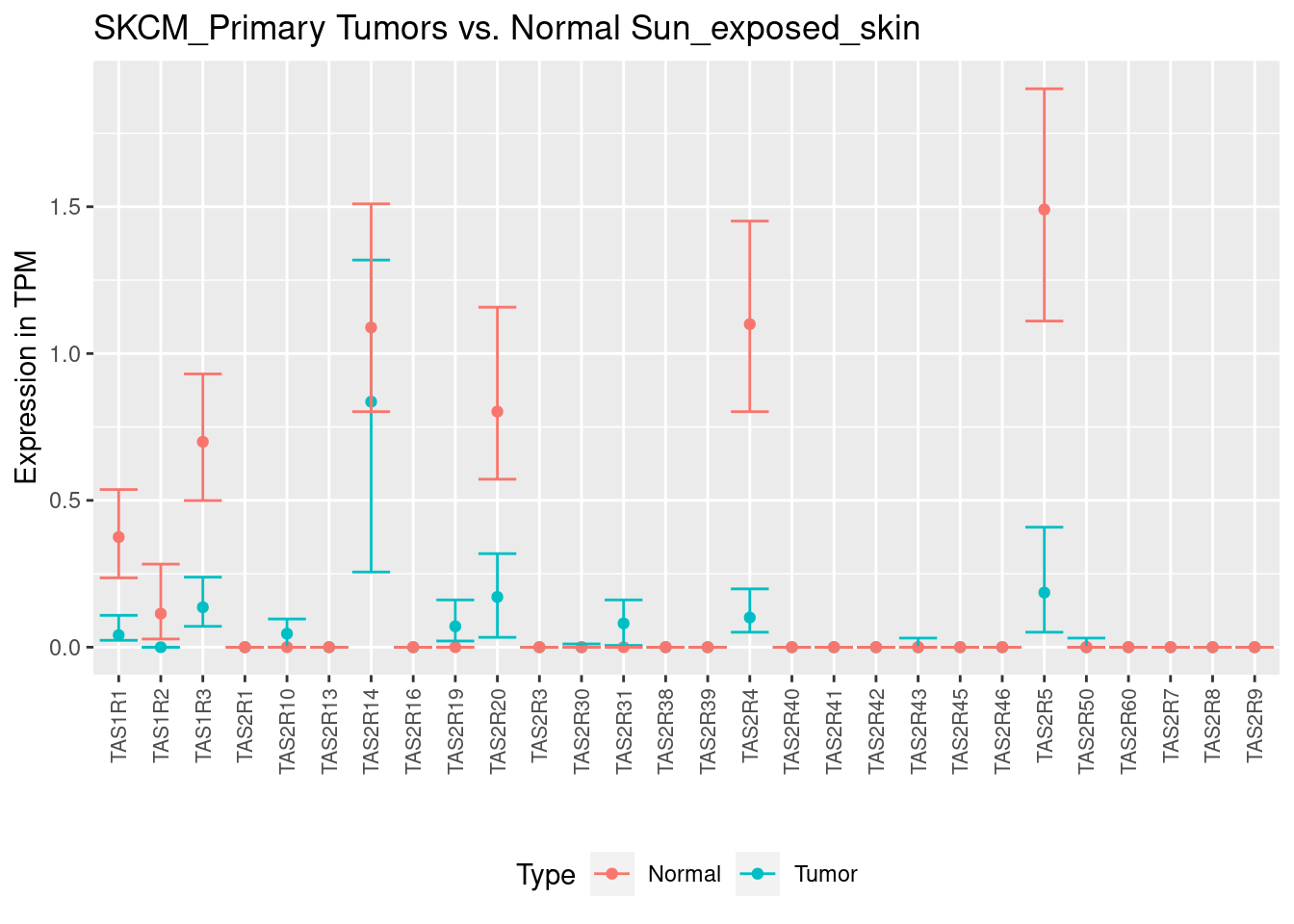

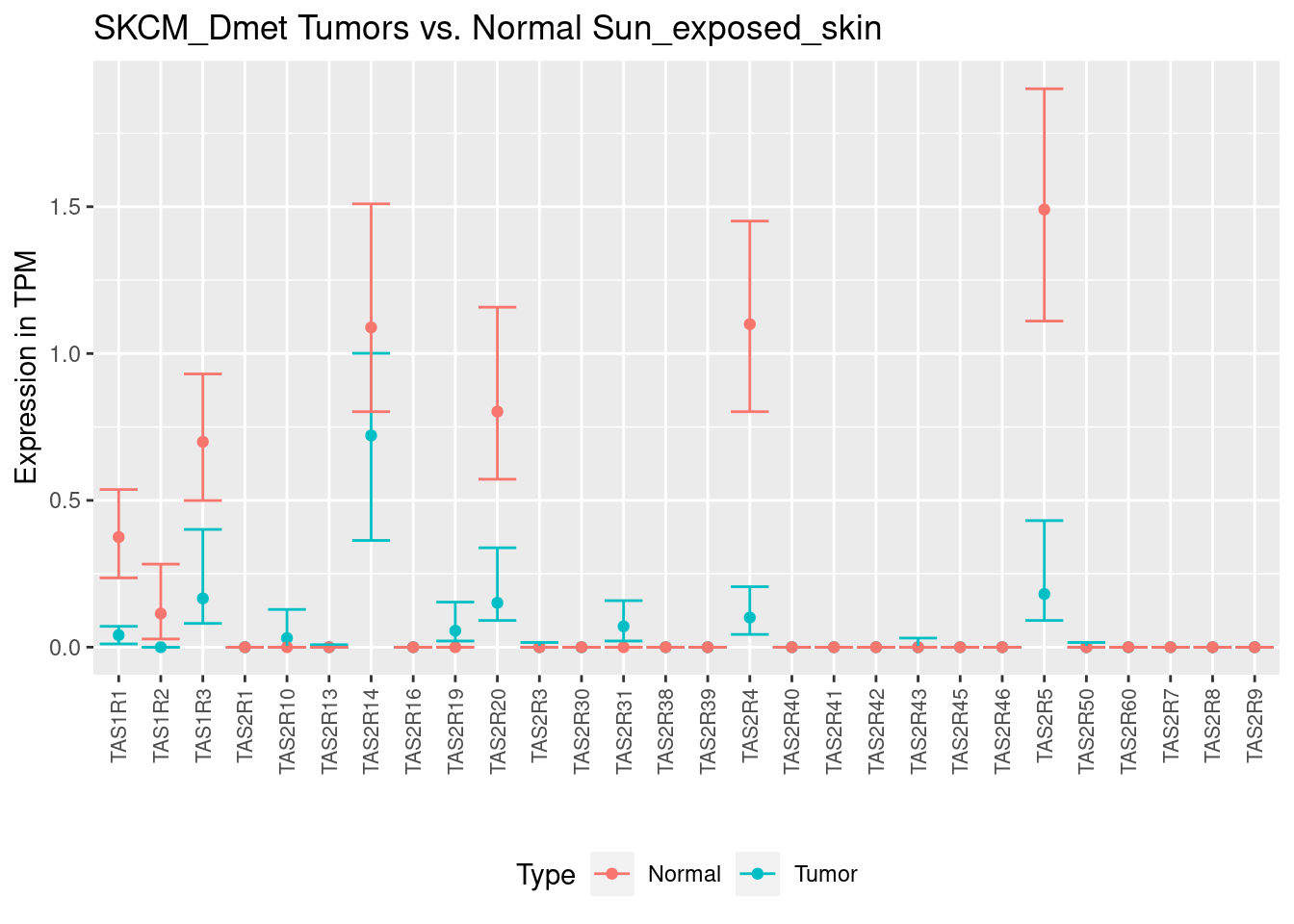

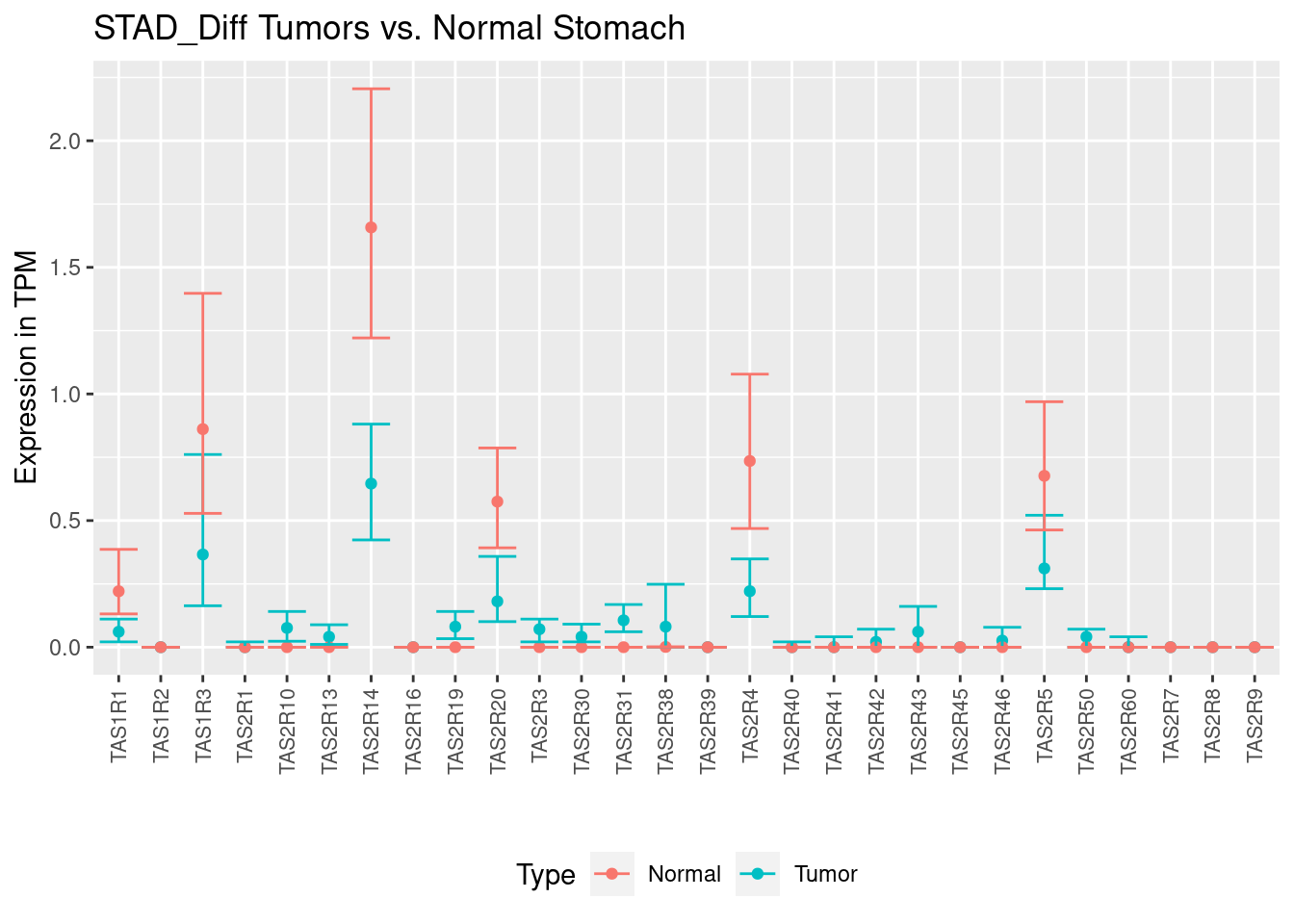

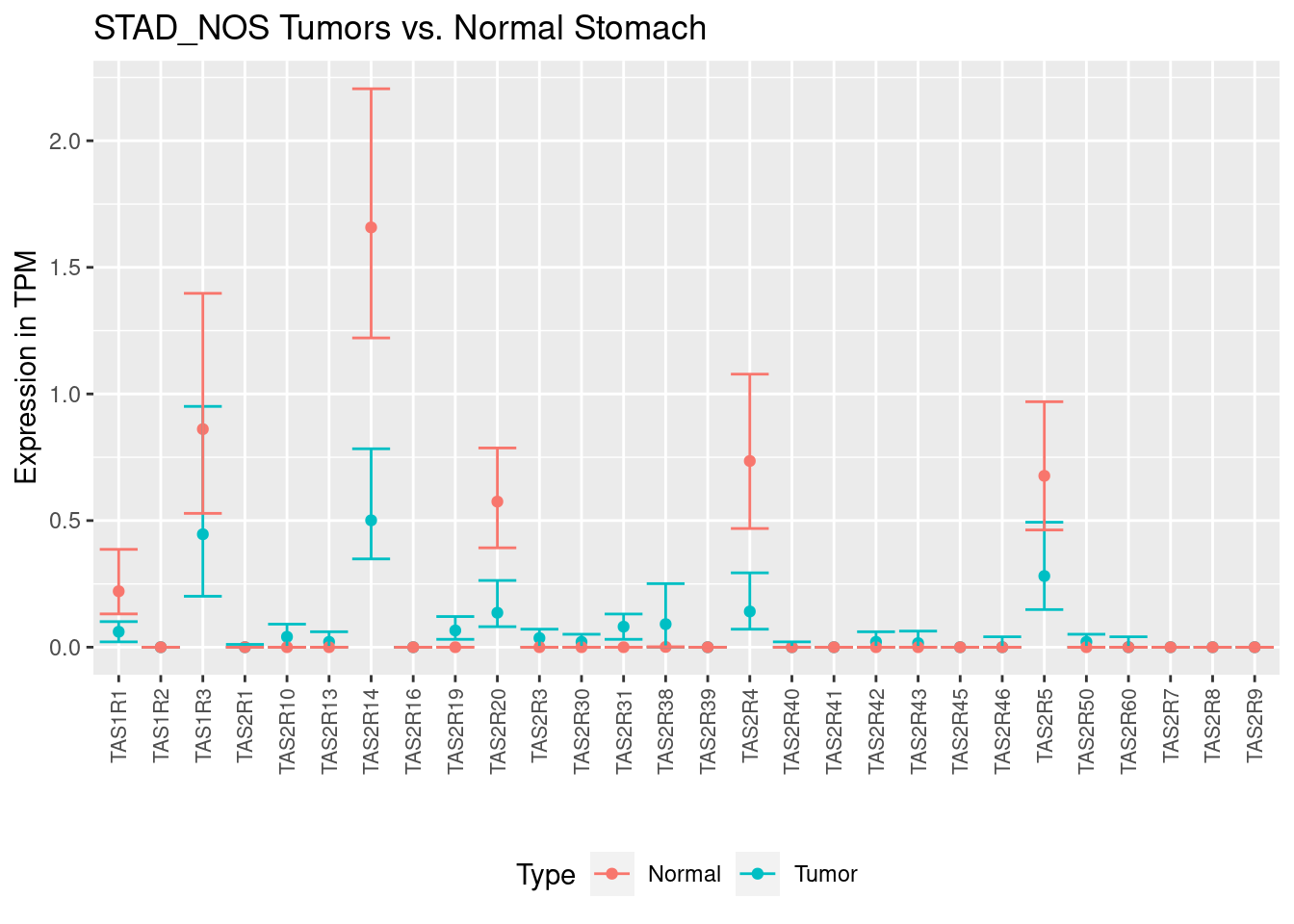

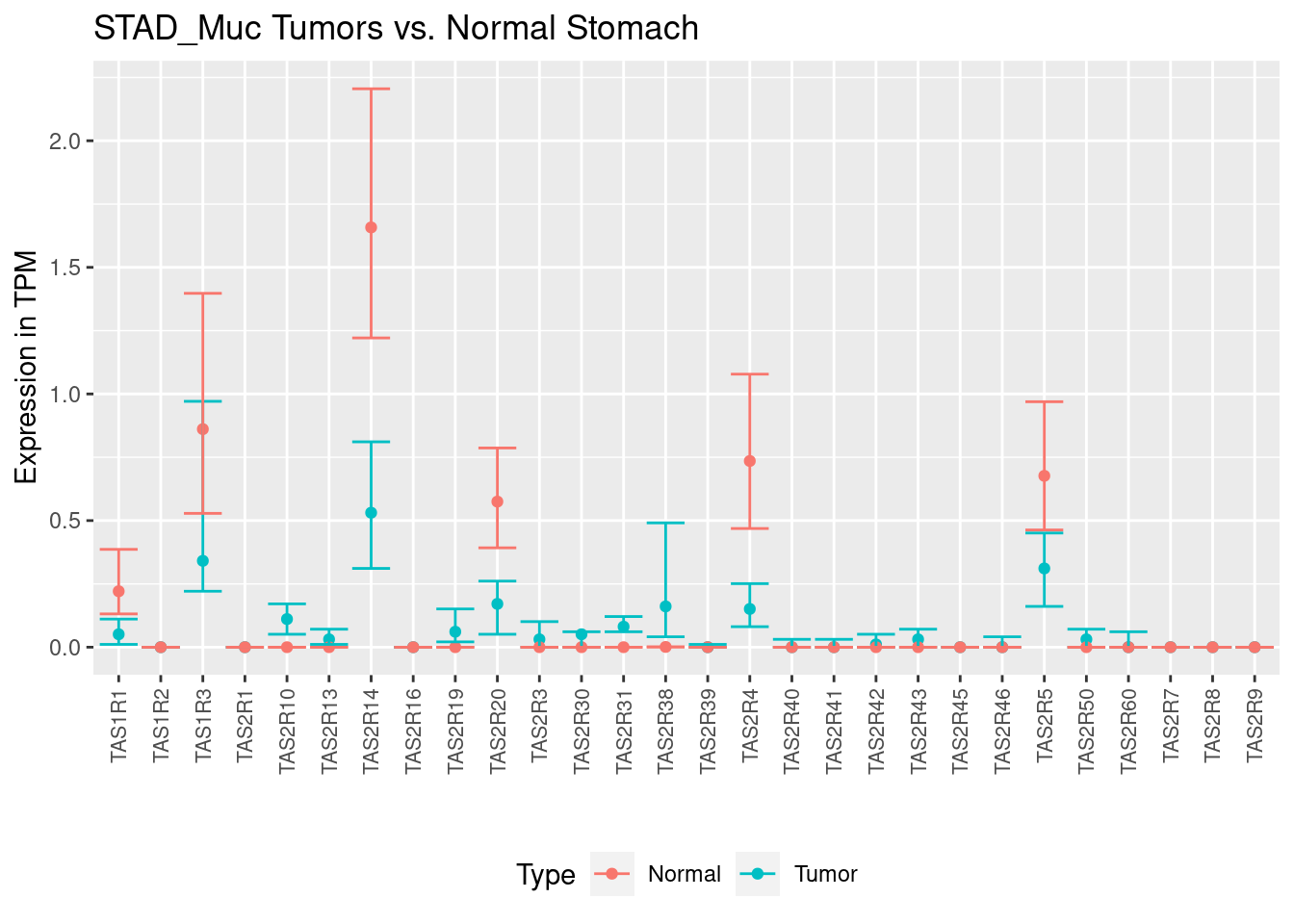

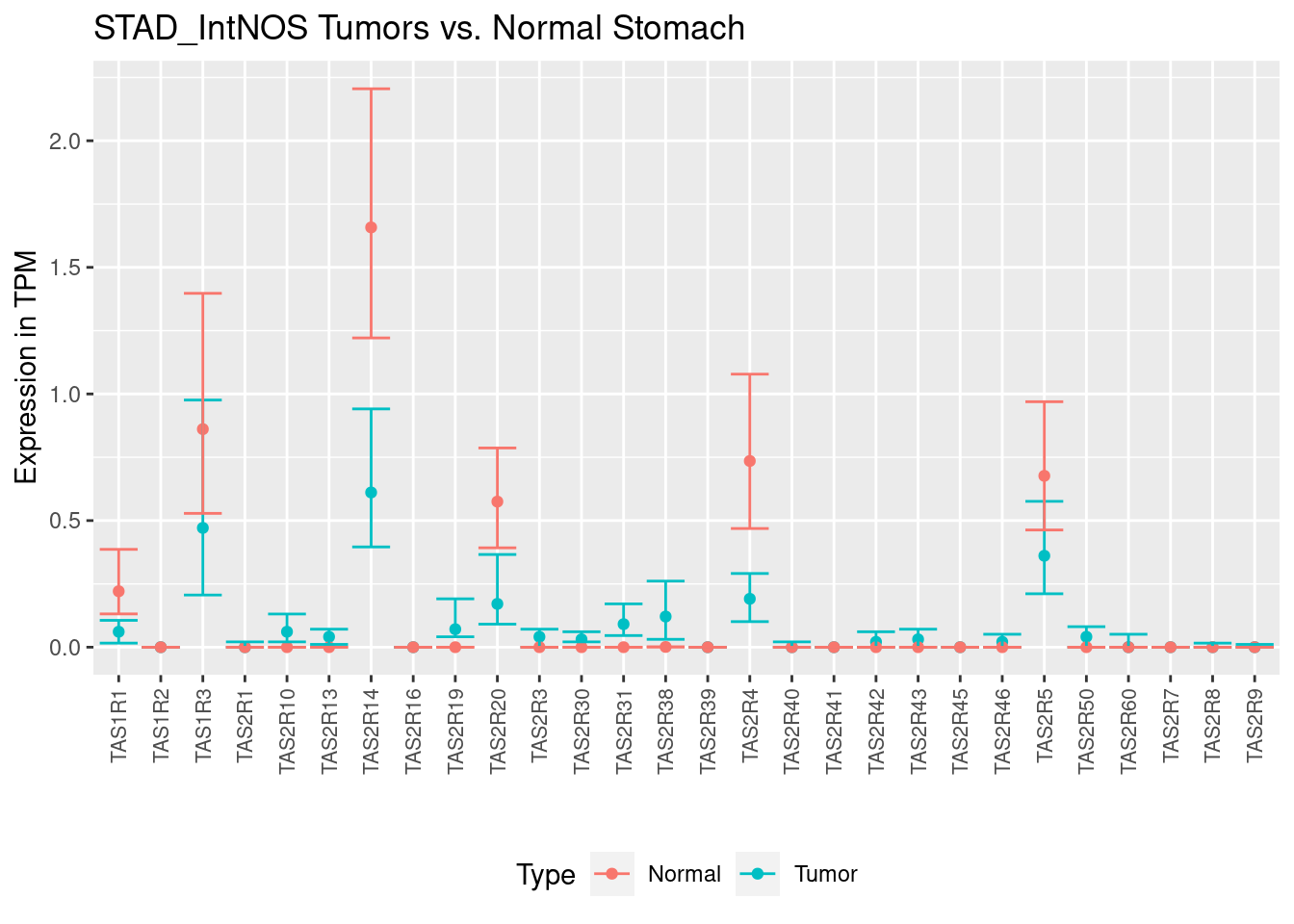

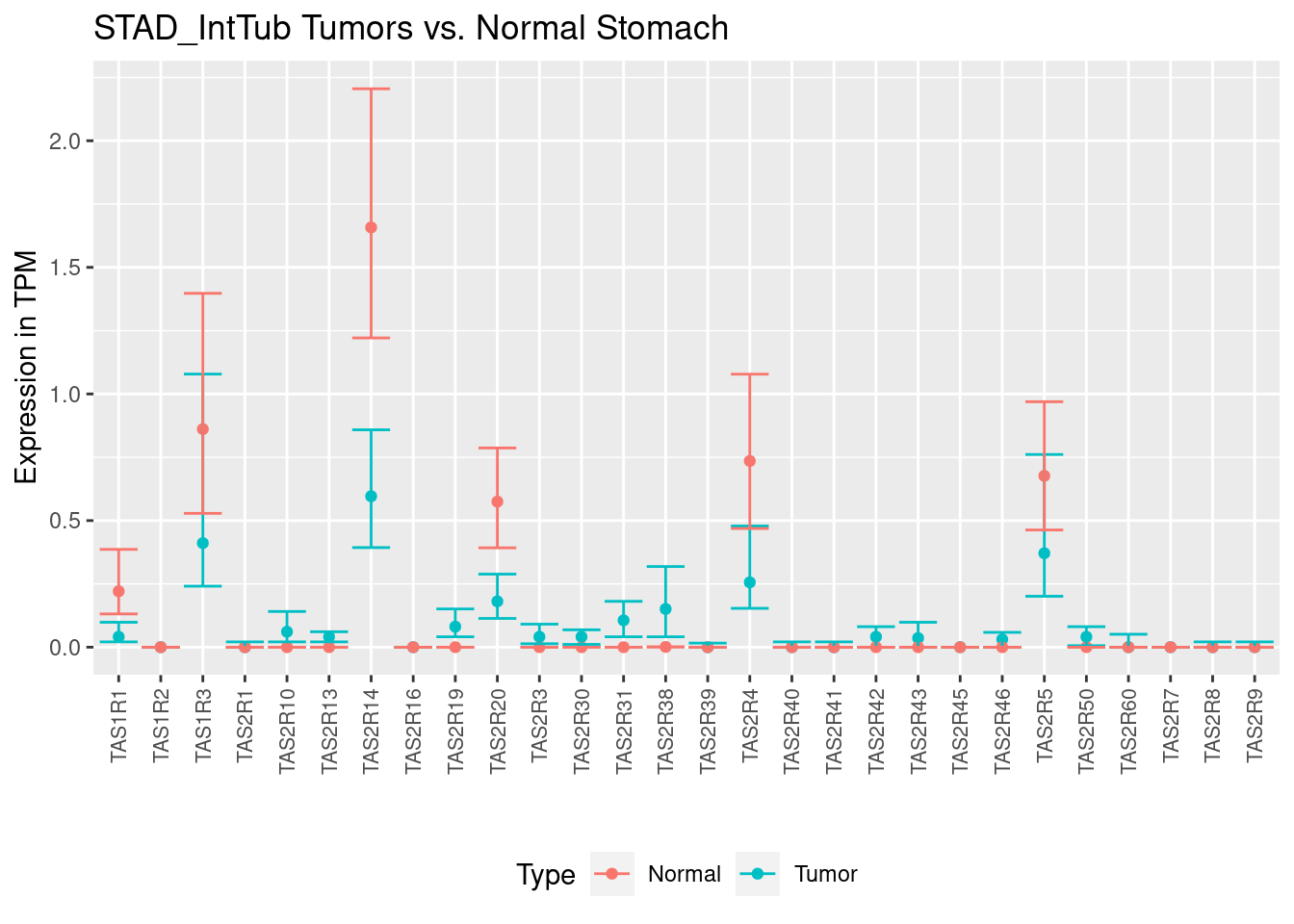

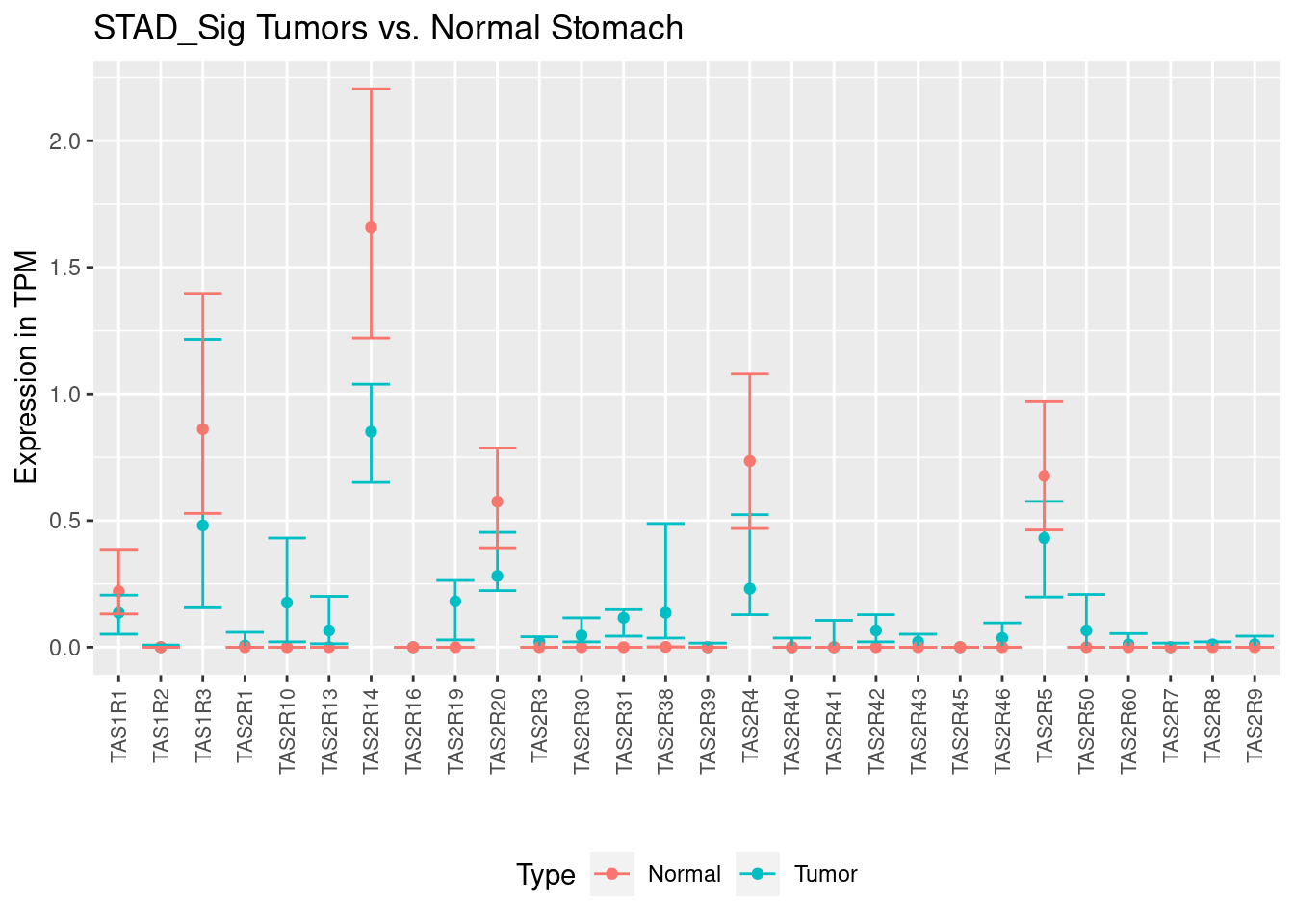

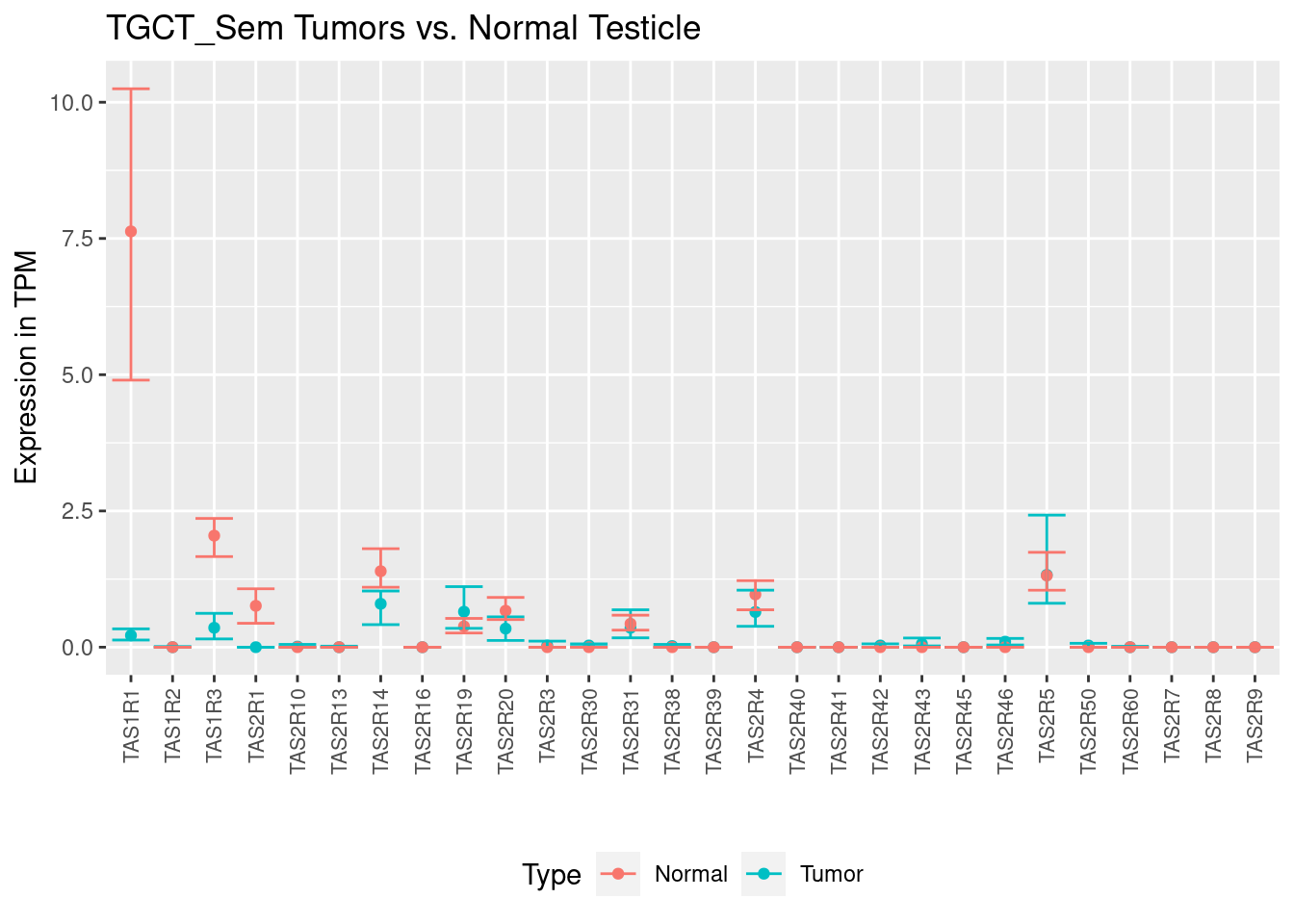

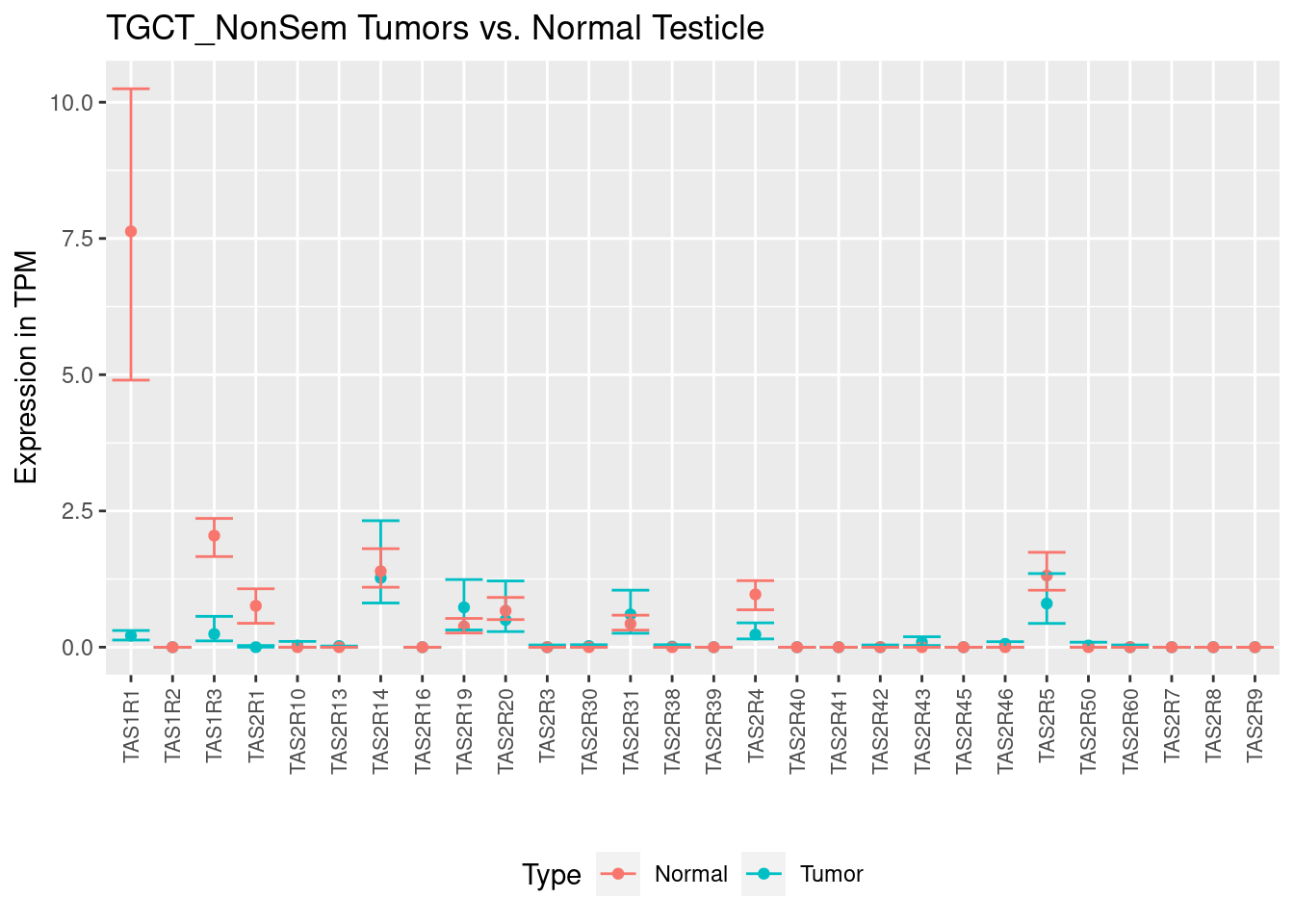

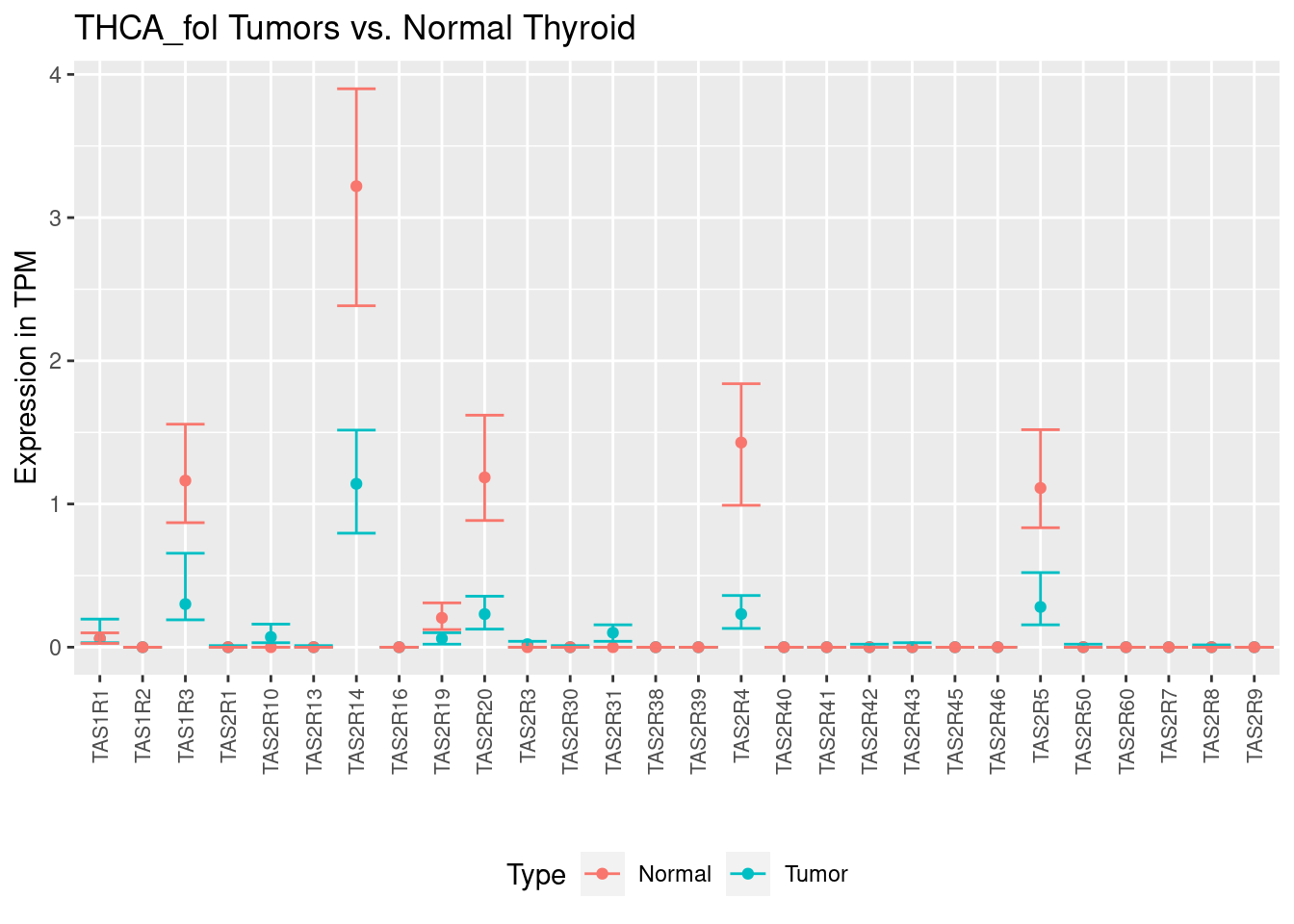

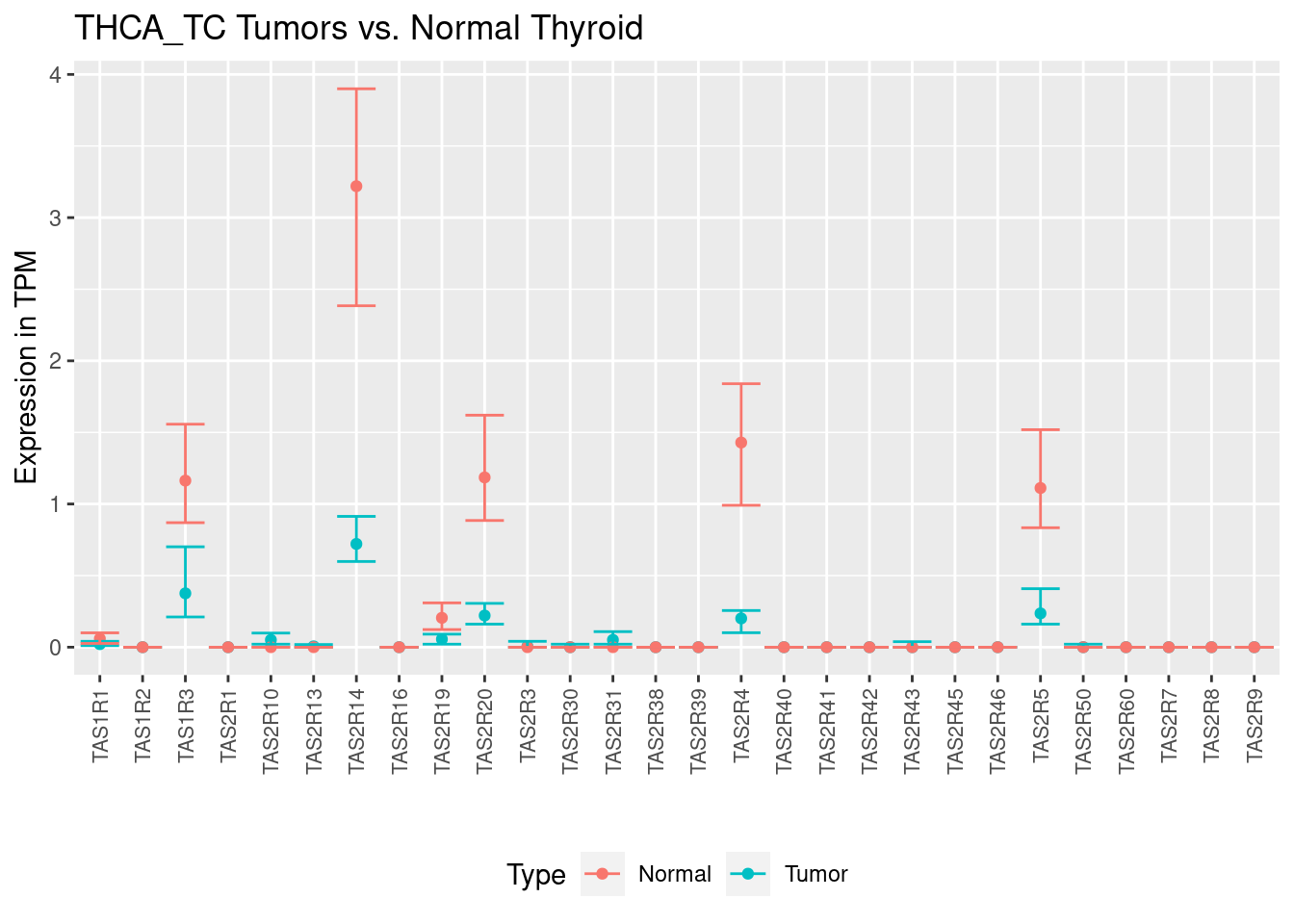

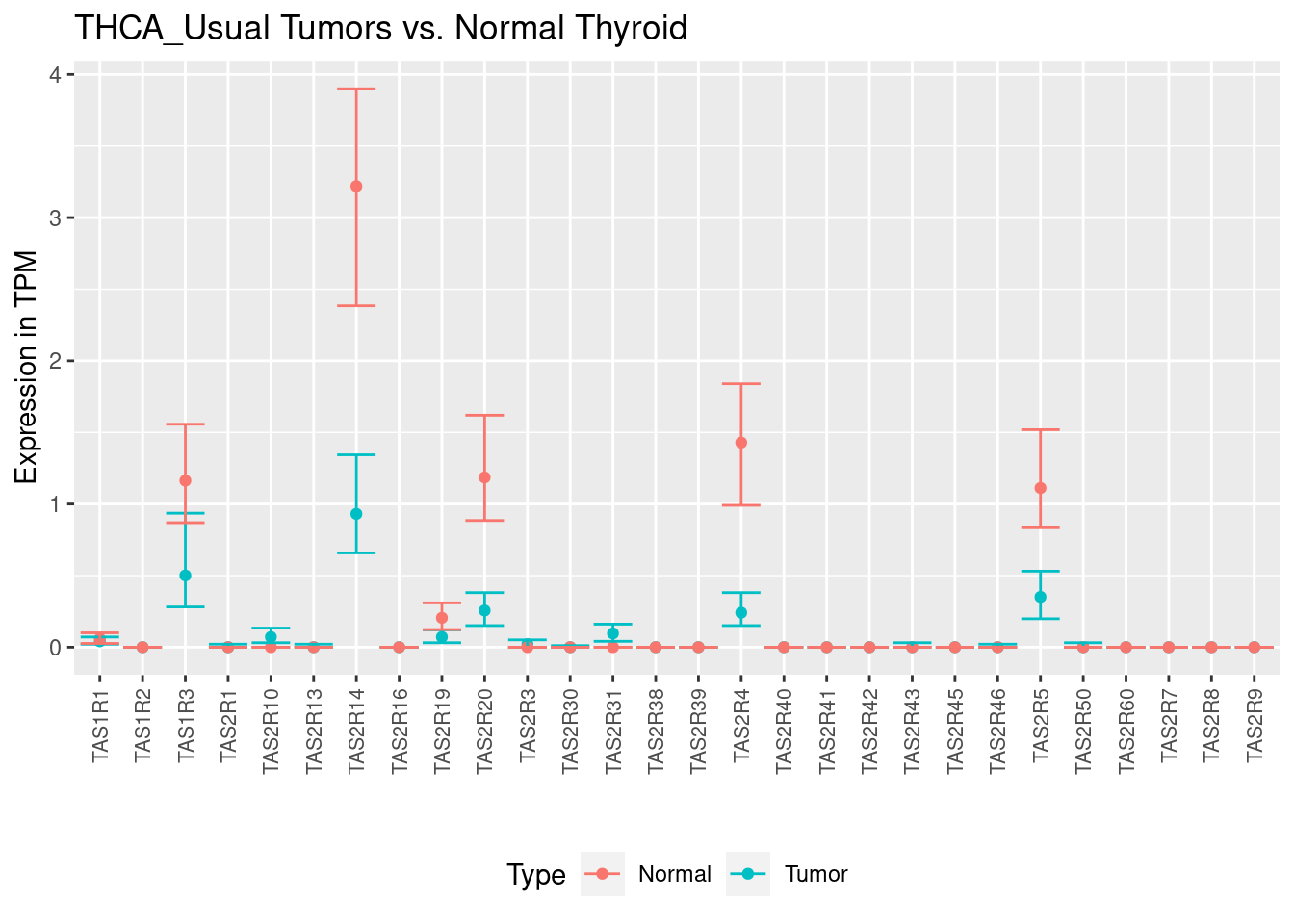

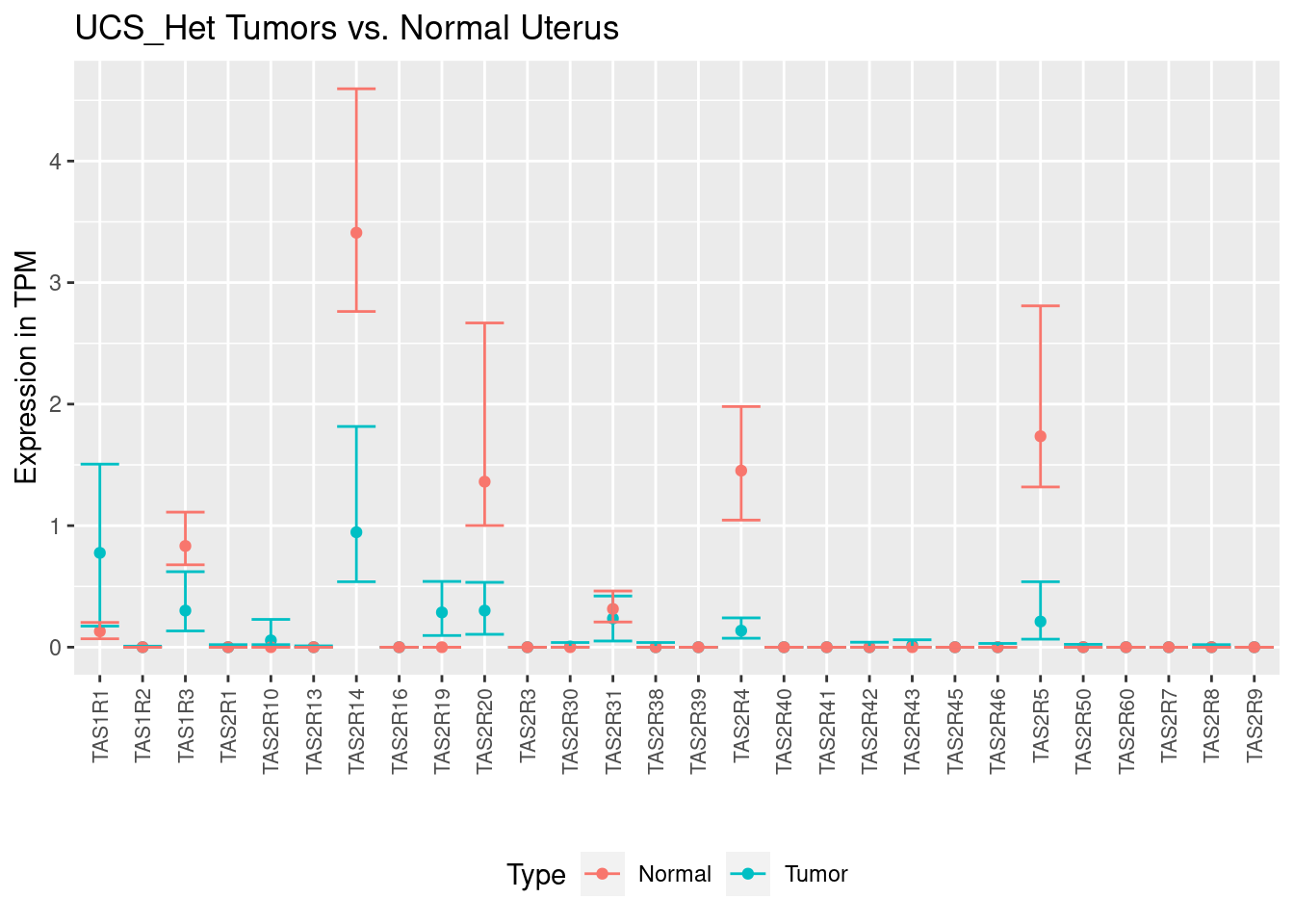

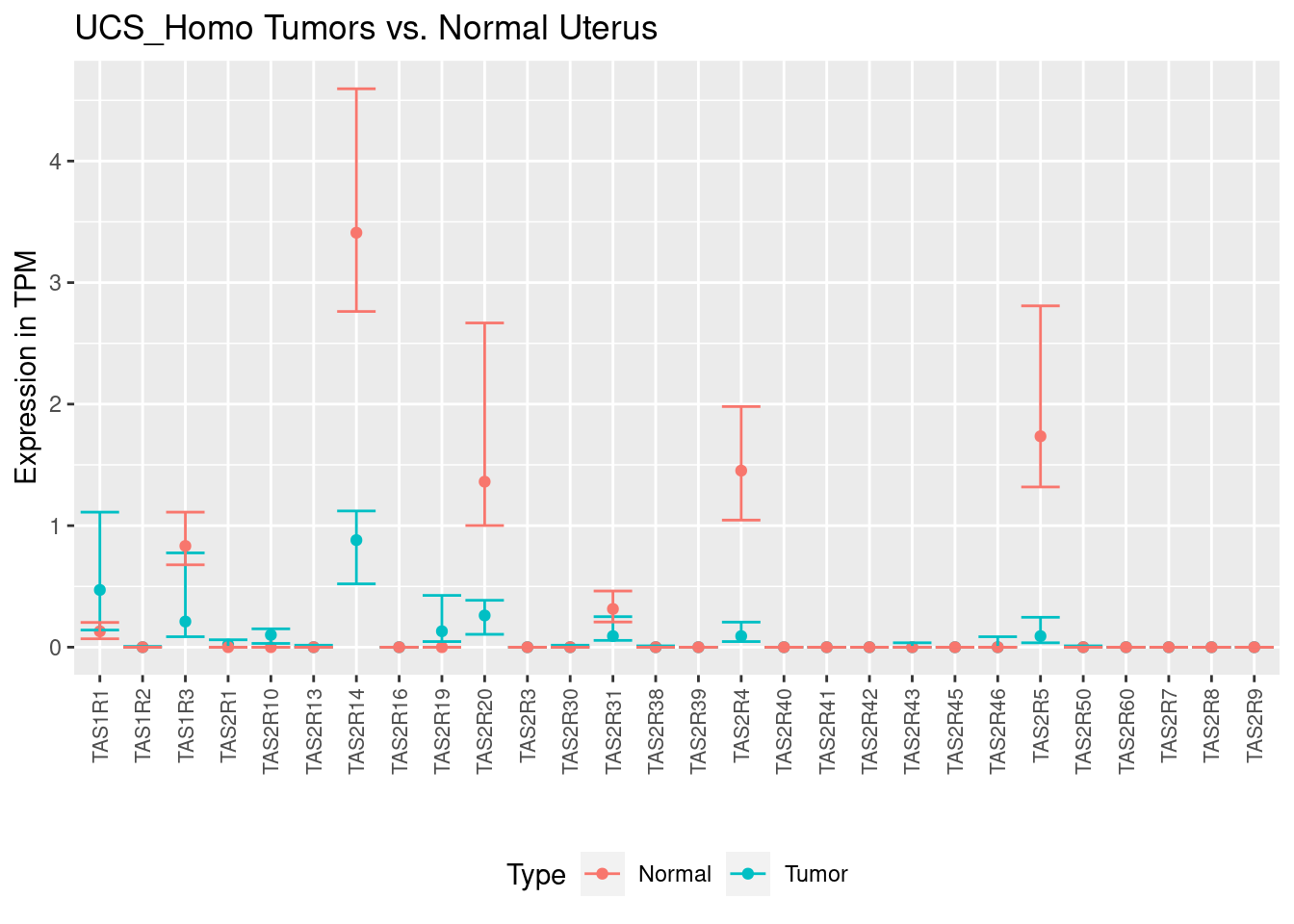

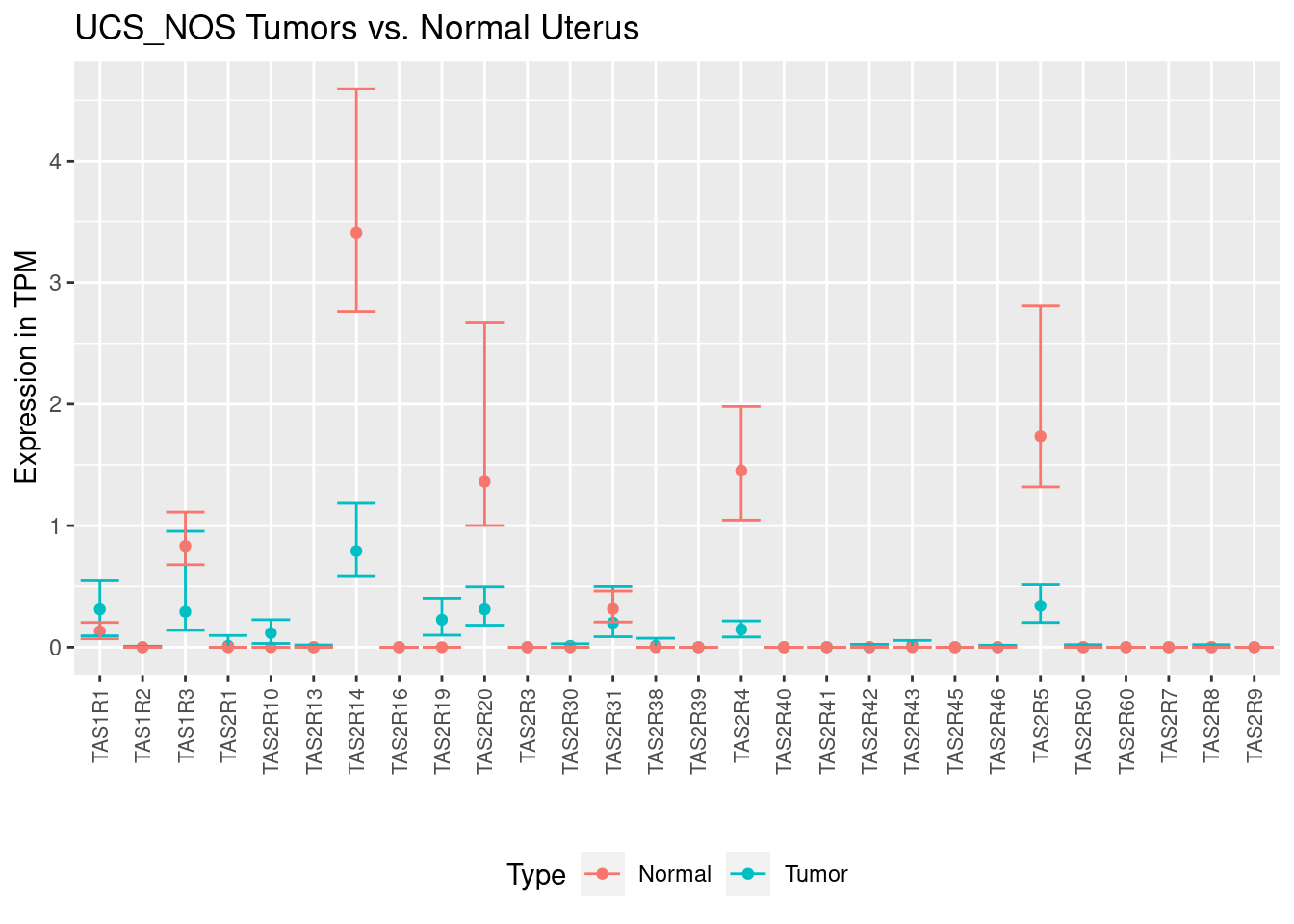


**Supplemental Figure 2.** *TAS1R* and *TAS2R* expression data for solid tumors (blue) and corresponding normal tissue (red). Data is displayed as median transcripts per million (TPM) with upper and lower quartiles of expression. TPM, Transcripts Per Million. Plots generated using files from <https://insellab.github.io/>^47^ and labeled using The Cancer Genome Atlas (TCGA) cancer type and subclassification as defined in Figure 1A.


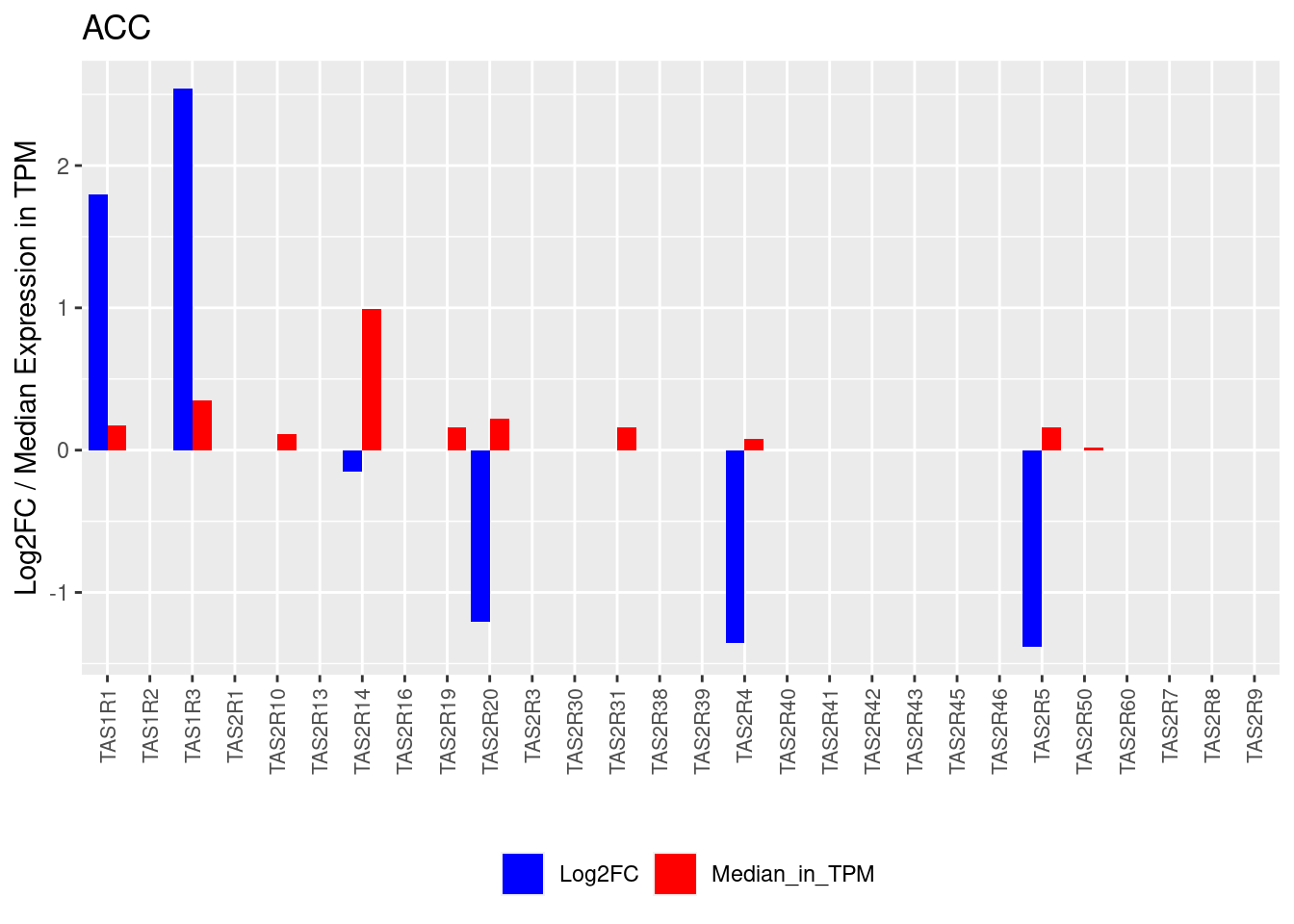

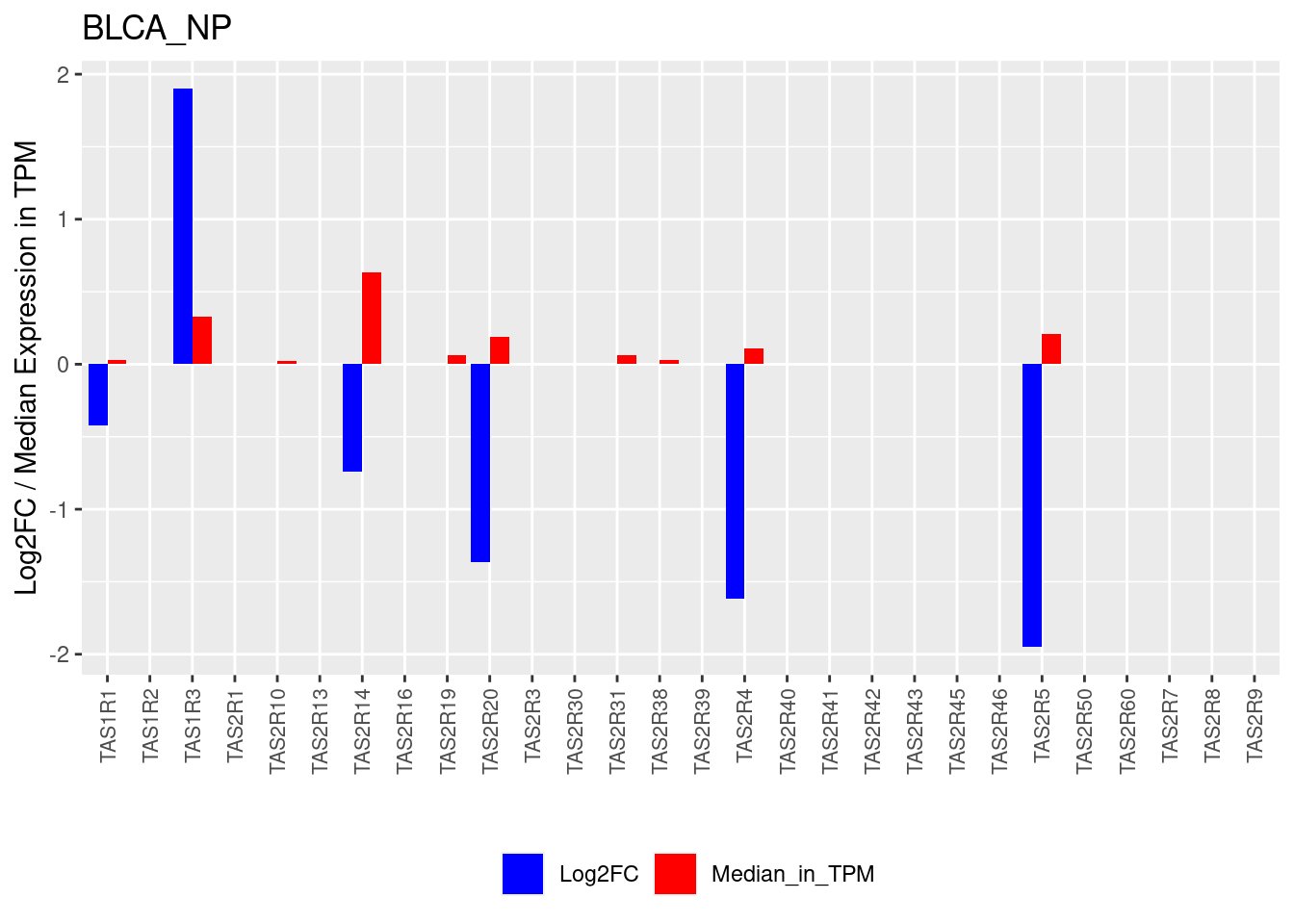

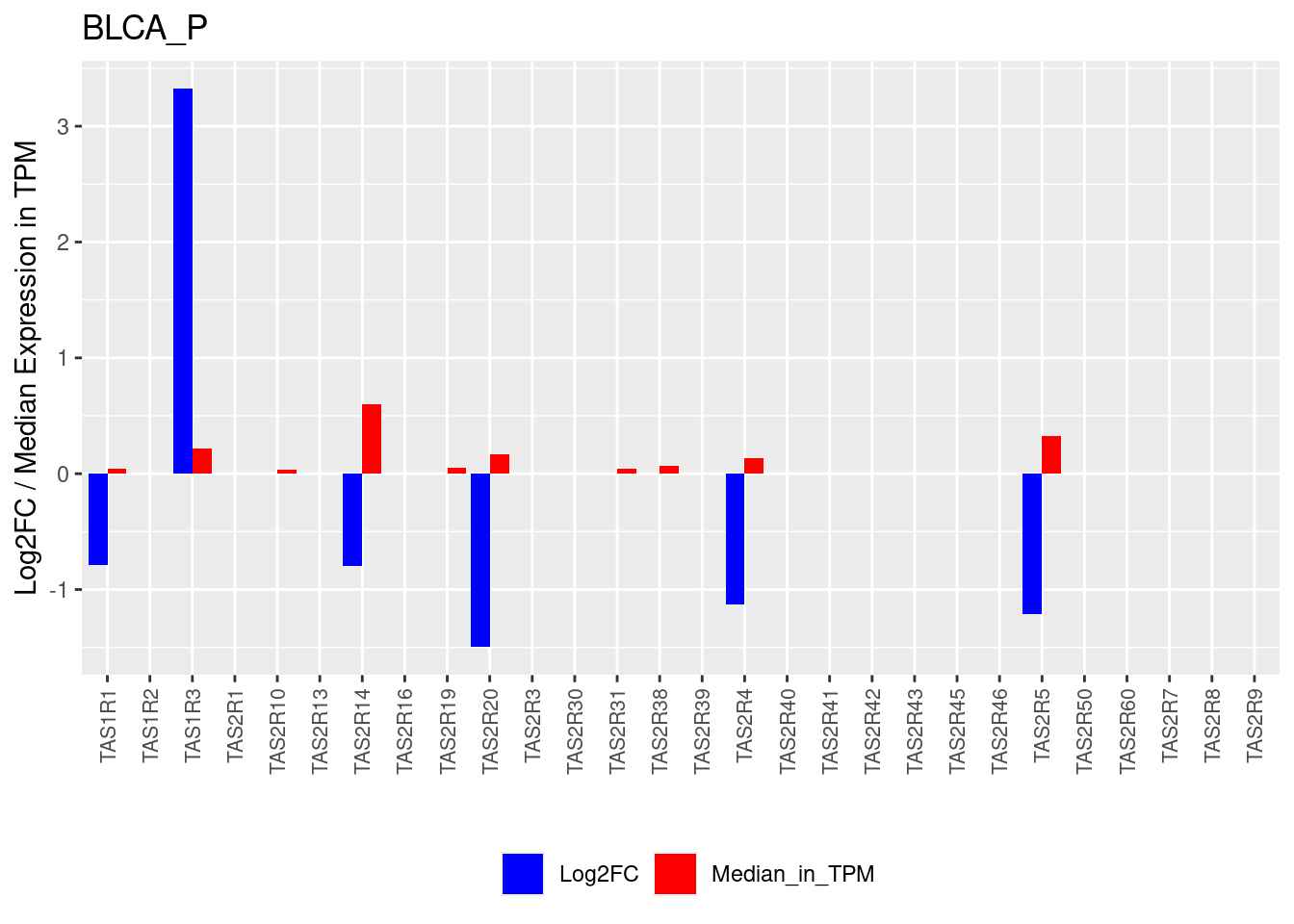

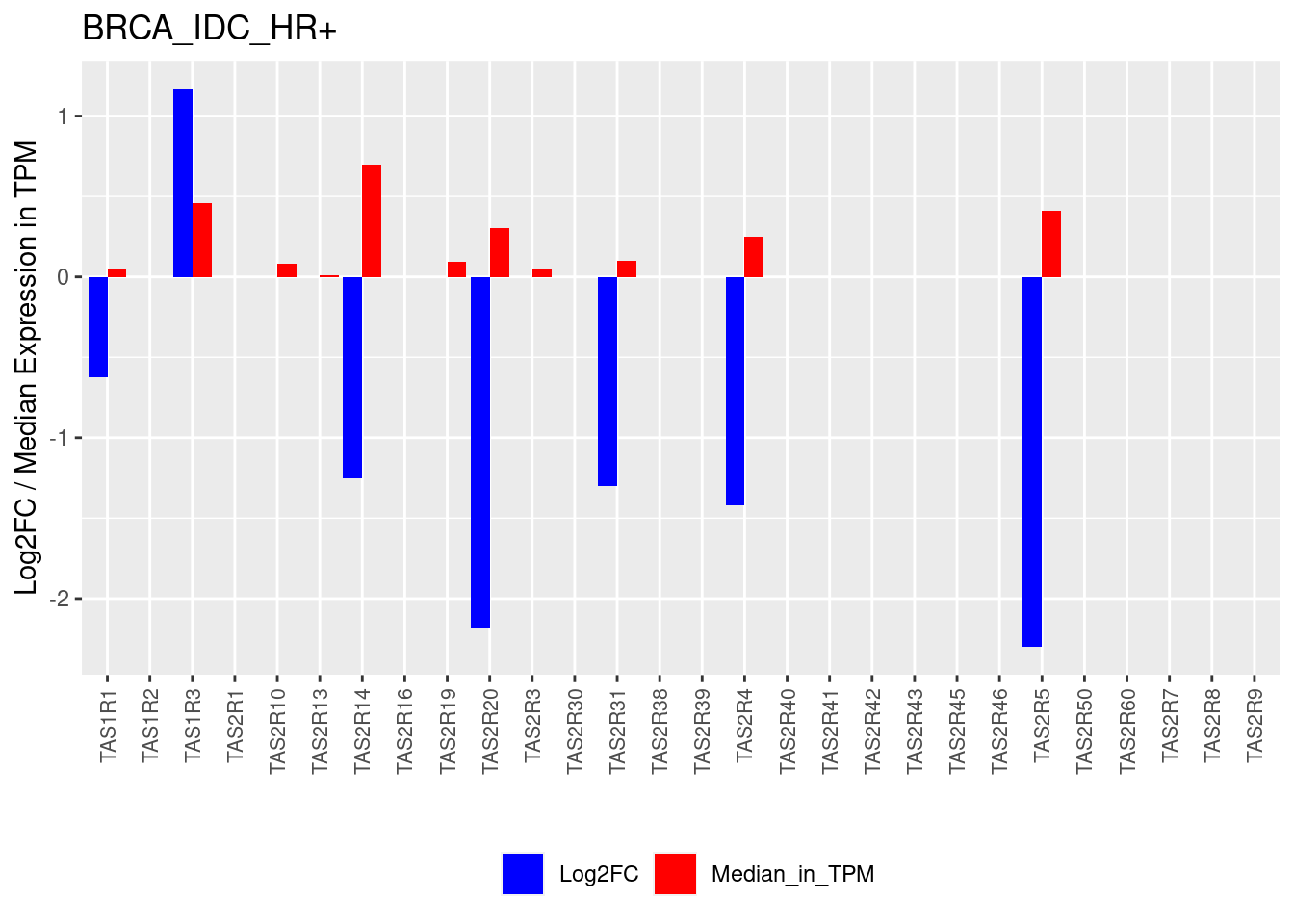

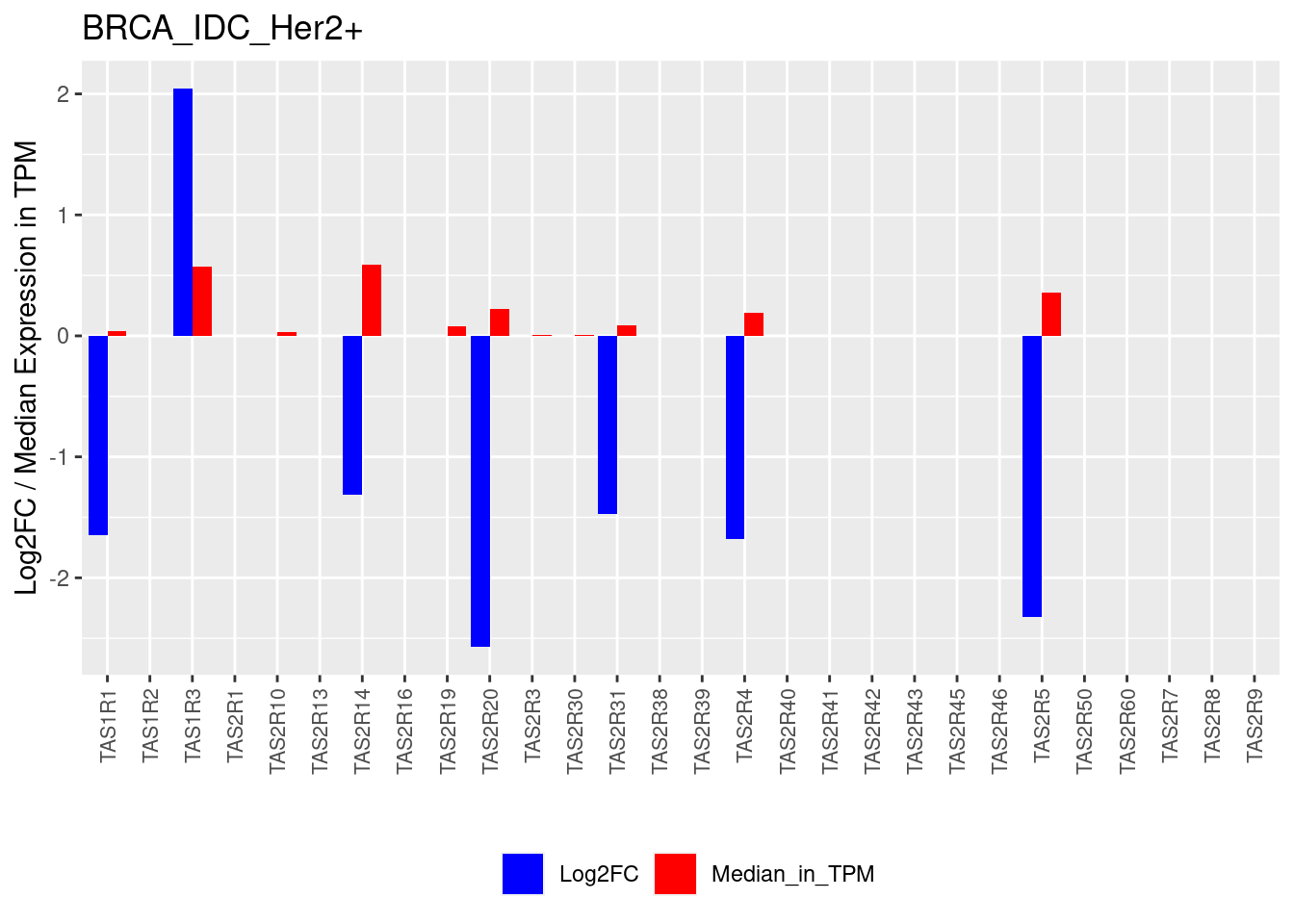

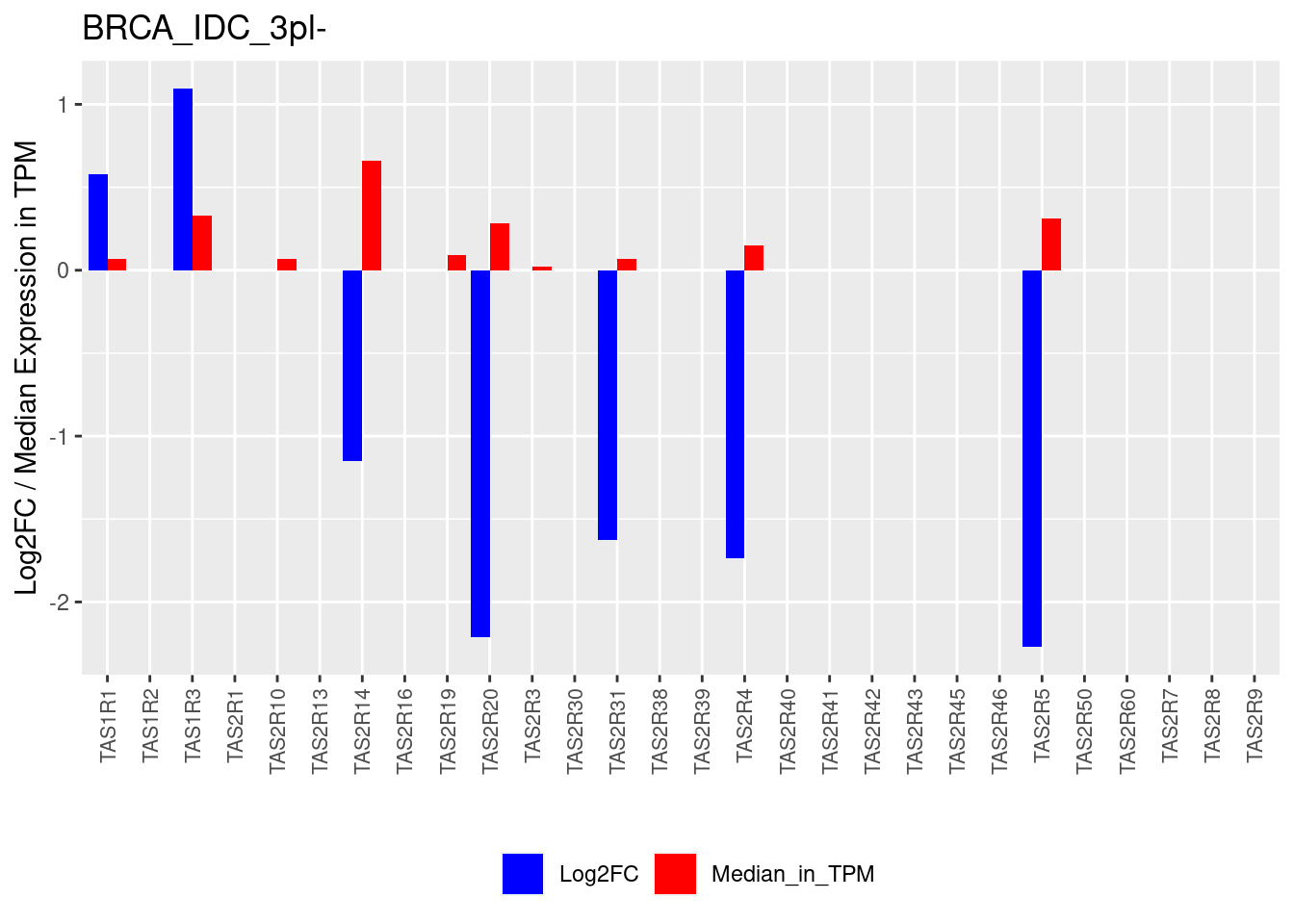

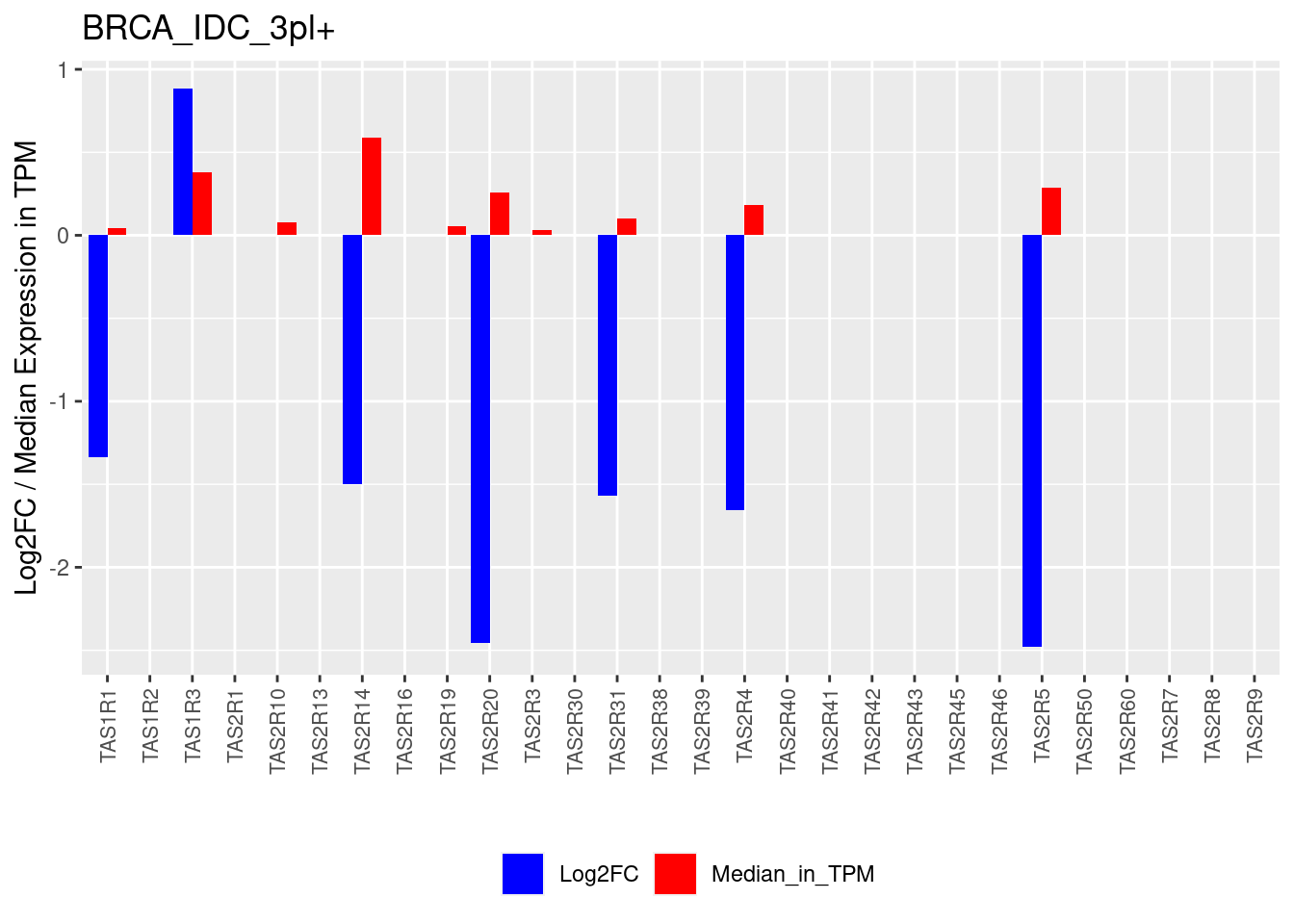

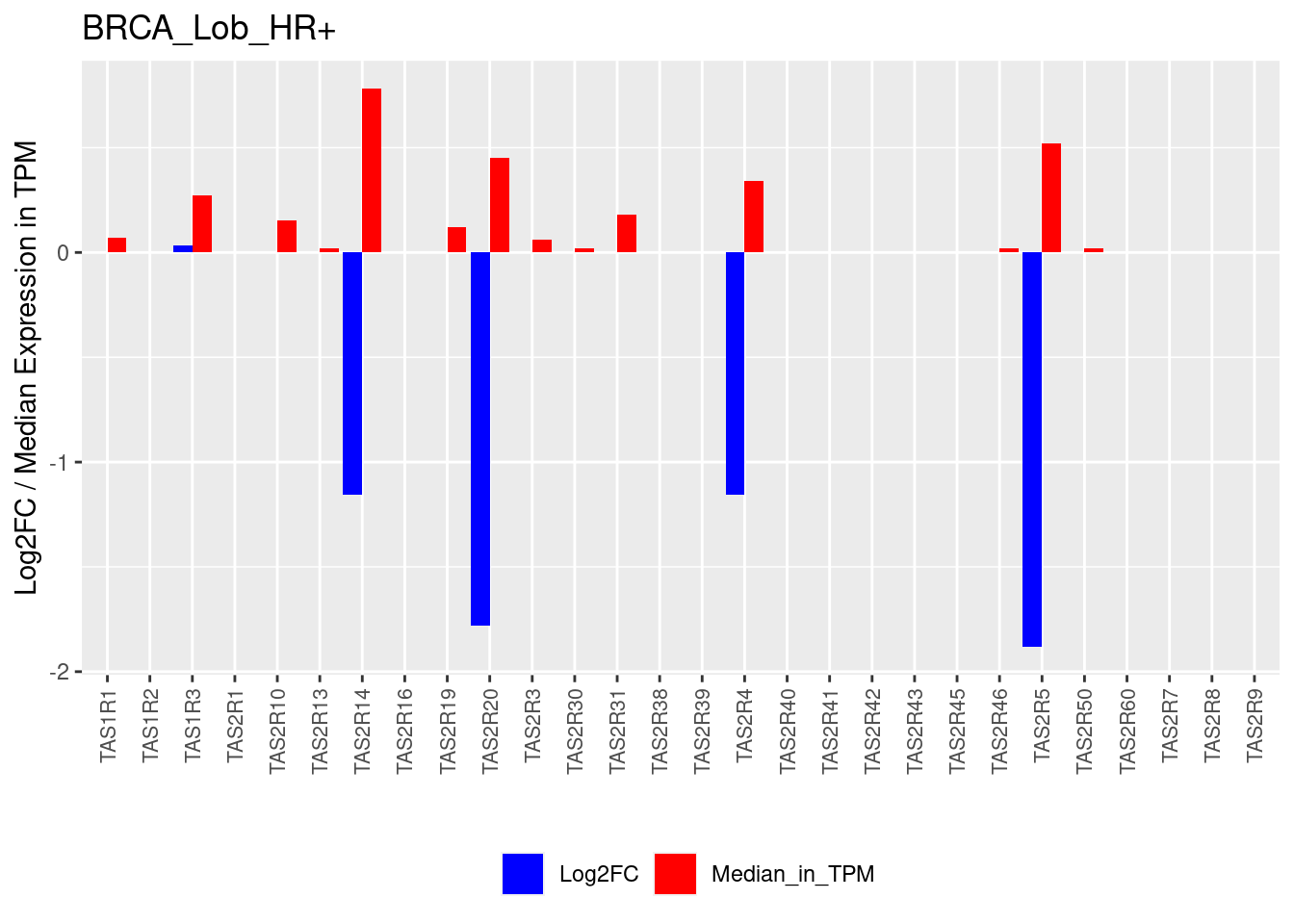

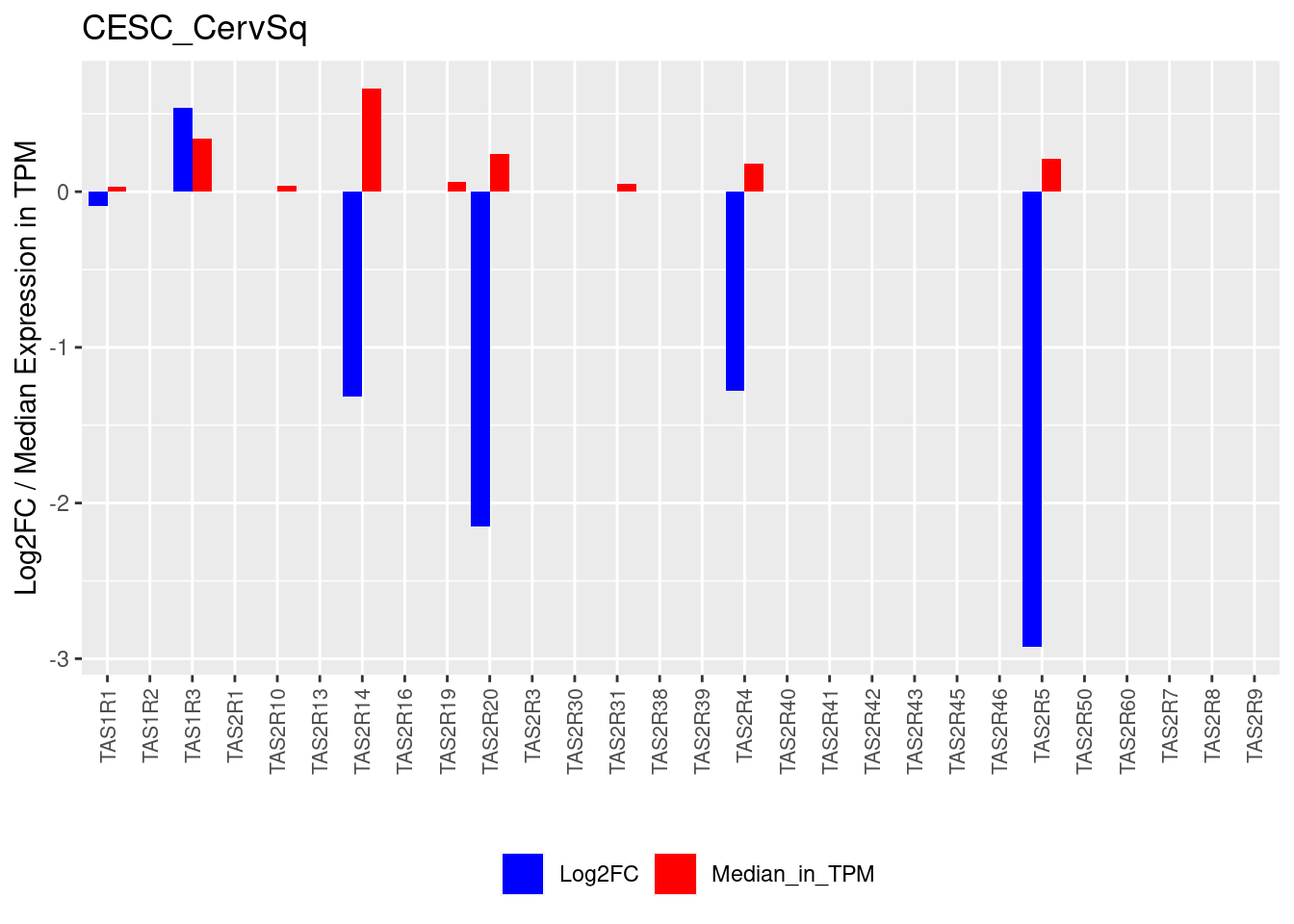

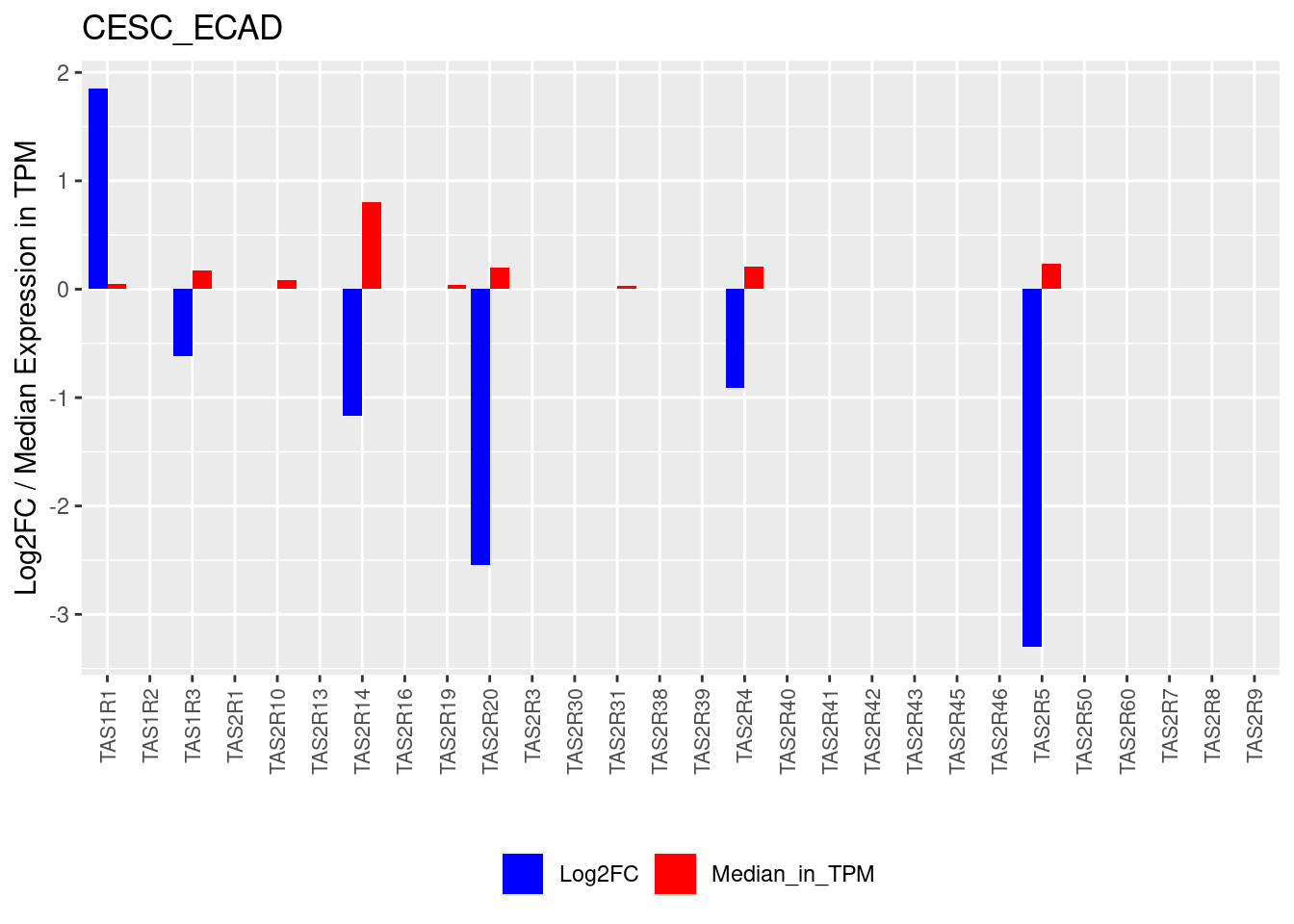

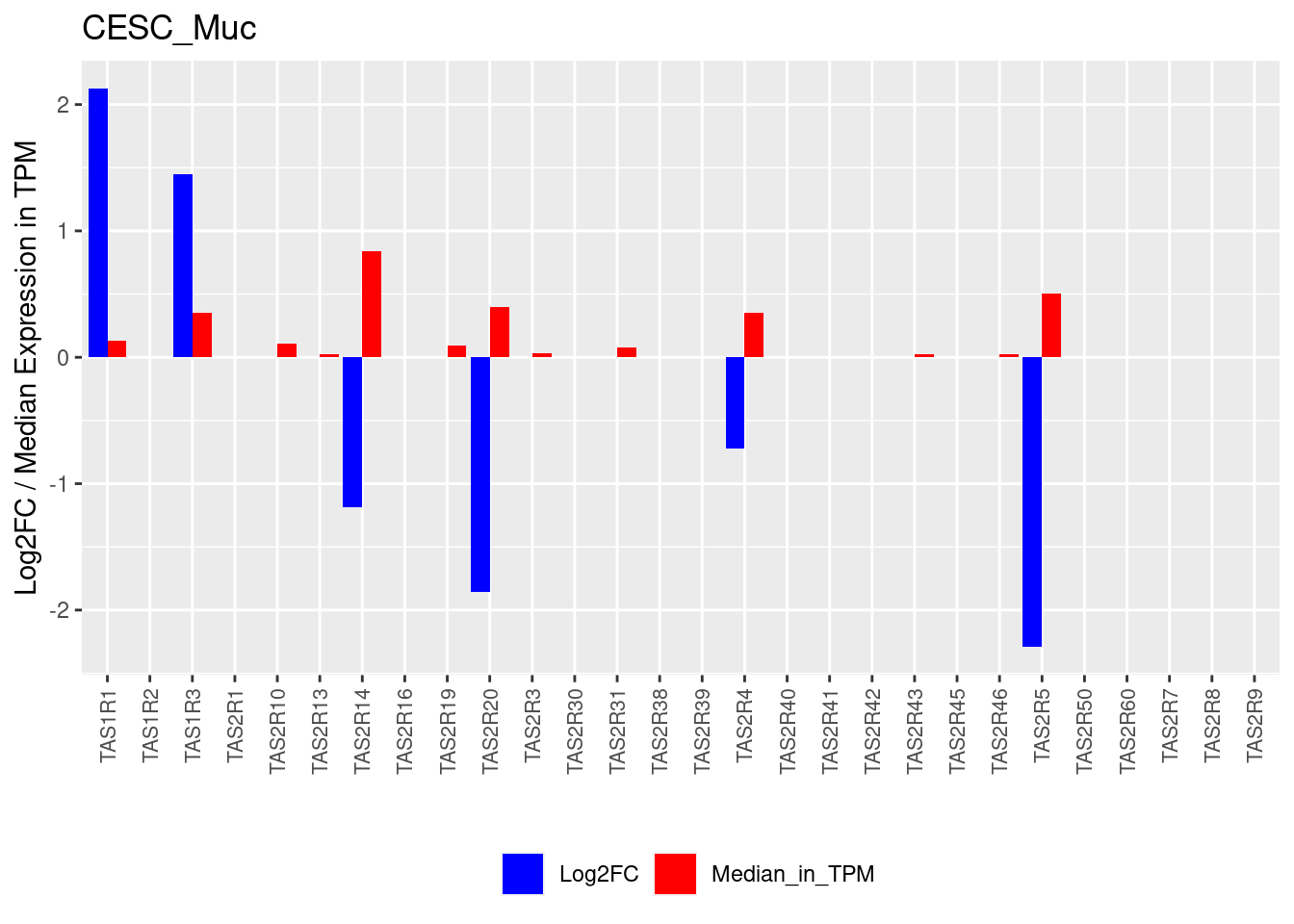

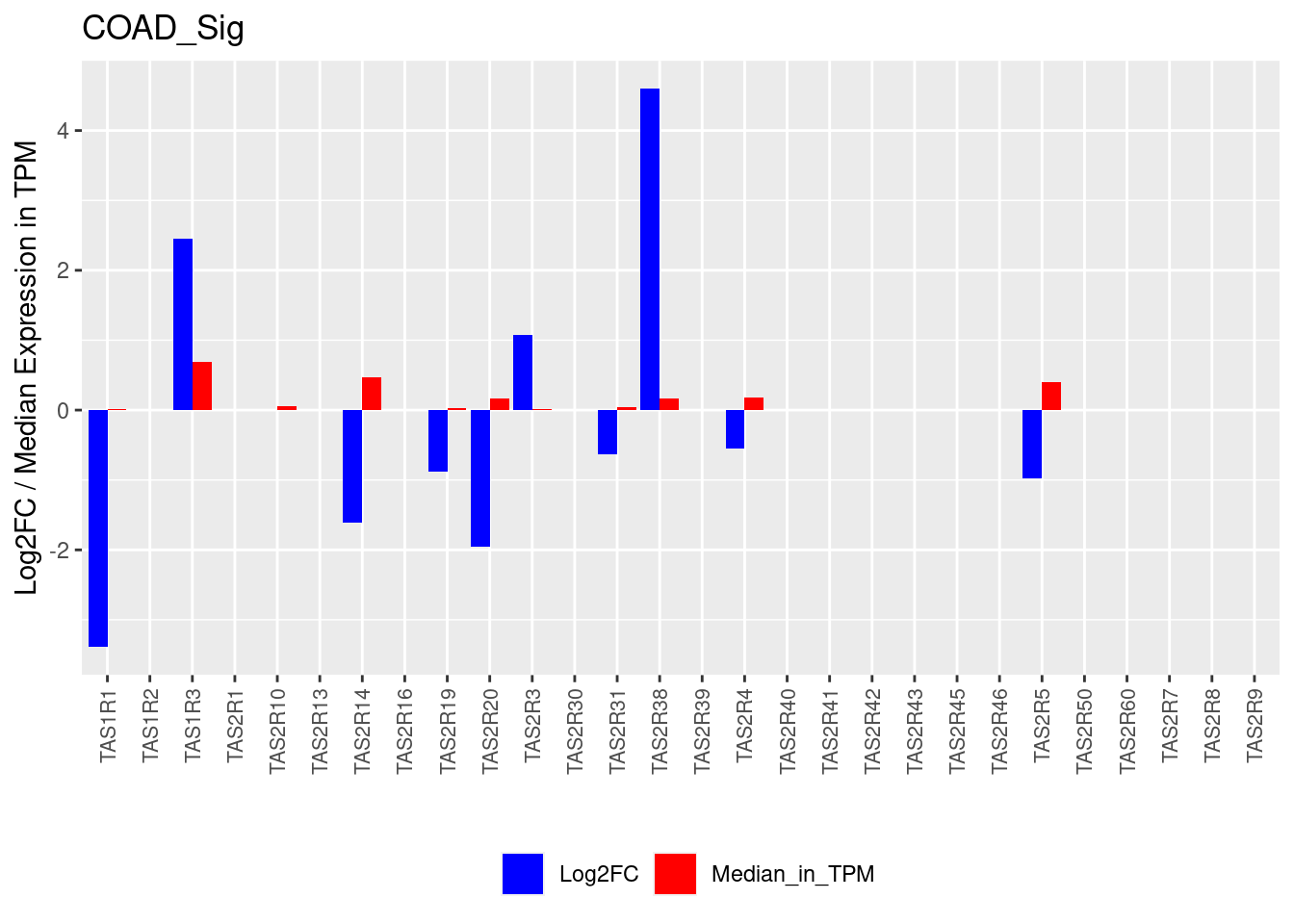

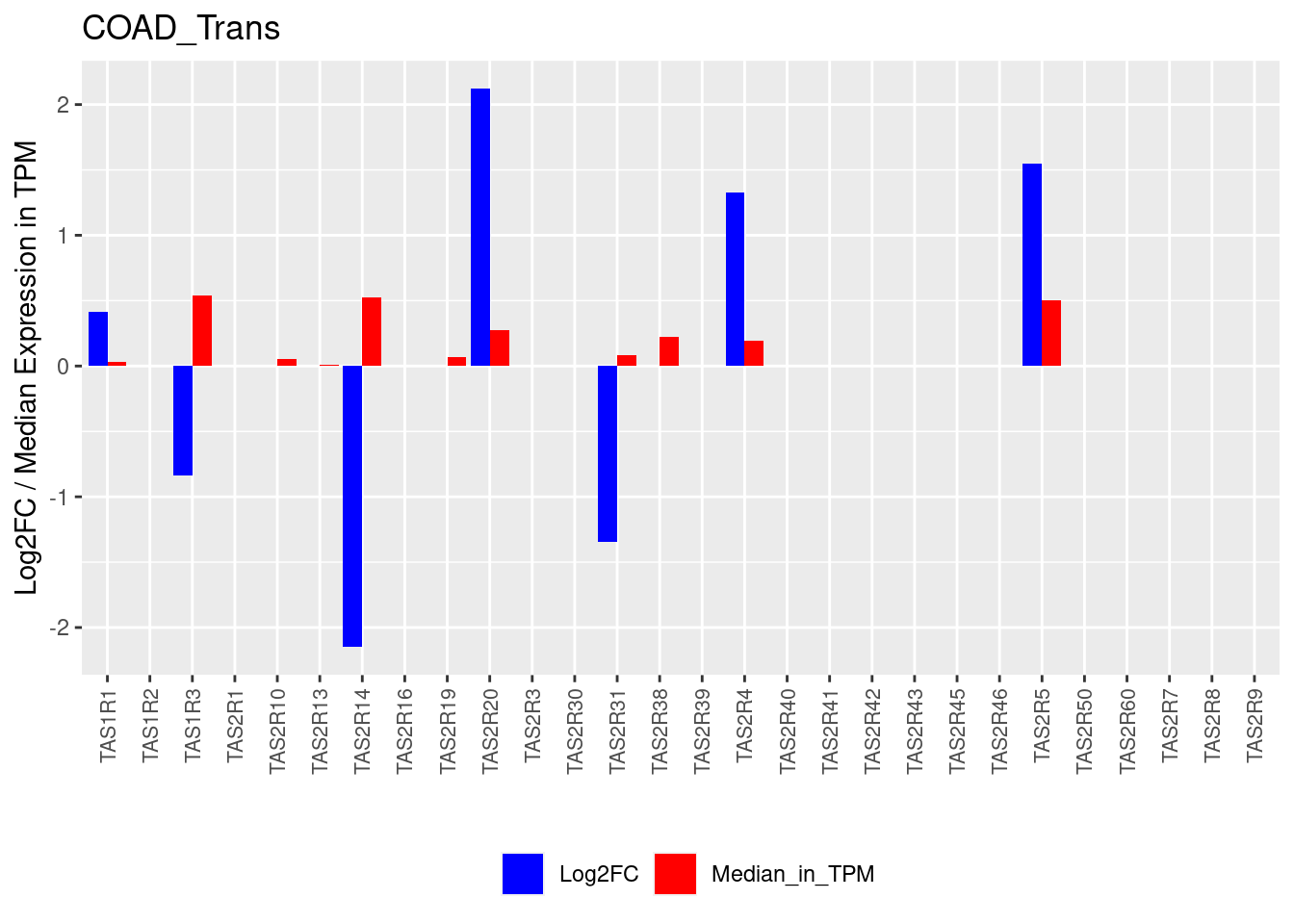

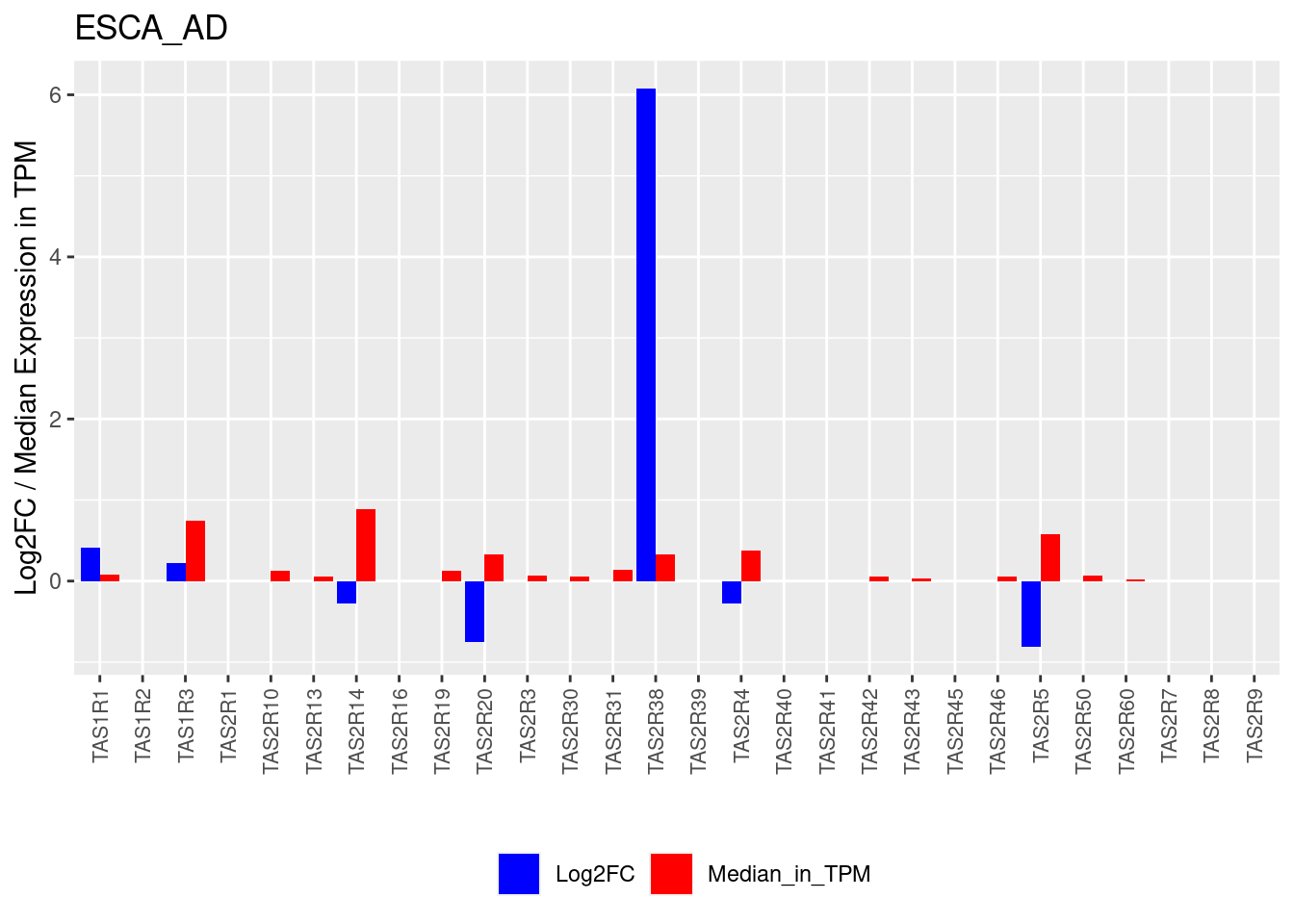

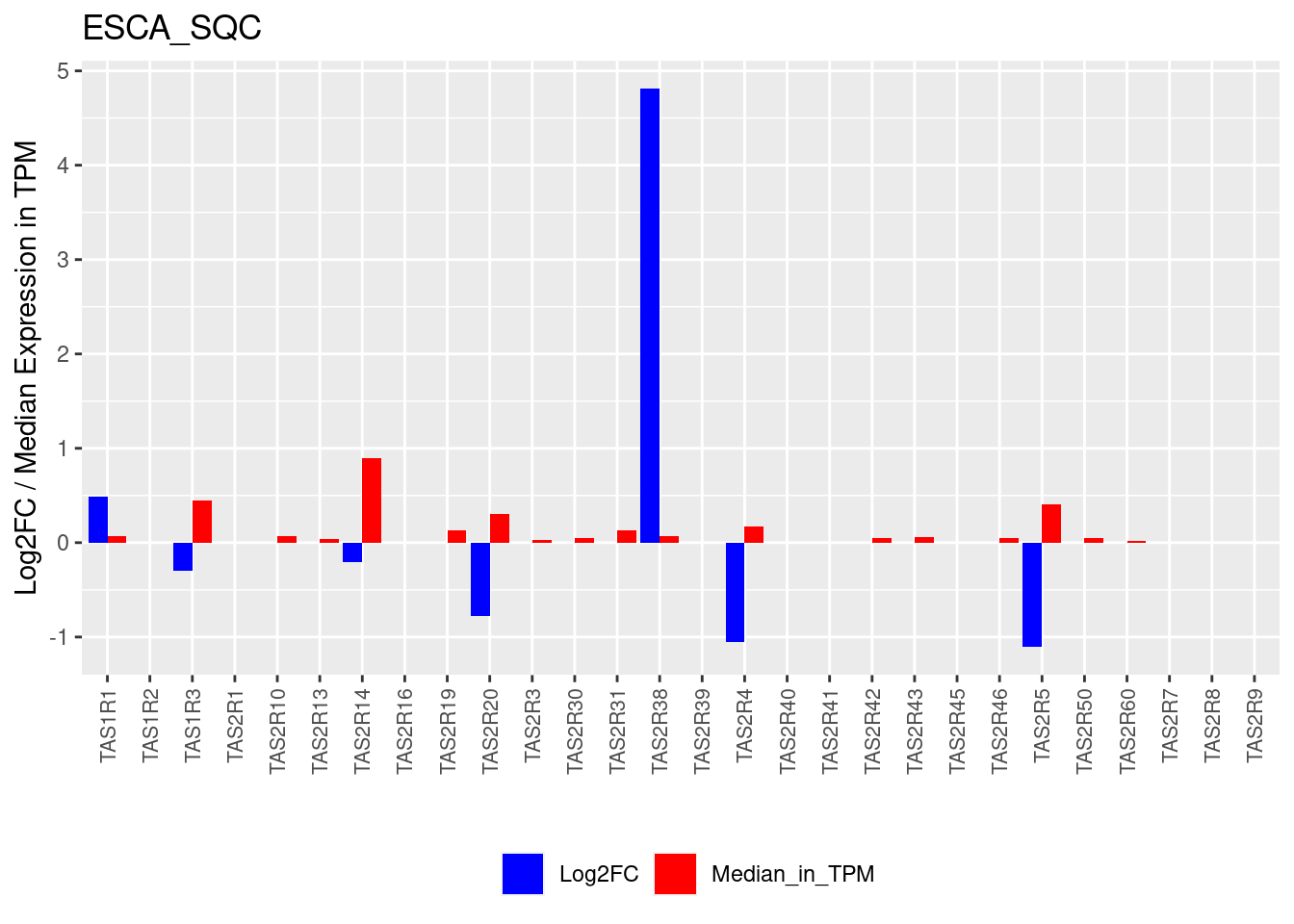

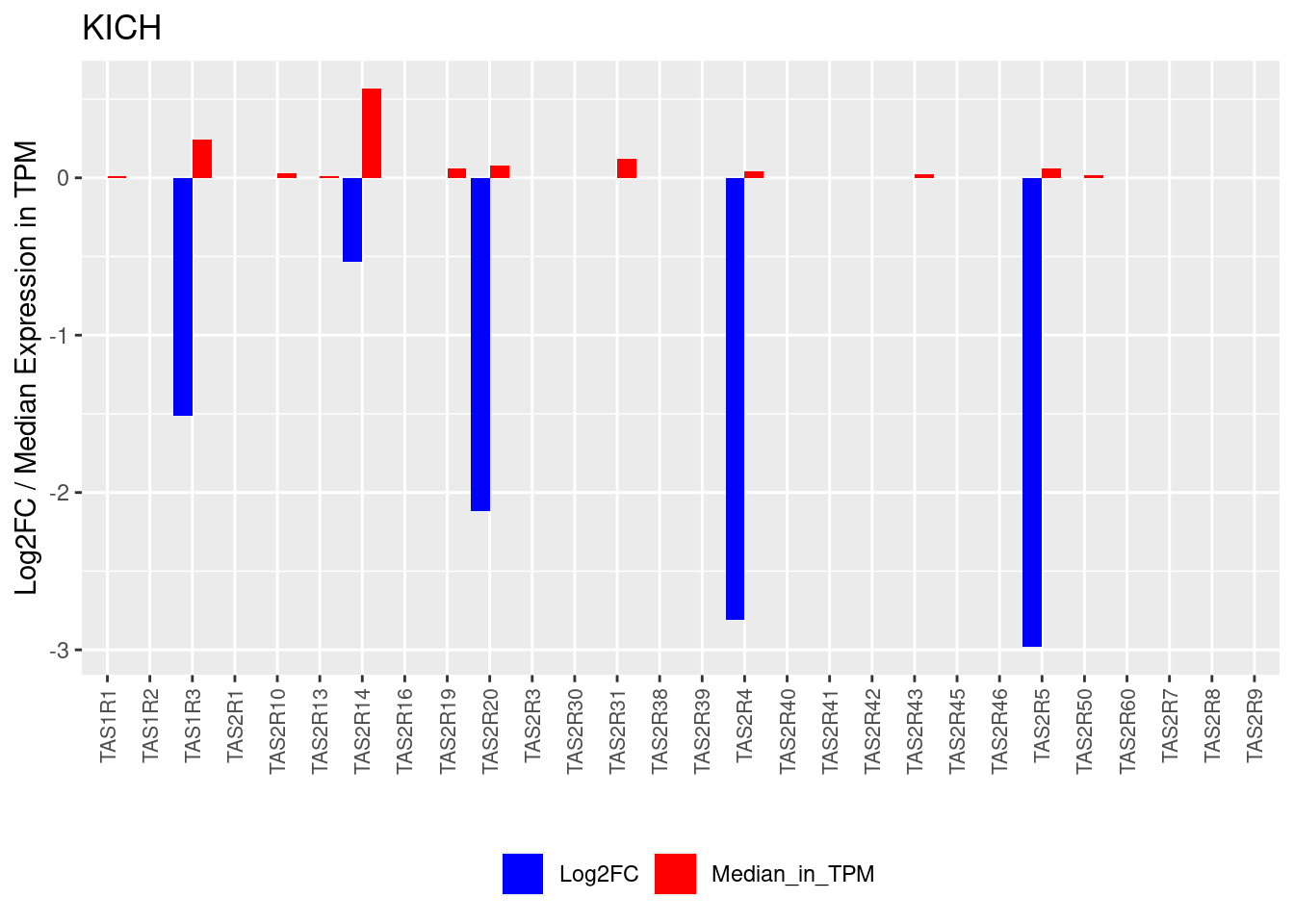

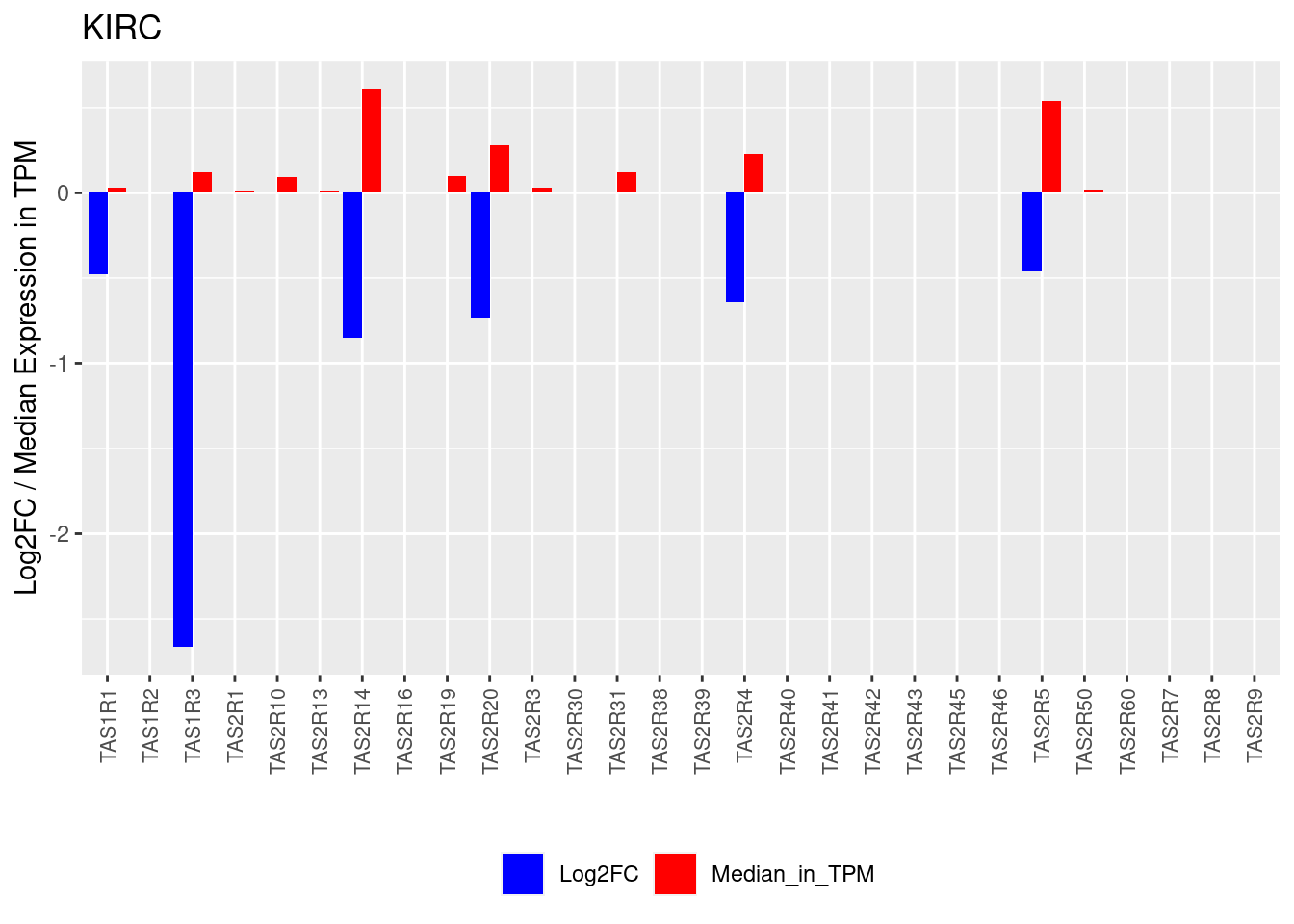

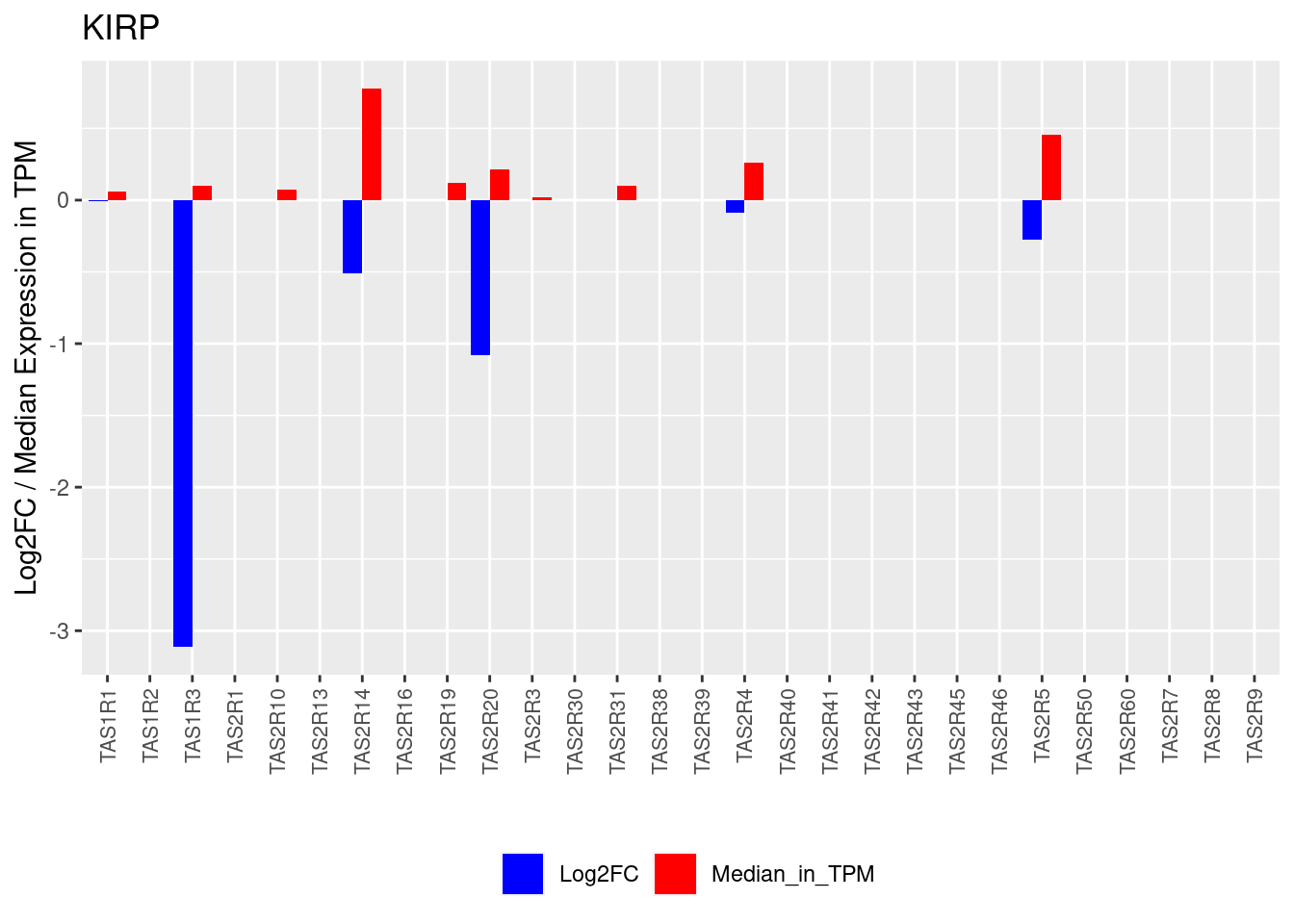

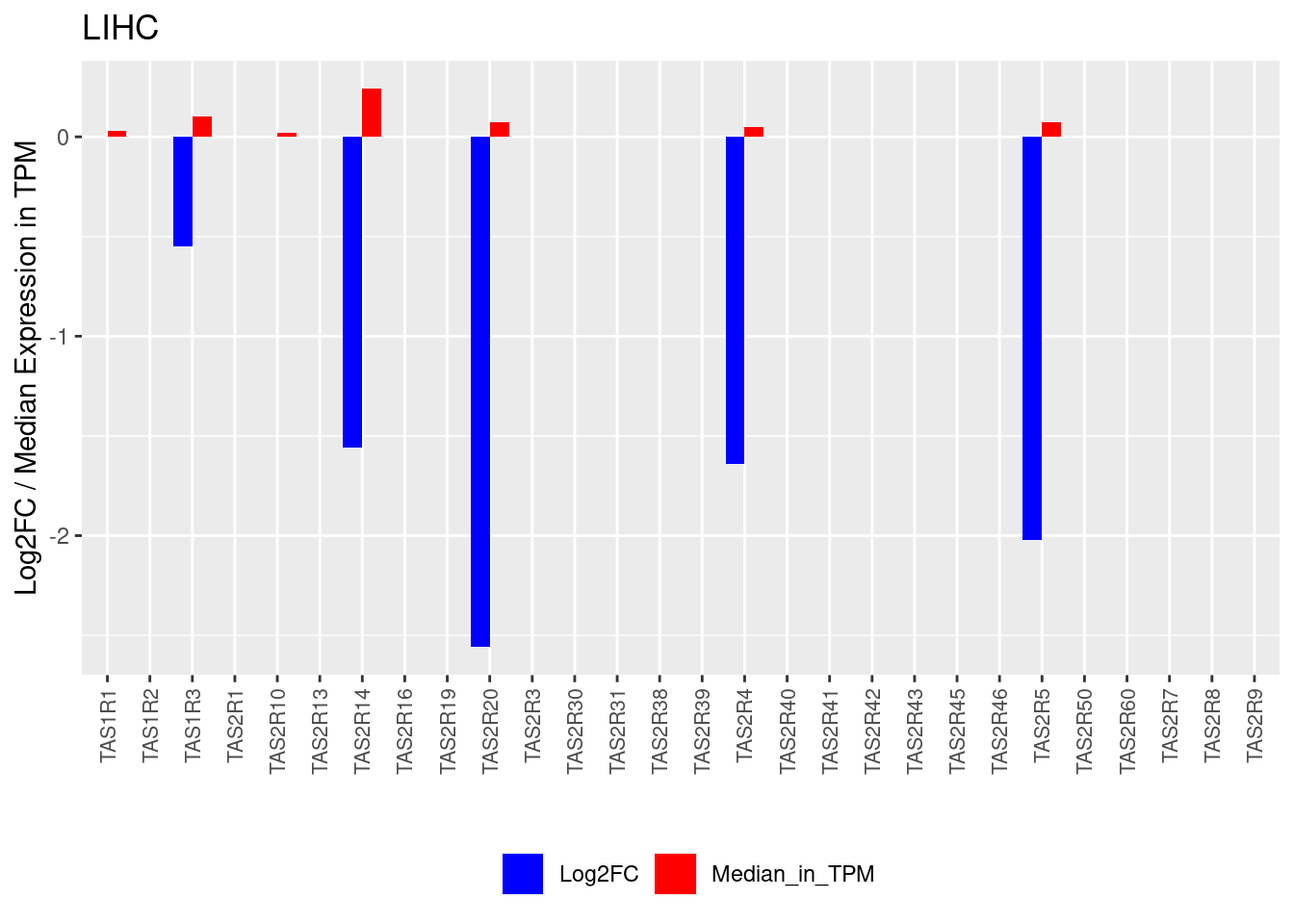

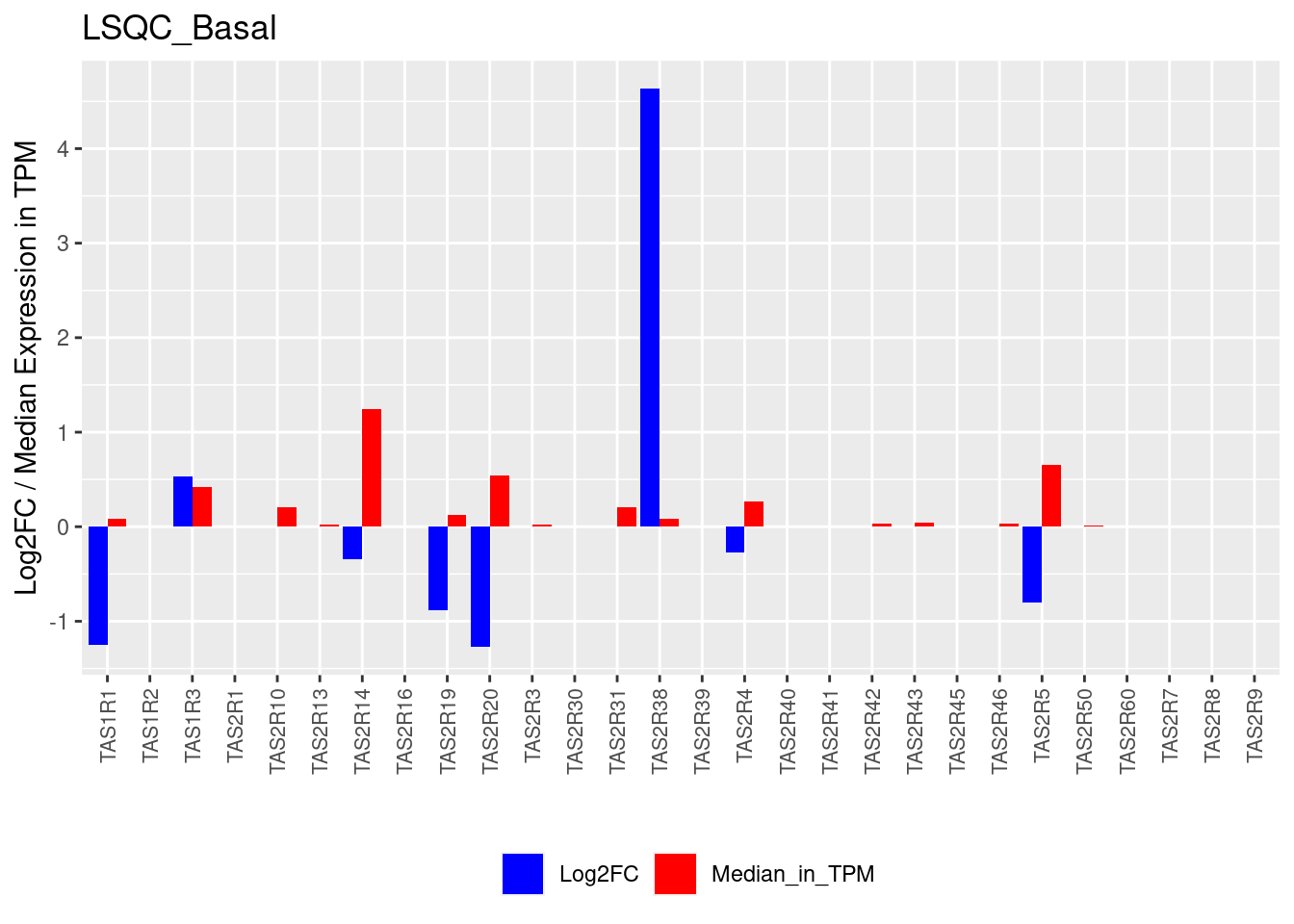

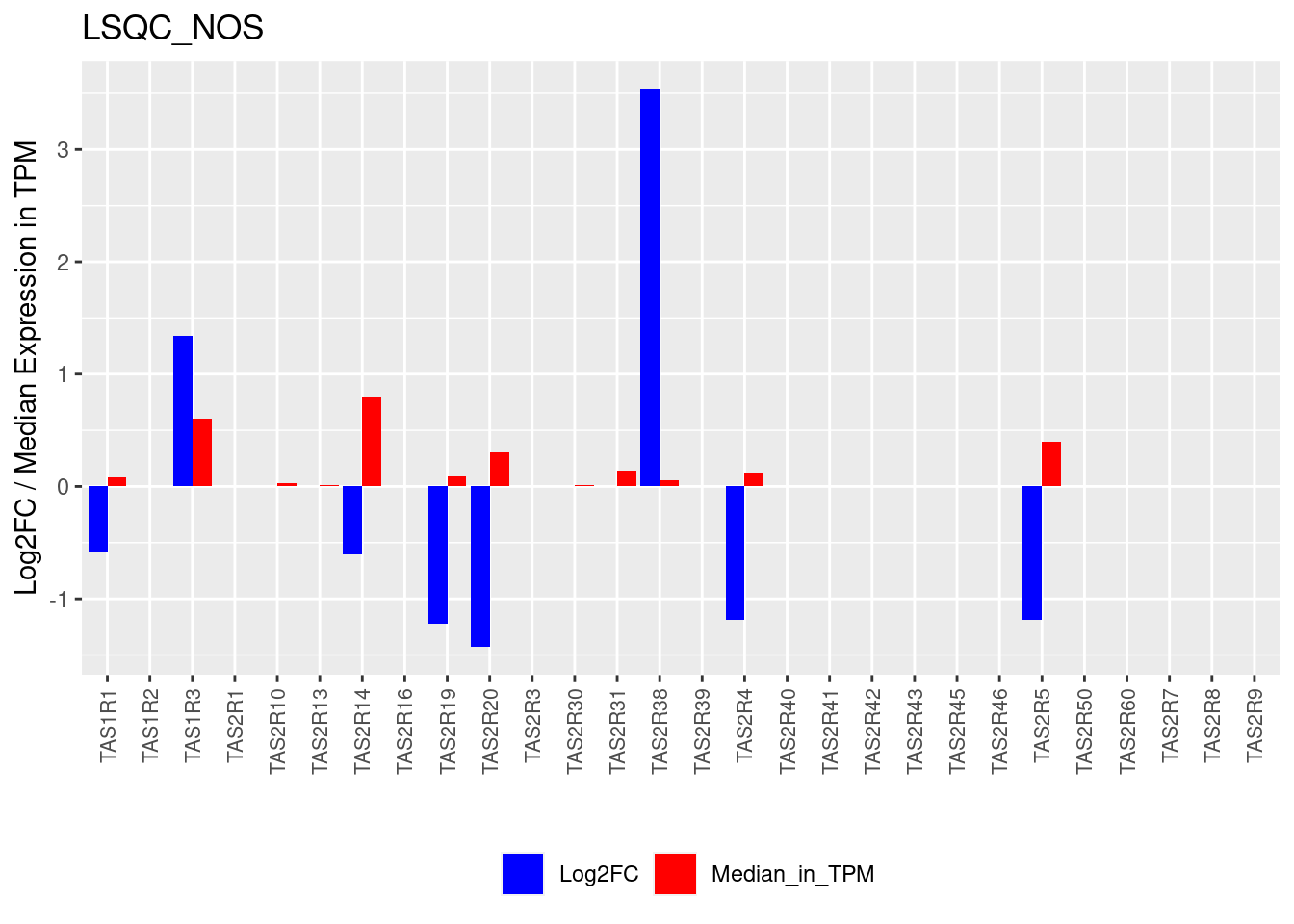

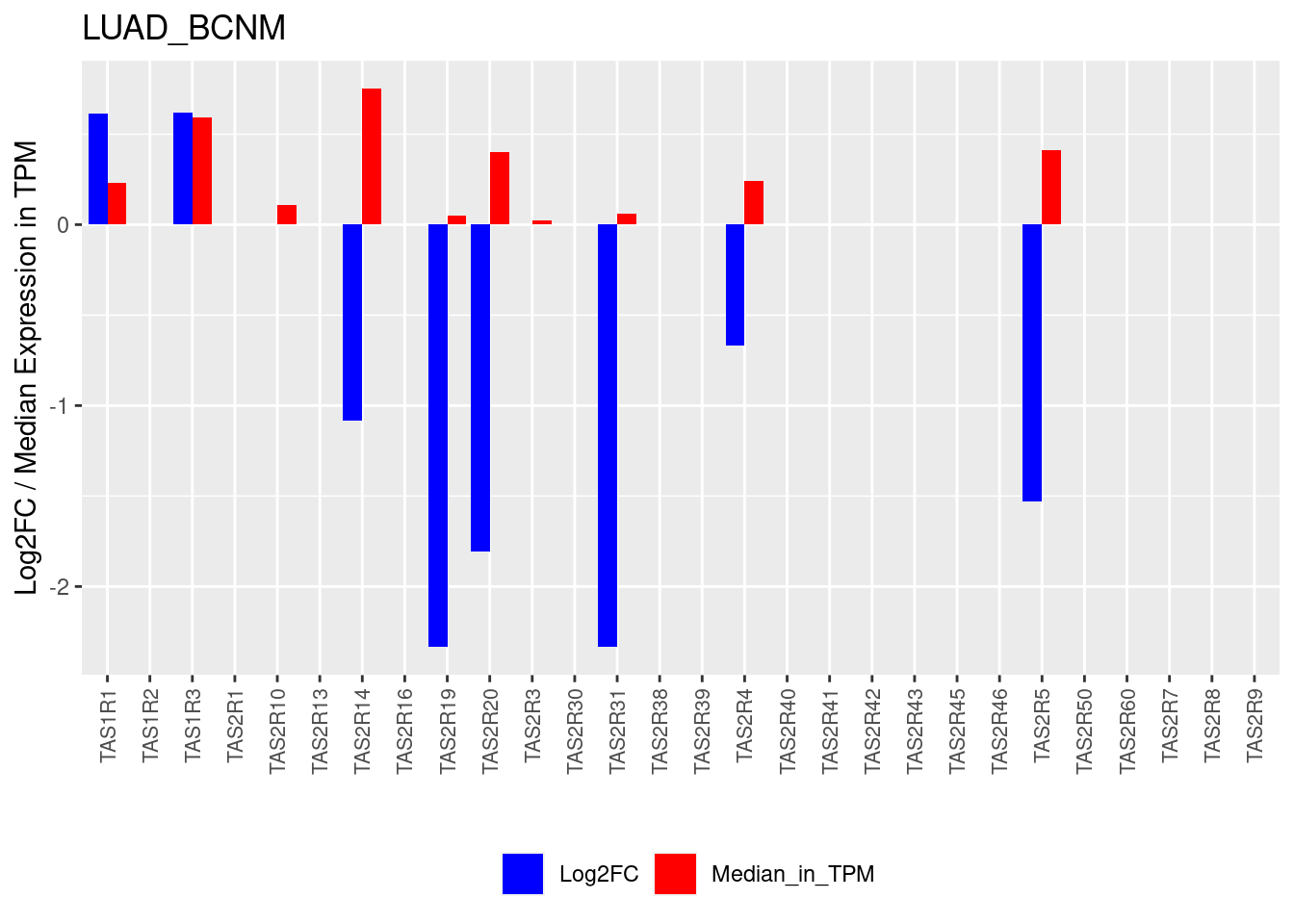

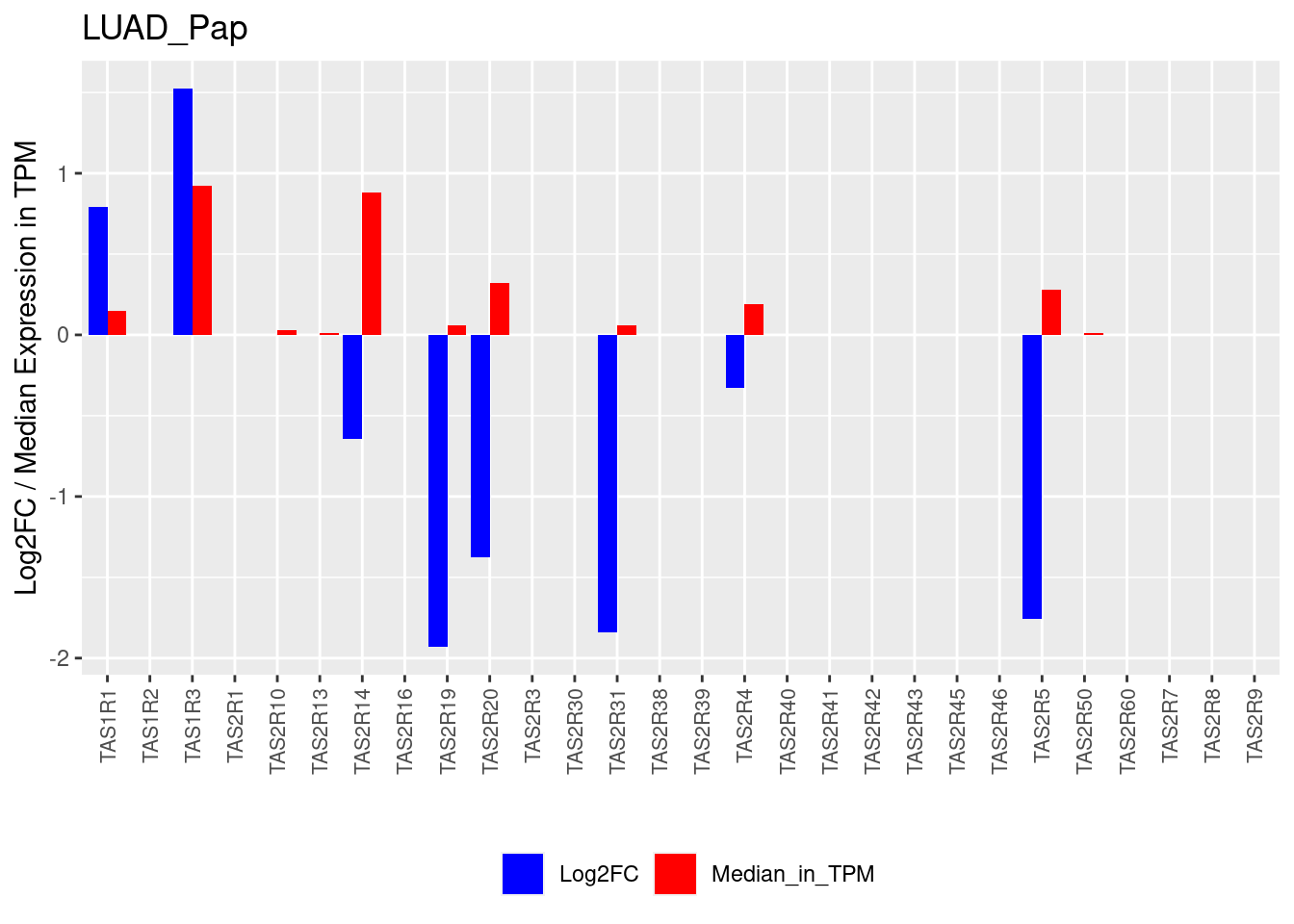

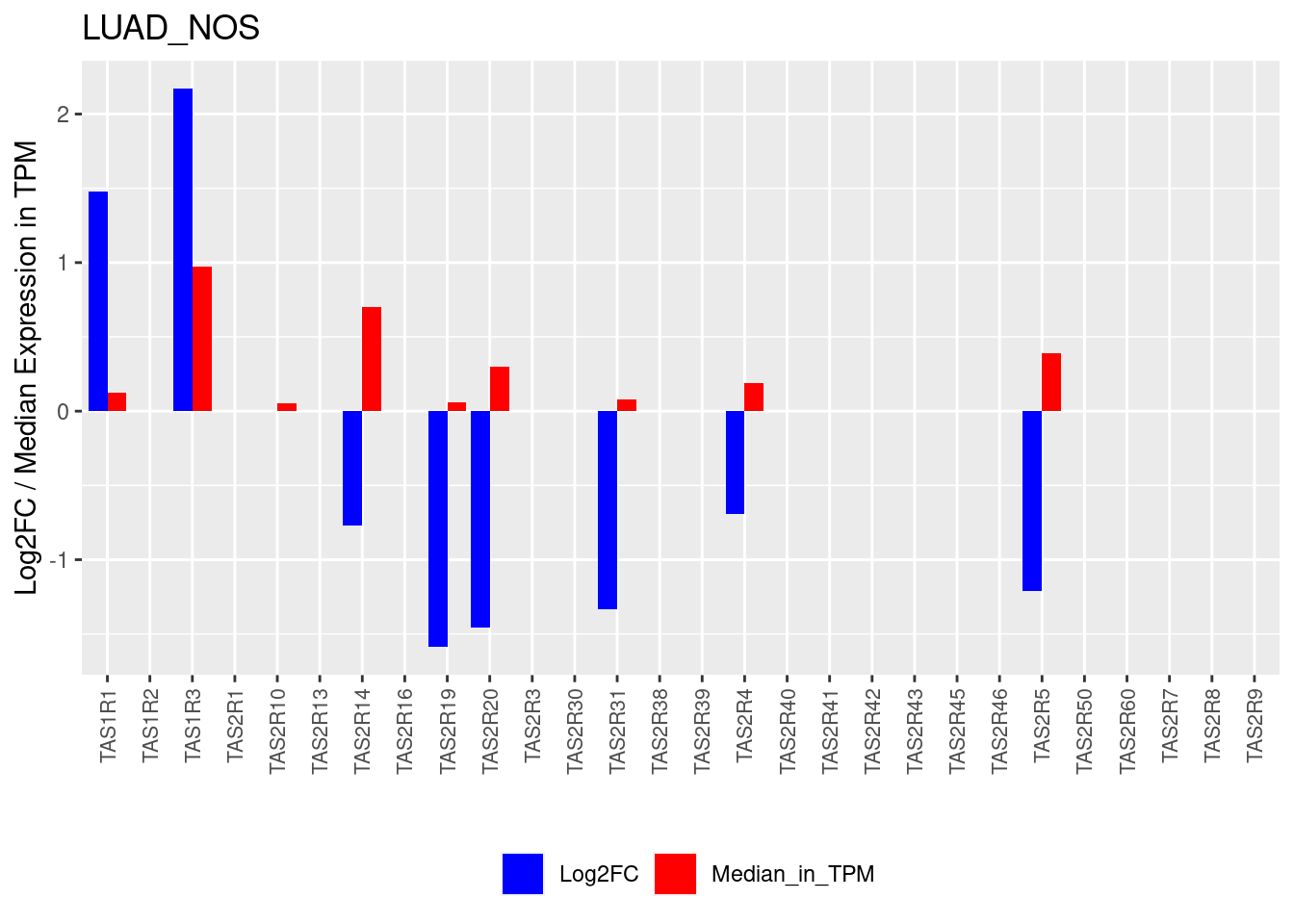

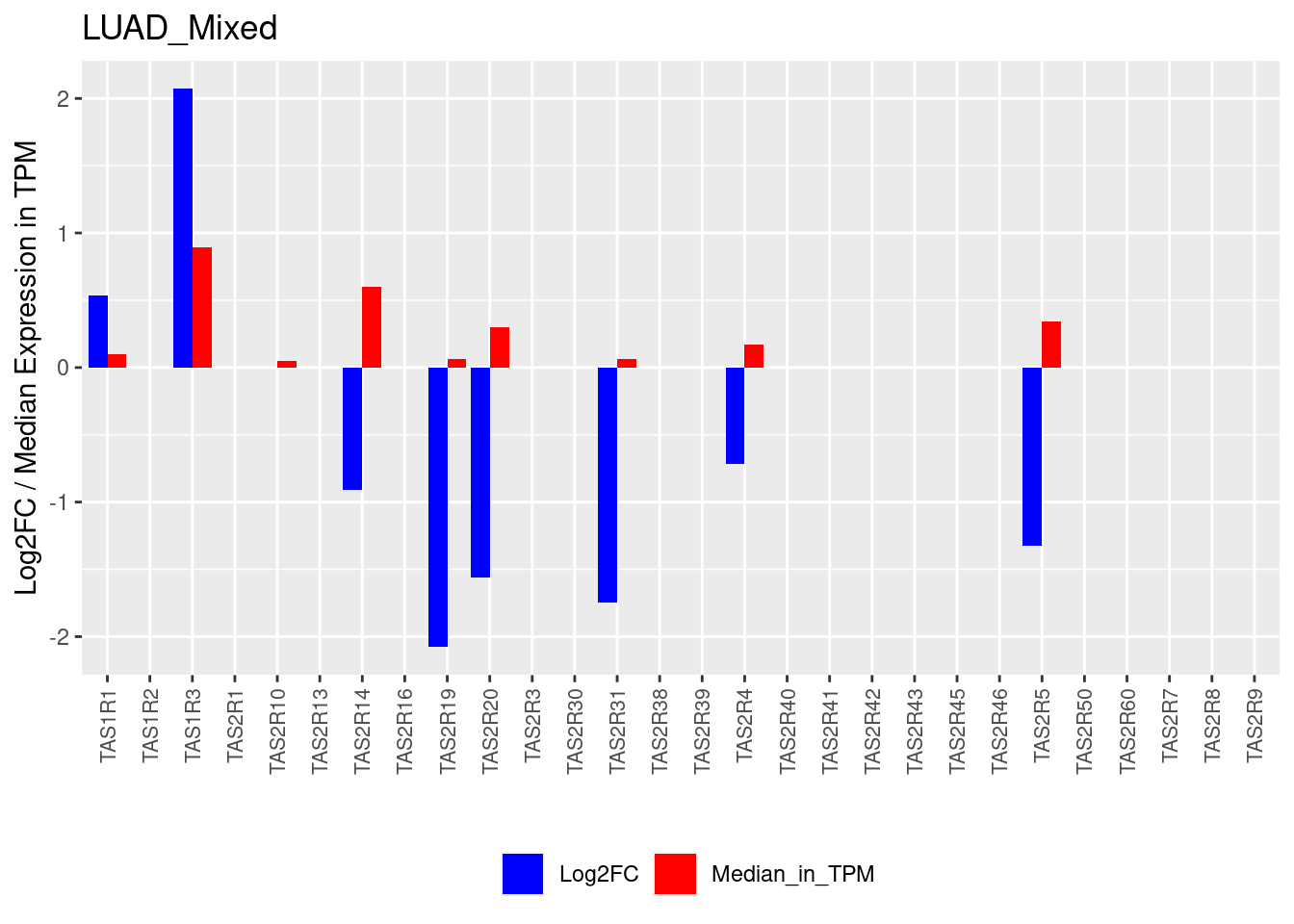

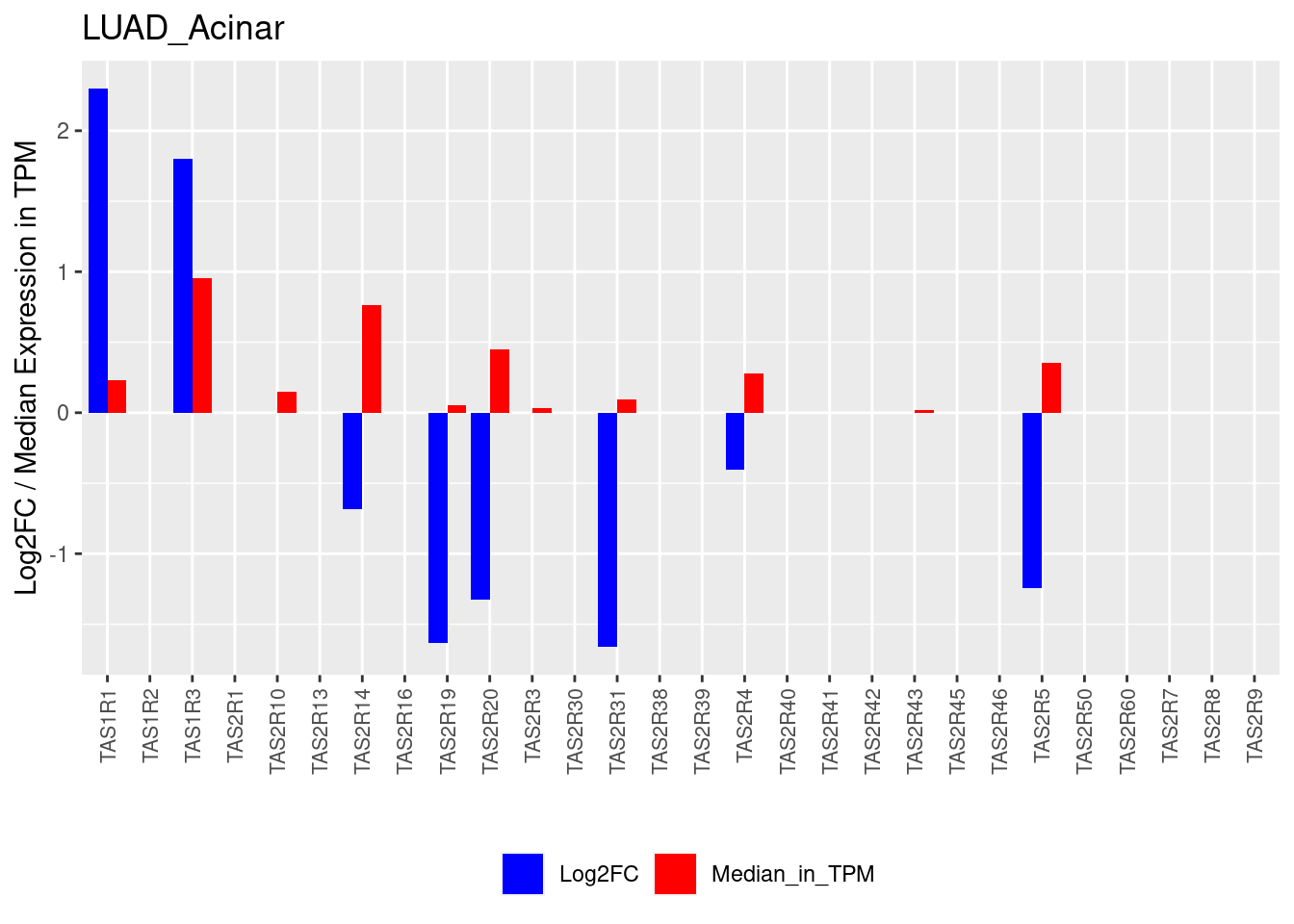

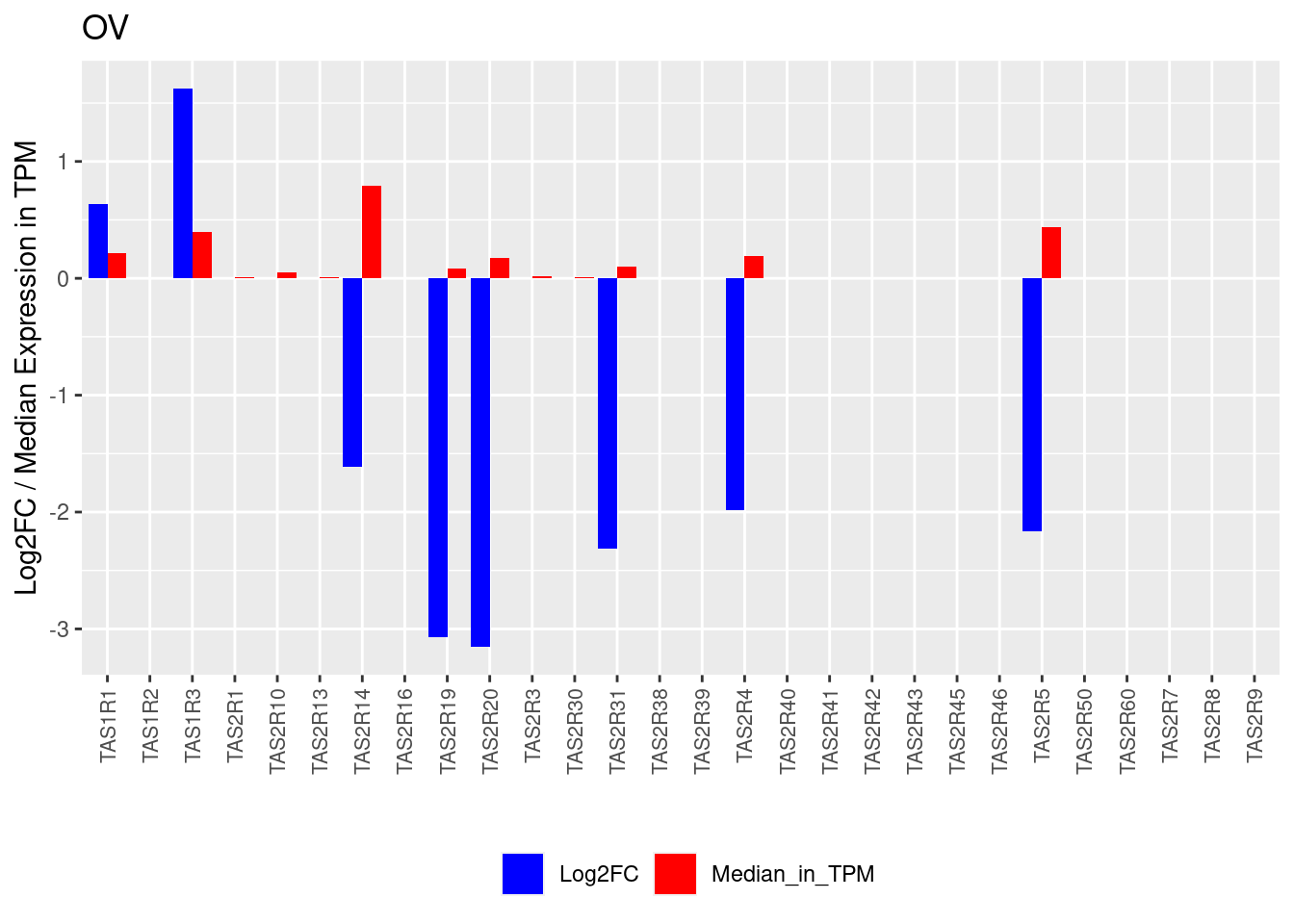

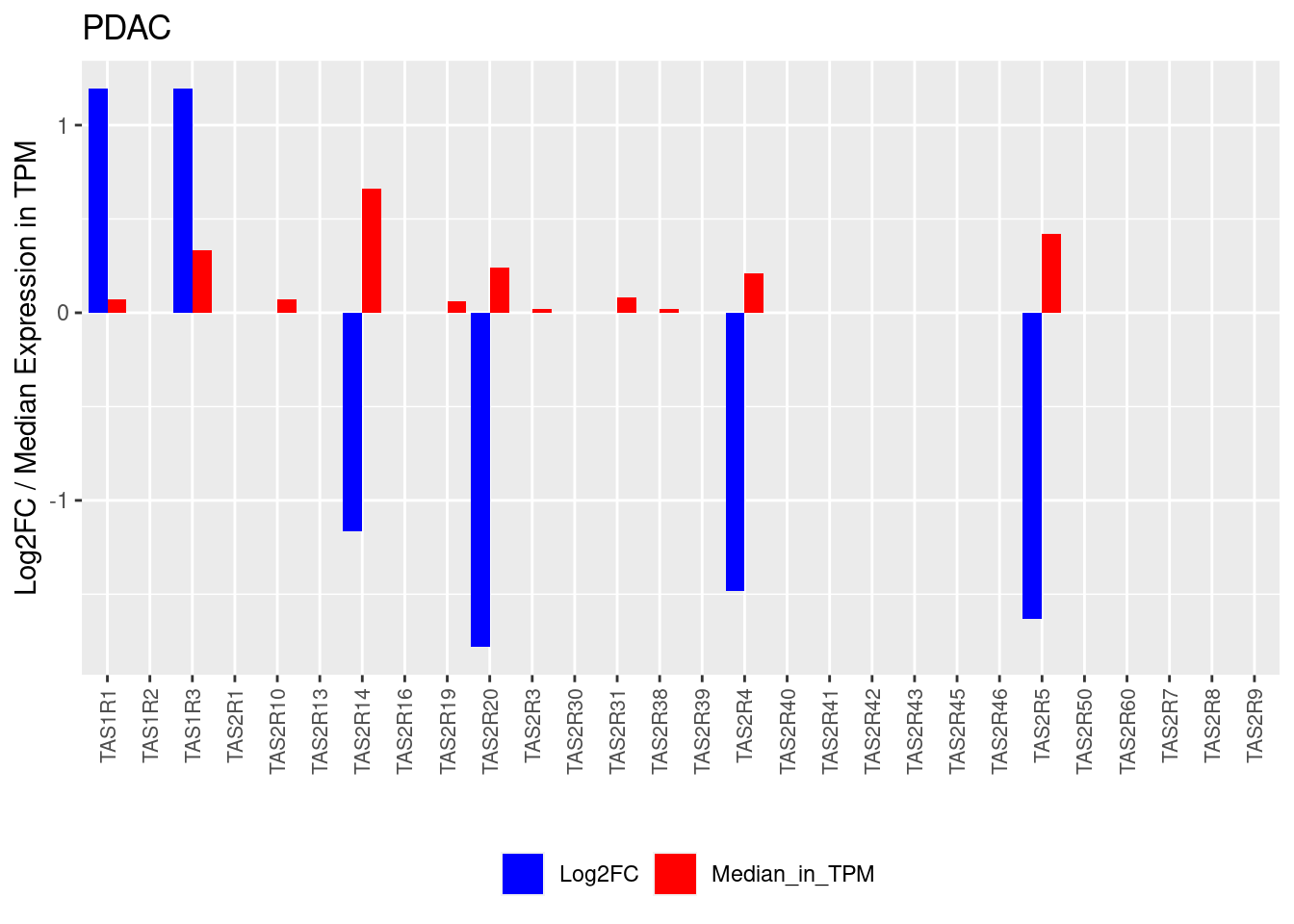

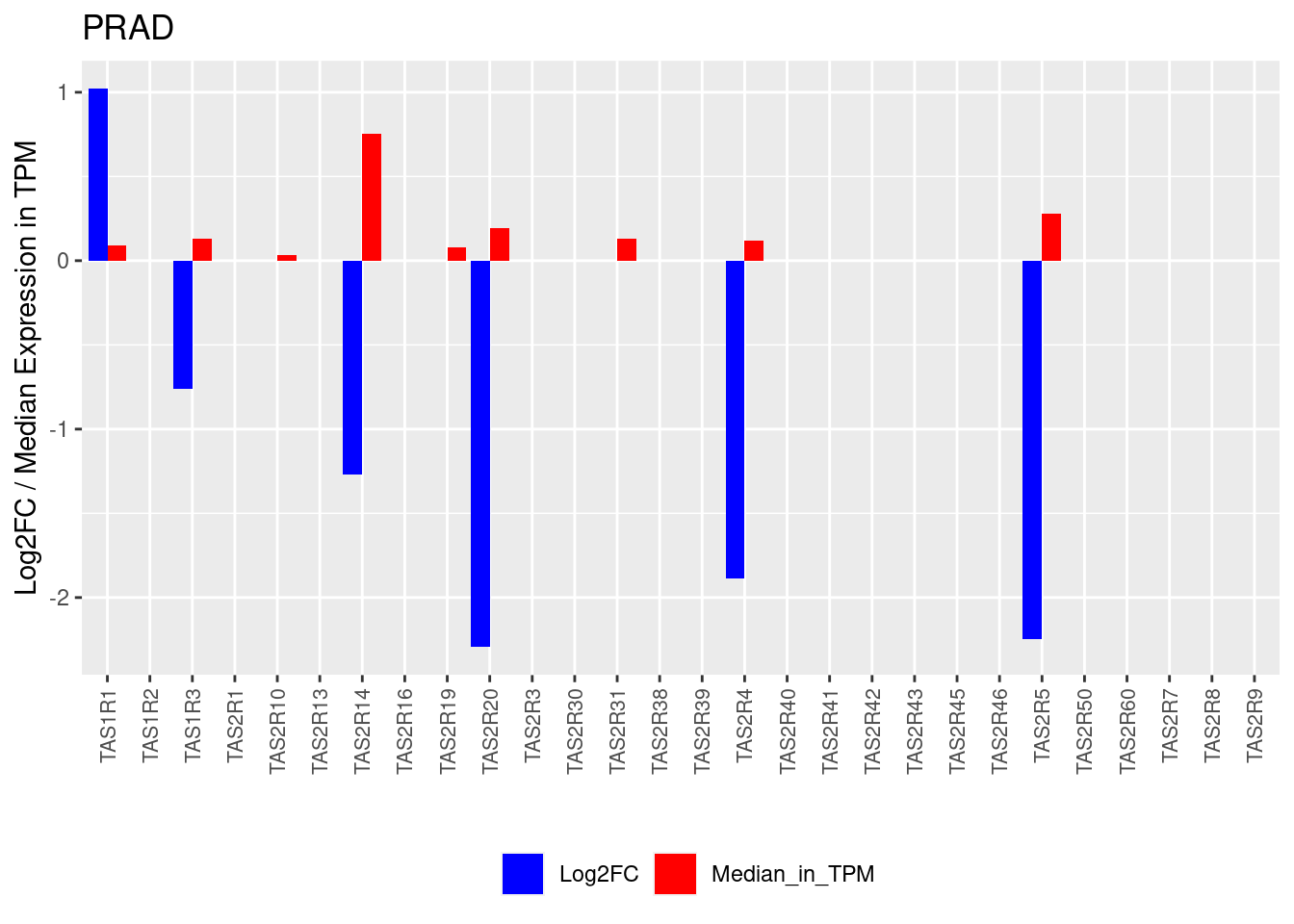

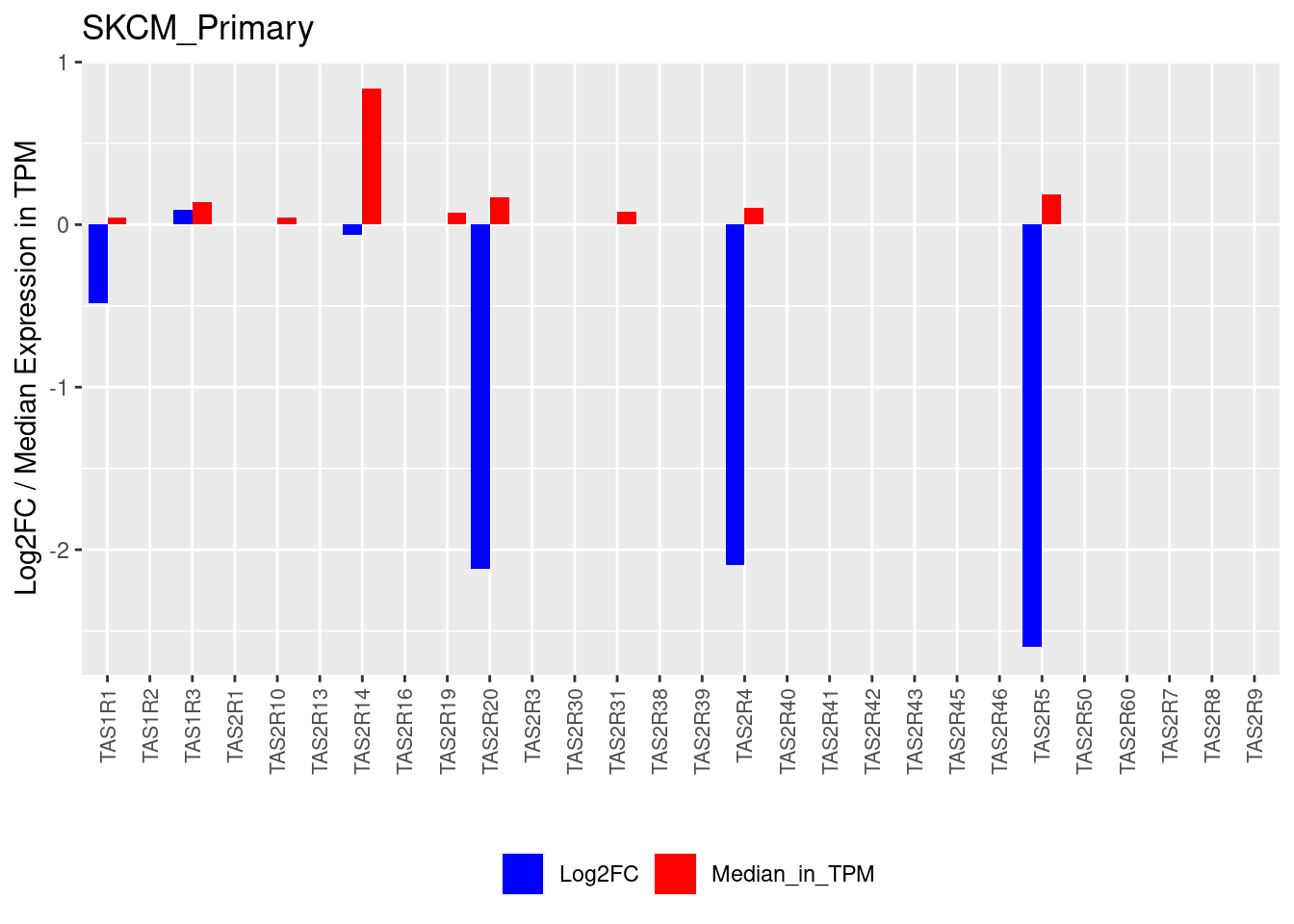

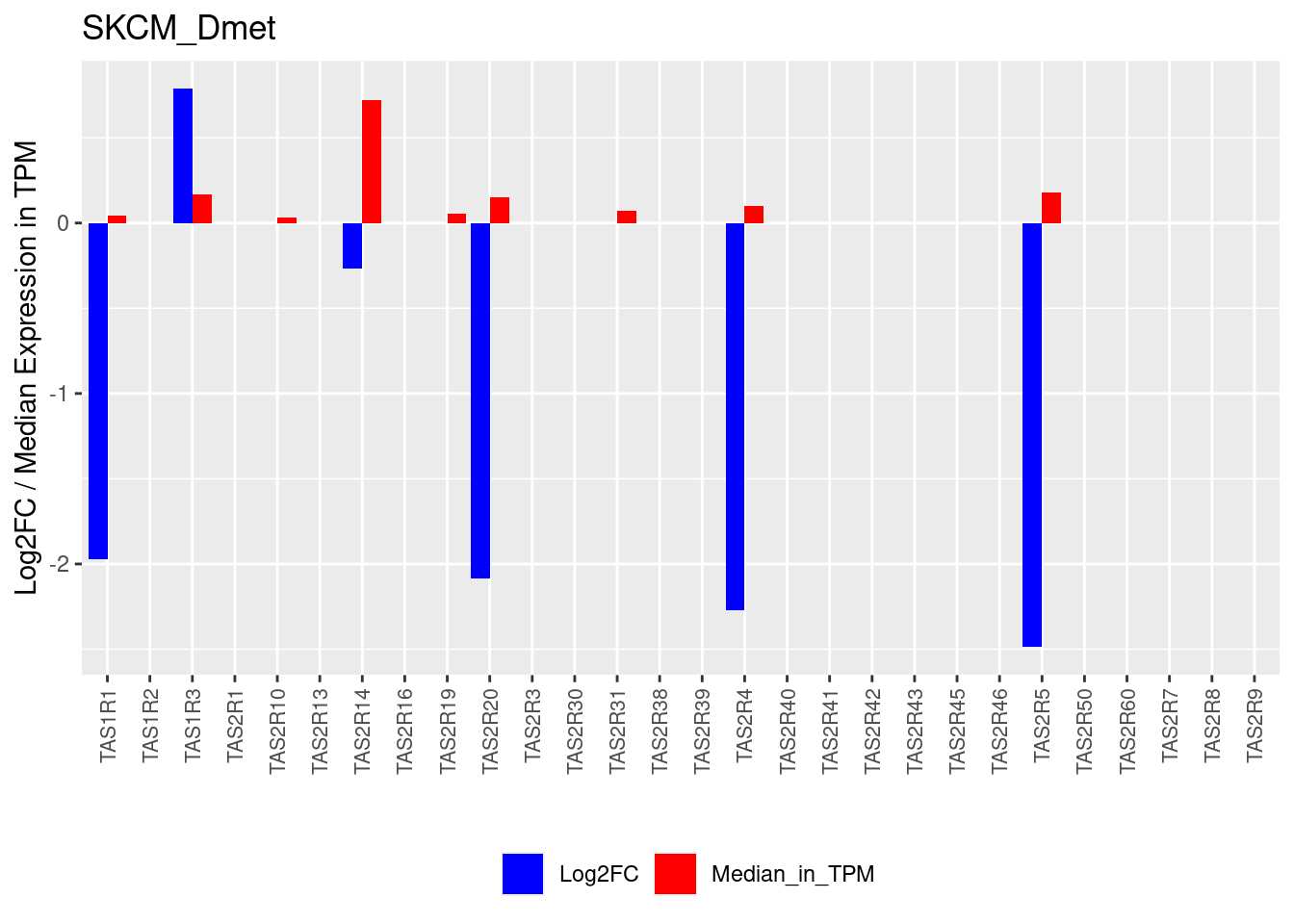

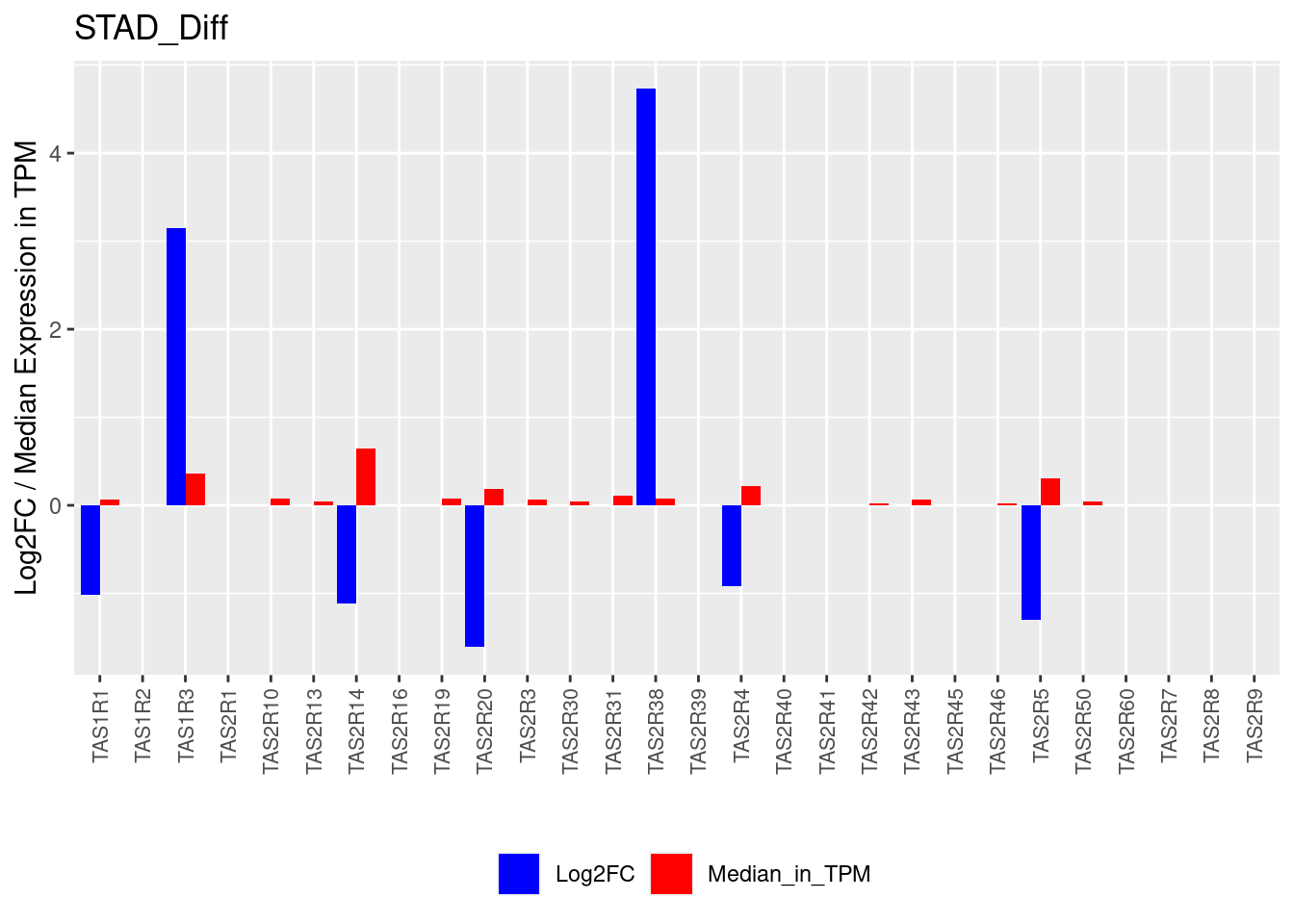

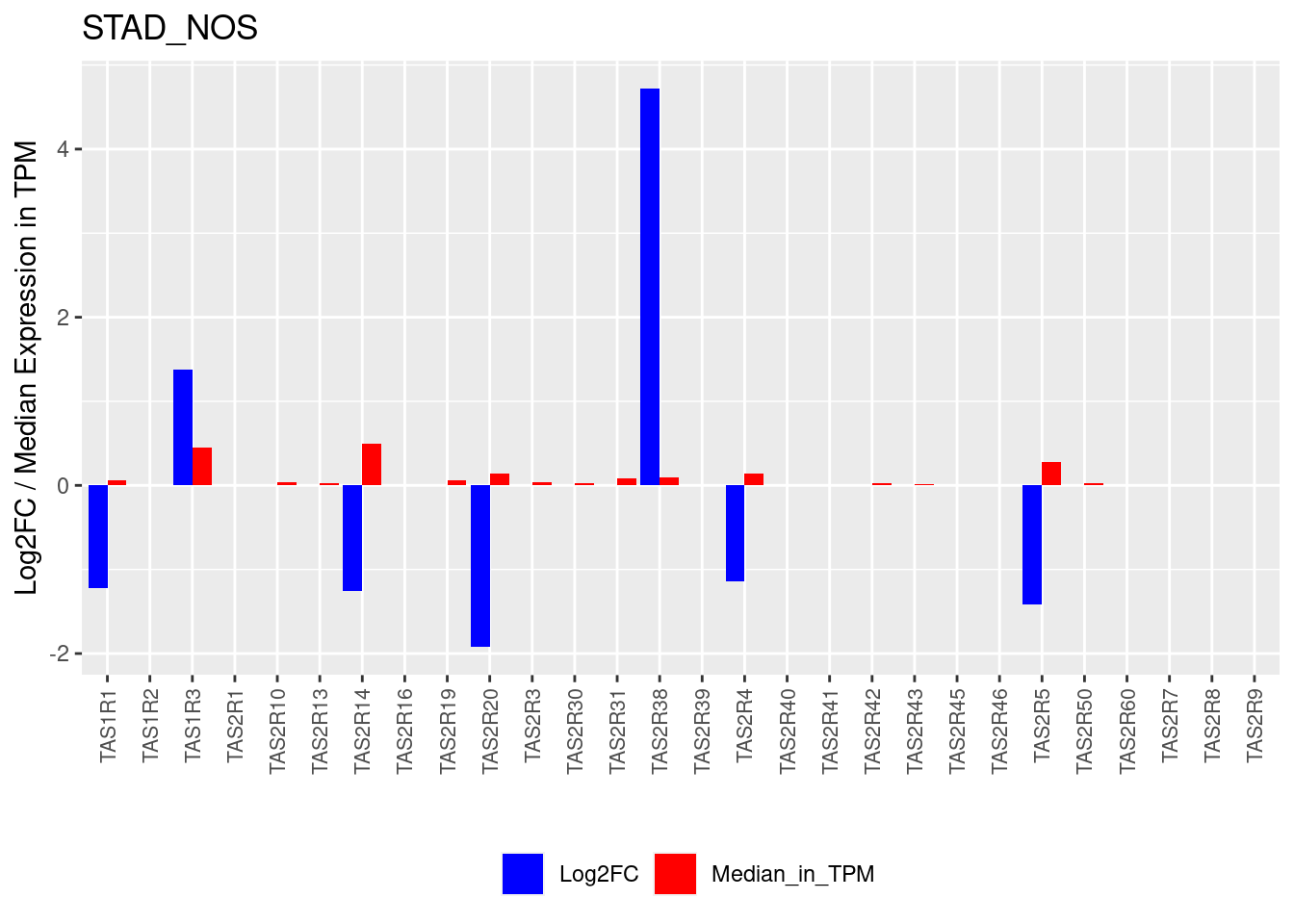

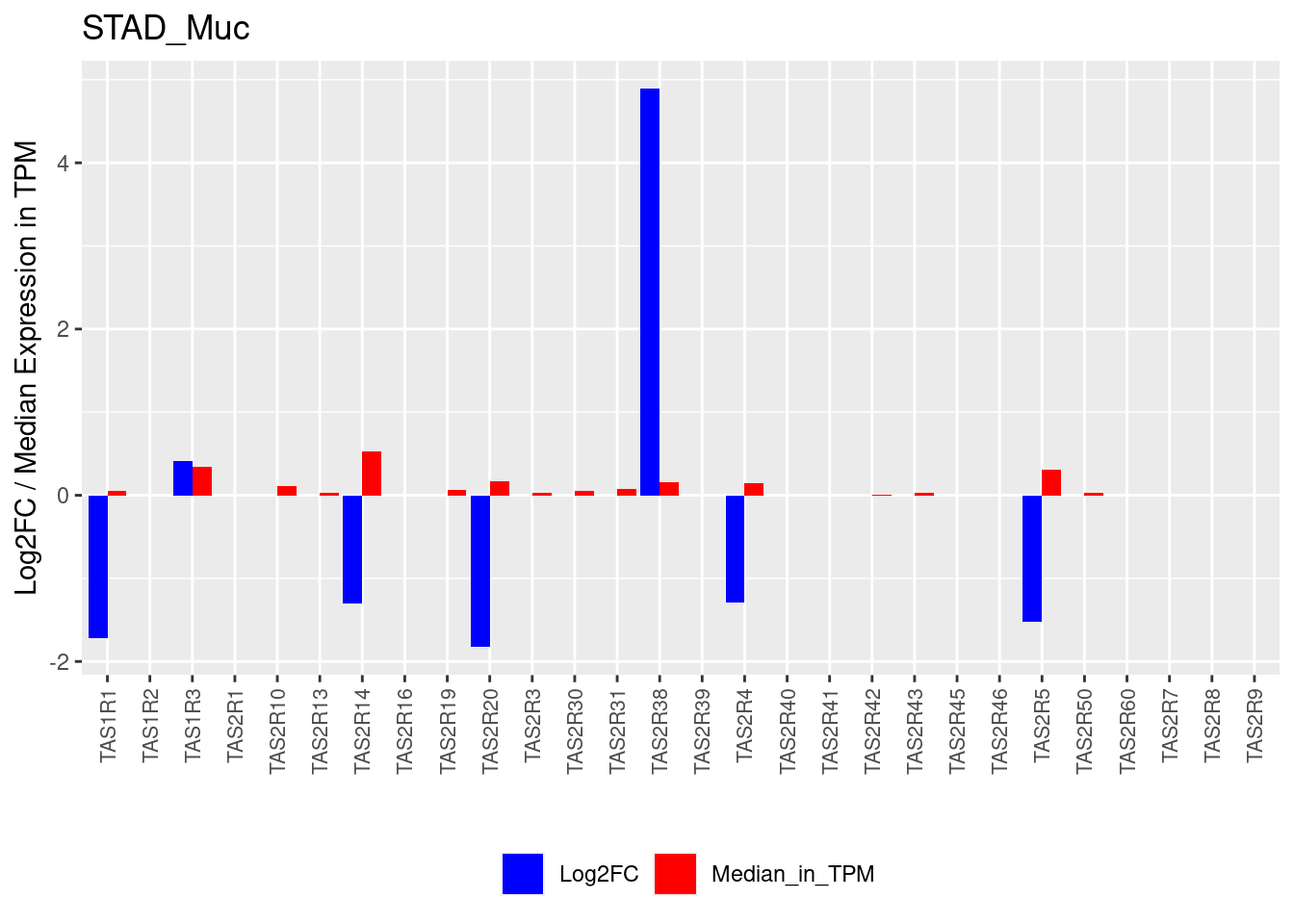

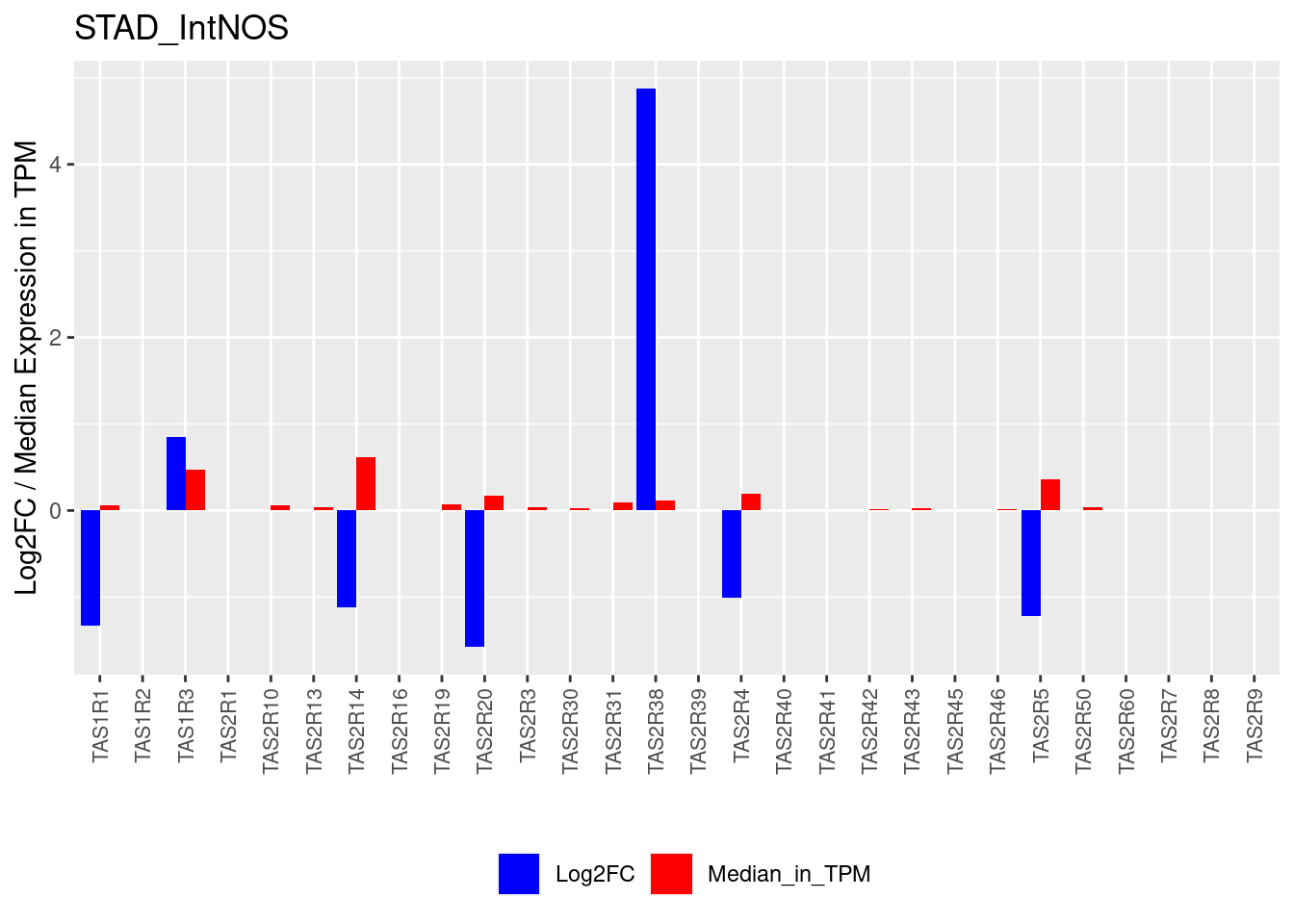

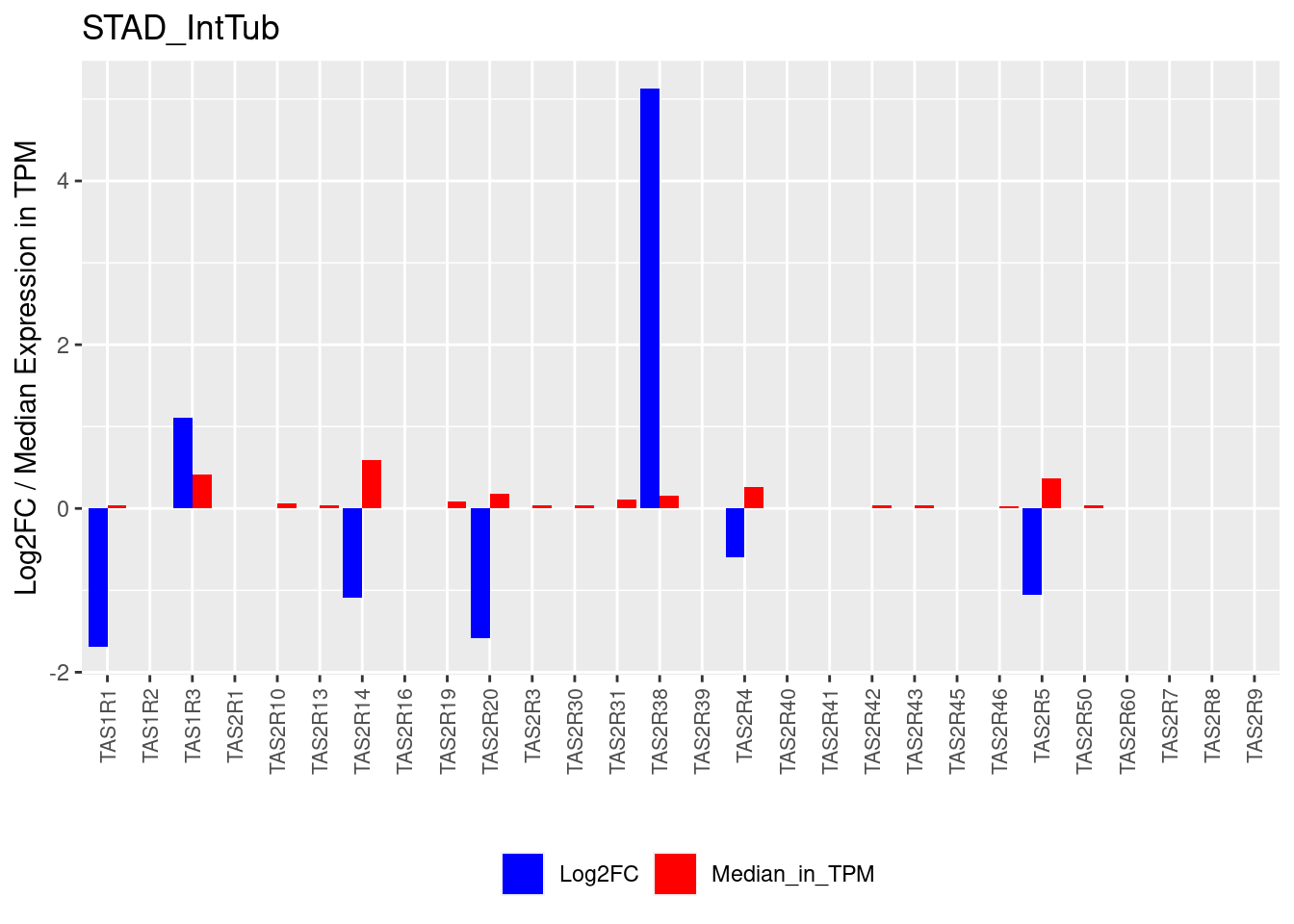

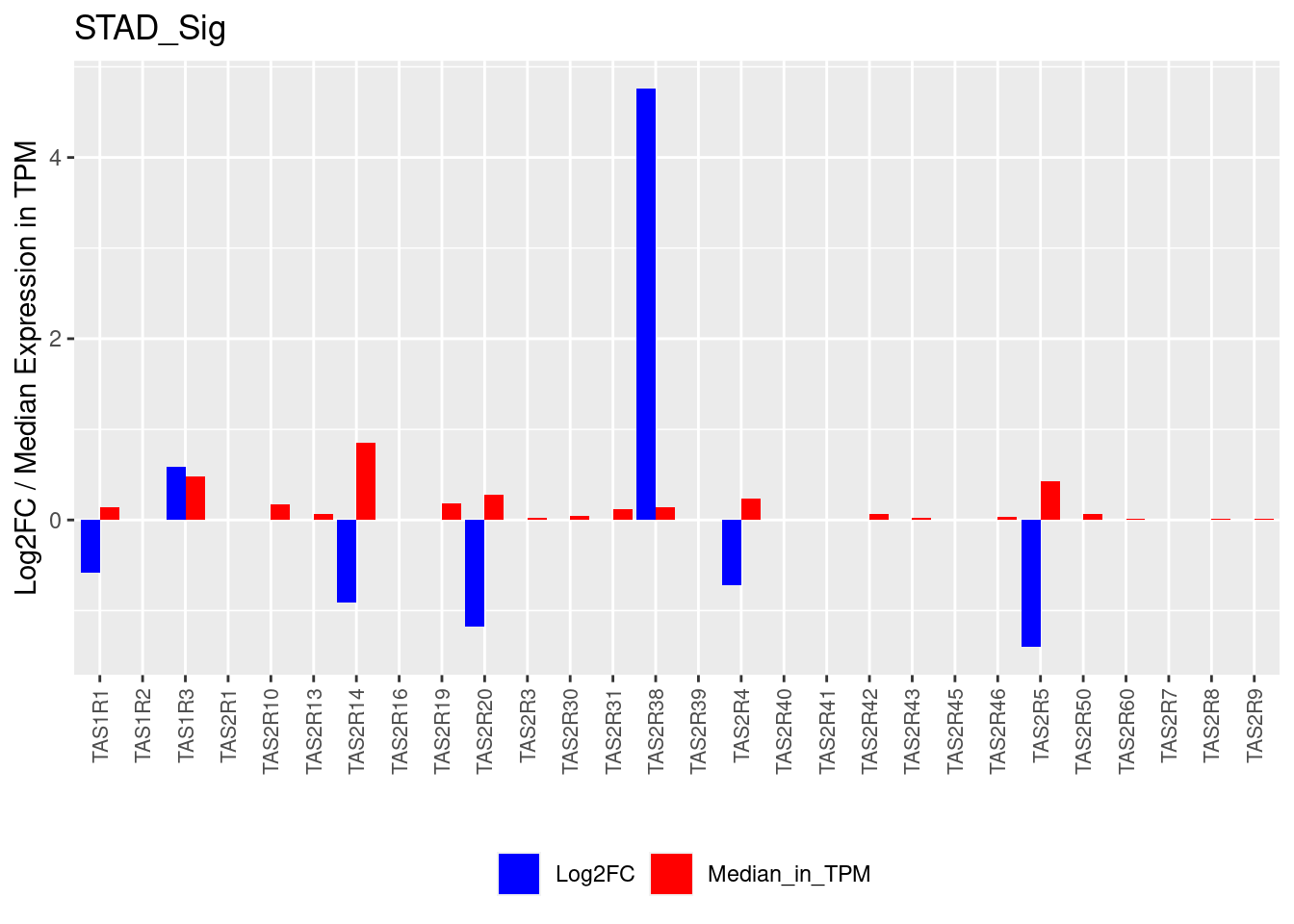

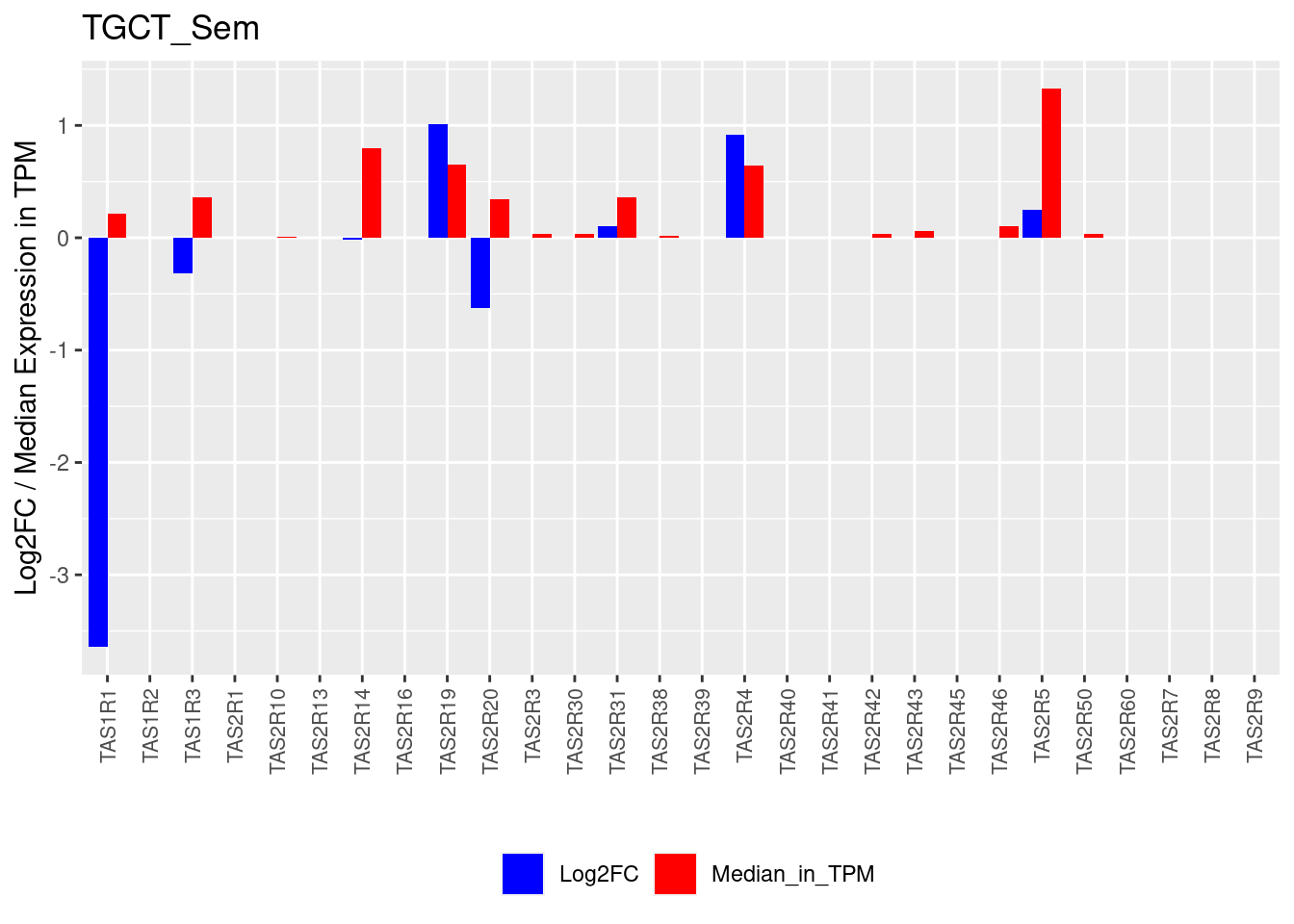

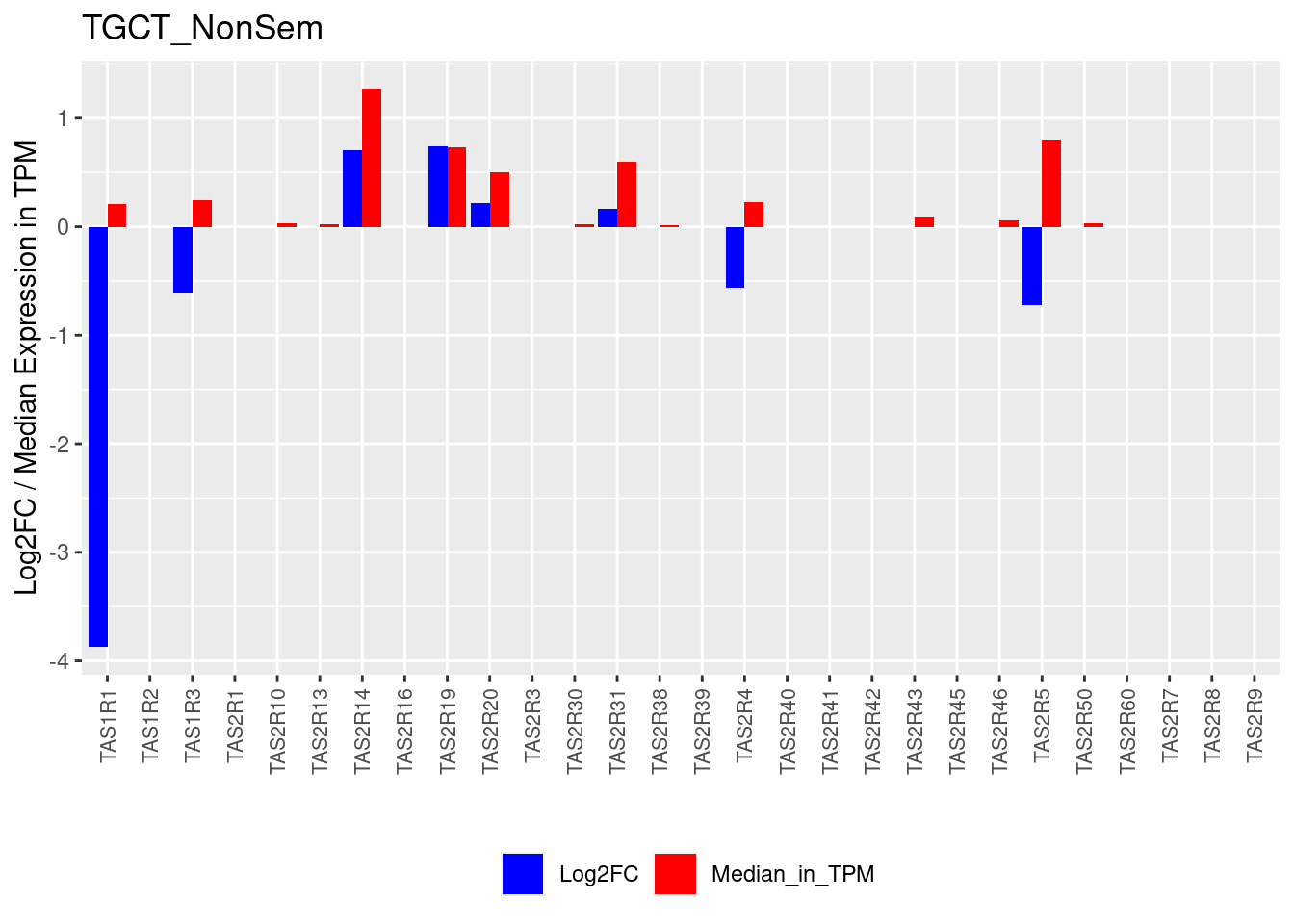

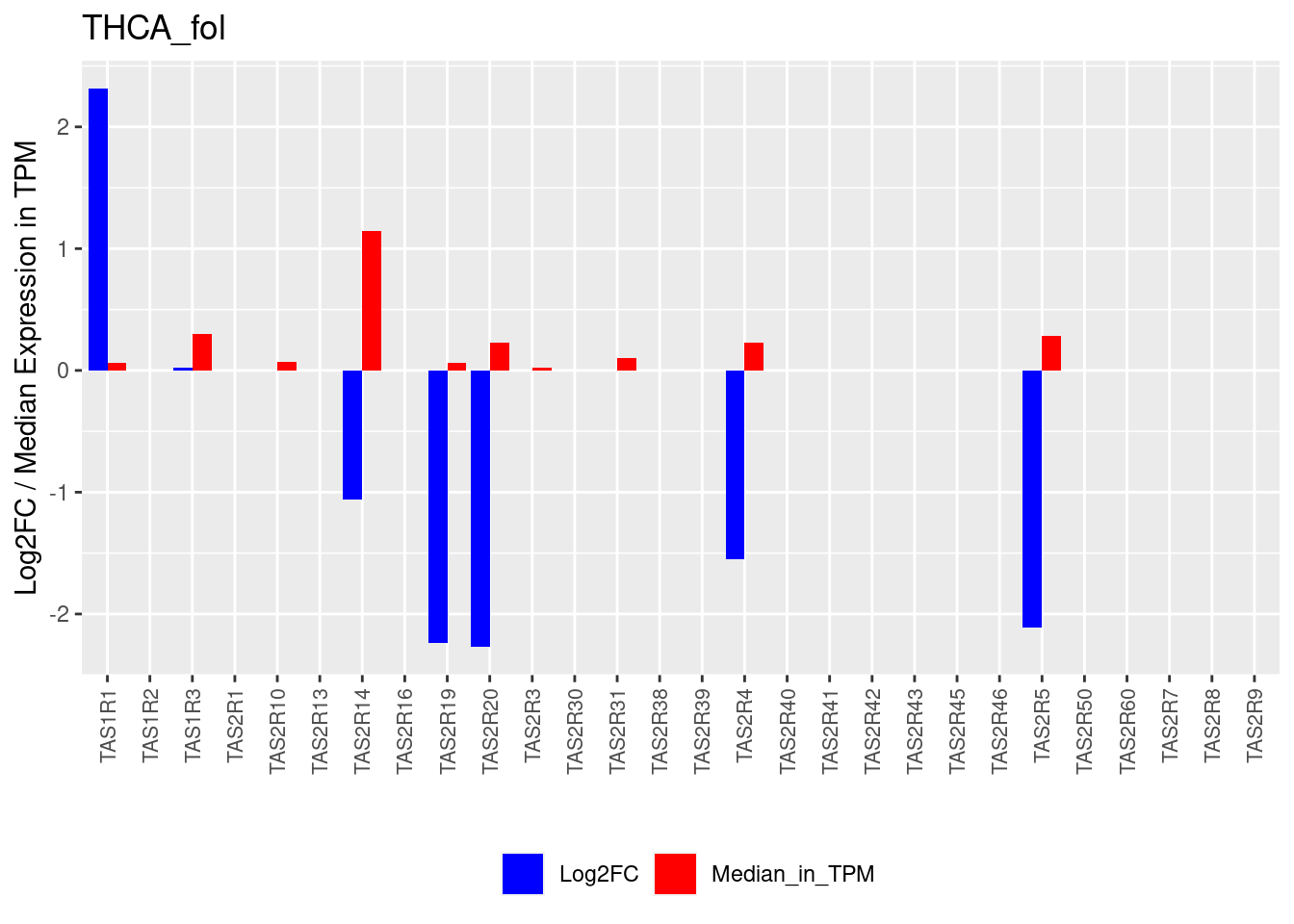


**Supplemental Figure 3.** *TAS1R* and *TAS2R* expression data shown as median expression in transcripts per million (TPM; red) for solid tumors and the log2 fold-change (log2FC; blue) for tumors compared to normal tissue. TPM, Transcripts Per Million; FC, fold change. Plots generated using files from <https://insellab.github.io/> as described in *Sriram et al*^47^ and labeled using The Cancer Genome Atlas (TCGA) cancer type and subclassification as defined in Figure 1A.

**Supplemental Figure 4. *TAS1R* and *TAS2R* mutation types in solid tumors.** Graph demonstrating the overall rate of different mutation types for *TAS1R* and *TAS2R* genes for different TCGA solid tumor types (n = 5,103 for 20 tumor types with data available). The rate was calculated by taking the total number of a given mutation type divided by the total number of samples. ACC, Adrenocortical Cancer; BLCA, bladder cancer; BRCA, breast cancer; CESC, Cervical Cancer; COAD, colon adenocarcinoma; ESCA, esophageal cancer; KICH, Kidney Chromophobe; KIRC, kidney clear cell carcinoma; KIRP, kidney papillary cell carcinoma; LIHC, liver hepatocellular carcinoma; LSQC, lung squamous cell carcinoma; LUAD, lung adenocarcinoma; OV, ovarian cancer; PAAD, pancreatic ductal adenocarcinoma; PRAD, prostate adenocarcinoma; SKCM, skin cutaneous melanoma; TCGA, The Cancer Genome Atlas; TGCT, testicular germ cell tumor, THCA, thyroid cancer; UCS, Uterine Carcinosarcoma.

**Supplemental Table 1. *TAS1R* and *TAS2R* expression is associated with survival in solid tumors.** Tumor samples were divided into high and low expression groups (defined as above-median and below-median expression, respectively) and the difference in mean survival days was calculated for these groups for each *TAS1R*/*TAS2R* gene and tumor. Negative survival difference indicates that higher gene expression corresponds to shorter survival times while positive survival difference indicates that higher gene expression corresponds to longer survival times. The table lists cancer subtypes with significant survival associations, the *TAS* genes, p-values, and mean survival differences. ACC, adrenocortical cancer; BLCA_NP, non-papillary bladder cancer; BRCA_IDC_HR+, hormone receptor positive infiltrating ductal carcinoma; CPM, counts per million; ESCA_AD, esophageal adenocarcinoma; KIRC, kidney clear cell carcinoma; KIRP, kidney papillary cell carcinoma; LUAD_NOSs, lung adenocarcinoma not otherwise specified; OV, ovarian cancer; SKCM_DistantMets, distantly metastatic skin melanoma; THCA_usual, thyroid cancer usual type.

**Supplemental Figure 5. Multiple *TAS2R*s are associated with survival in kidney clear cell carcinoma (KIRC).** Kaplan-Meier curves for KIRC high and low expression groups for (A) *TAS2R4*, (B) *TAS2R5*, (C) *TAS2R14*, (D) *TAS2R20*, and (E) *TAS1R3* and (F) the combined group of these genes together. Significance determined by Peto-Peto’s modified survival estimate. Combined expression analysis used a mean-normalized sum of expression of genes that individually predicted survival. CPM, counts per million; KIRC, kidney clear cell carcinoma.

**Supplemental Figure 6. Kaplan-Meier survival curves for expression of *TAS2R*s, *TAS1Rs,* and combined taste receptor gene groups.** Significance determined by Peto-Peto’s modified survival estimate. The Cancer Genome Atlas (TCGA) cancer types and sub-classifications defined in Figure 1A. CPM, counts per million.
